# Molecular Iodine-Catalyzed Synthesis of Imidazo[1,2-*a*]Pyridines: Screening of Their *In Silico* Selectivity, Binding Affinity to Biological Targets, and Density Functional Theory Studies Insight

**DOI:** 10.1021/acsomega.2c01570

**Published:** 2022-06-22

**Authors:** Deepika Geedkar, Ashok Kumar, Pratibha Sharma

**Affiliations:** School of Chemical Sciences, Devi Ahilya University, Indore 452001, Madhya Pradesh, India

## Abstract

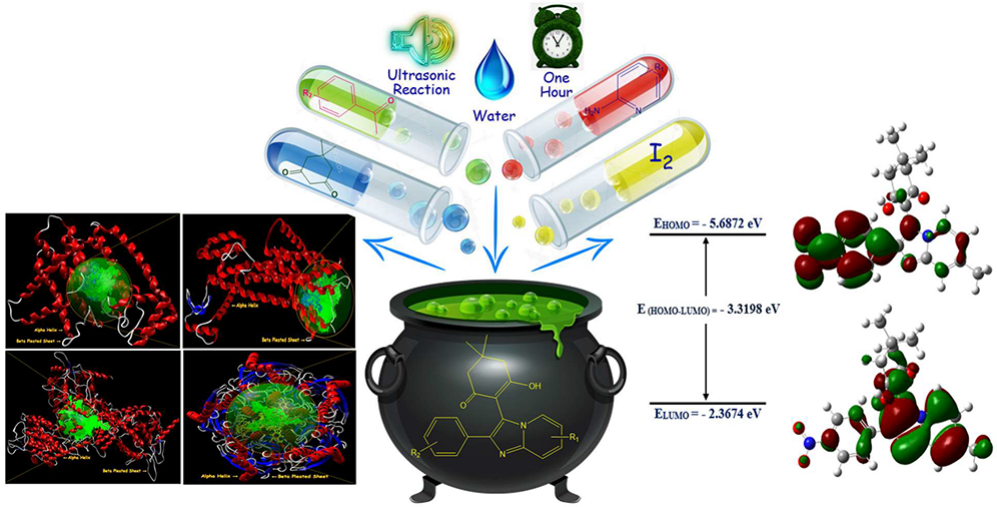

The present paper discloses an ultrasonication strategy assisted by molecular iodine as an environmentally benign catalyst leading to the synthesis of pharmacologically significant imidazo[1,2-*a*]pyridine scaffolds. The molecular-iodine-catalyzed approach for the synthesis of biologically active synthetic equivalents was achieved through three-component coupling embracing 2-aminopyridine derivatives, pertinent acetophenones, and dimedone in water medium under aerobic conditions. The higher product yield (up to 96%) with a miniature reaction time and modest catalyst loading as demonstrated by higher ecological compatibility and sustainability factors are fascinating features of this protocol. The structures of synthesized compounds were accomplished through FT-IR, ^1^H NMR,^13^C NMR, mass, and elemental analysis data. The virtual screening of synthetic moieties was performed to ascertain the *in silico* selectivity and binding affinities against several biological targets. Lipinski’s rules of five, ADMET, and TOPKAT descriptors were used to evaluate the drug-likeness assets. Furthermore, a quantum computational study was computed at the B3LYP/6-311G++(d,p) level of theory to investigate the density functional theory-based chemical reactivity parameters and HOMO–LUMO energy gap of the synthesized derivatives. The present studies open the way for *in vitro* and *in vivo* testing of synthesized derivatives as potent inhibitors with an improved pharmacological profile against farnesyl diphosphate synthase, phosphodiesterase III, CXCR4, and GABAa receptor agonists.

## 1 Introduction

Multicomponent reactions (MCRs) have been cherished in the realm of structural diversity and complexity in organic synthesis provisioning access to diverse sets of heterocyclic motifs.^1^ In a particular context, the imidazo-fused pyridines are one of the fascinating classes of nitrogen-fused heterocyclic scaffolds of versatile concern. Their chemistry has drawn significant attention over the past few years owing to their endowment in diverse medicinal applications, *viz*., antiviral, anticancer, anti-inflammatory, antibacterial, and anxiolytic agents.^2^

**Figure 1:**
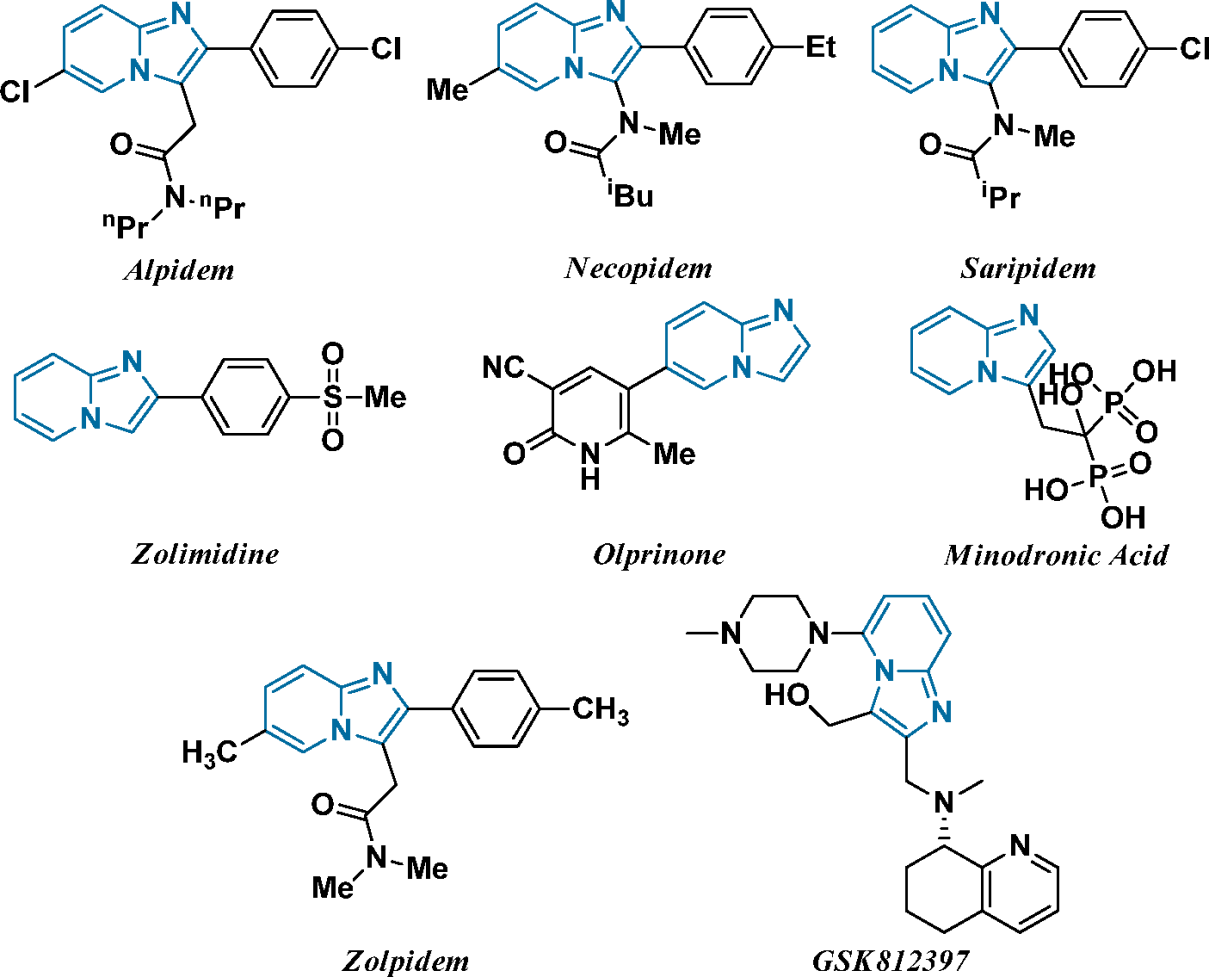
Some representative biologically active drugs embraced with an imidazole-fused pyridine skeleton.

Furthermore, several drugs embracing the imidazo-fused pyridine skeleton have been commercially marketed with their trade names as minodronic acid, a farnesyl diphosphate synthase inhibitor for the treatment of osteoporosis,^3^ olprinone,^4^ a phosphodiesterase III inhibitor as a cardiotonic agent for acute heart failure, zolpidem,^5^ a benzodiazepine γ-aminobutyric acid (GABA) receptor agonist for insomnia, zolimidine,^6^ for the treatment of peptic ulcers, alpidem, saripidem, and necopidem,^5,7^ a GABAa receptor agonist used as an anxiolytic agent, and some are under development, like GSK812397, a chemokine receptor, CXCR4 antagonist for the treatment of HIV,^8^ and β-amyloid formation inhibitors for Alzheimer’s disease^5,7^ as depicted in Figure 1. Further, the imidazo[1,2-*a*]pyridine derivatives have also been utilized as abnormal N-heterocyclic carbene ligands and have some noteworthy applications in material sciences and agrochemicals.^9,10^

Over the past decades, the synthesis of fused bicyclic imidazo[1,2-*a*]pyridines has elicited much consideration and resulted in the development of a variety of synthetic methodologies.^11^ In recent times, a number of multicomponent syntheses of imidazo[1,2-*a*]pyridines have been reported embracing assistance of a number of catalysts, *viz*., Cu(OTf)_2_, CuI/Cu(OTf)_2_, ionic liquid BPyBF_4_, FeCl_2_, FeCl_3_, Sc(OTf)_3_, ZrCl_2_, montmorillonite clay, InCl_3_, bromodimethyl sulfonium bromide (BDMS), copper(I) iodide-NaHSO_4_·SiO_2_, magnetic nano-Fe_3_O_4_-KHSO_4_·SiO_2_, CeCl_3_·7H_2_O/NaI, and dichloro(2-pyridinecarboxylato) gold [PicAuCl_2_].^11^ However, these methods endure some bottlenecks including the requirement of expensive and excessive amounts of catalysts, harsh reaction conditions, product diversity, and yields. Hence, keeping this in view, it was thought to be worthwhile to develop a simple and high-yielding environmentally benign protocol for the one-pot multicomponent synthesis of fused bicyclic imidazo[1,2-*a*]pyridine scaffolds.

Most organic reactions, including multicomponent transformations, use dimedone or its derivatives as a versatile synthon. The acidic feature of dimedone’s methylene group, which is in equilibrium with the tautomeric enol form, is responsible for its notoriety. These findings support the use of dimedone in a variety of organic processes that result in a variety of organic compounds with high medicinal potency. Their versatile chemistry with its low toxicity, easy accessibility and handling, moisture stability, and low cost make them pertinent precursors for the production of divergent organic compounds possessing anticancer, antioxidant, spasmolytic, anti-anaphylactic, and antibacterial activities. They have also emerged as a substantial class of compounds owing to their industrial and synthetic applications including several uses in dyes, fluorescent compounds, and laser technology. The organic transformations are performing on the basis of green chemistry procedures that are in demand over the last few decades.^12^

Because of accruing concerns over a hazardous sequel of organic solvents for the environment and living creatures, diverse types of MCRs have been effectively investigated in the aqueous medium. The reactions in an aqueous environment have spurred interest owing to its elite reactivity and selectivity that are onerous to acquire in conventional organic solvents. Water, probably because of its unique abilities, for instance, hydrogen bonding, high dielectric constant, and polarity, appears to be a more rational medium for organic transformation. In this framework, water is a preferential solvent providing an astonishing contribution to the field of organic synthesis.^13^

During recent decades, iodine has been evinced to be a versatile and benign reagent in the synthesis of a wide range of heterocyclic moieties including benzimidazoles,^14^ benzoxazoles, benzothiazole,^15^ quinolines,^16^ coumarins,^17^ and lactones.^18^ Molecular iodine has received escalating significance owing to its low outlay, nontoxicity, sustainability, ready availability, and ecofriendly properties as the preferable catalyst for organic synthesis. The utilization of iodine as a Lewis acid has improved substantially on account of its high tolerance to air as well as moisture and high catalytic activity in dilute and highly concentrated conditions.^19,20^

Computational approaches have become essential complements to each stage of the drug discovery and development trajectory. Molecular docking is an emphatic strategy to gain insight into the interactions between ligand and receptor in the design and development of the drug candidates. The significant role of molecular docking in the development of drug design is because of its ability to predict the best binding mode between drugs and the target protein. However, the density functional theory (DFT) method has also emerged as an influential technique for appraising the structural and spectral properties of organic compounds. The global and local chemical reactivity parameters and the impact of pertinent substituted groups on the synthesized scaffold were also achieved by utilizing the DFT method.^21^

**Scheme 1:**
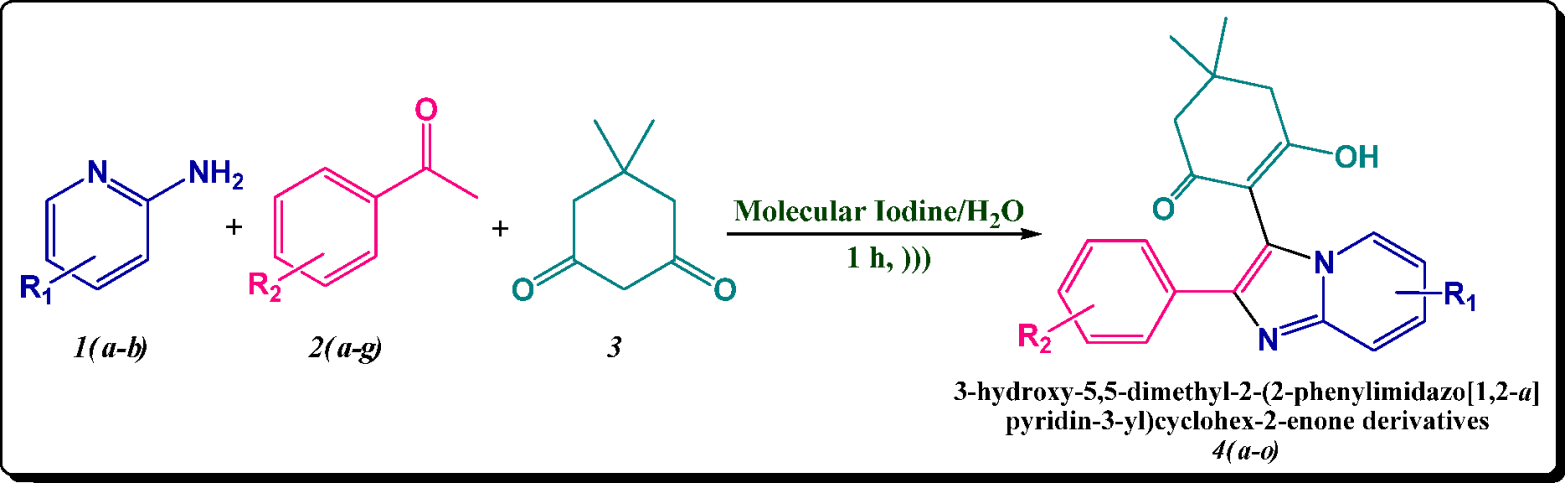
Ultrasonic-Assisted Synthesis of 2-Phenylimidazo[1,2-*a*]pyridin-3-yl Derivatives **4(a-o)**

Hence, encouraged by these advances, we sought to explore the relevance of these reagents and methods in the synthesis of fused imidazo-pyridine scaffolds. In continuation of our research program focused on the development of synthetic methodologies^22−24^ herein, we wish to disclose for the first time the ultrasonic-assisted synthesis of 2-phenylimidazo[1,2-*a*]pyridin-3-yl cyclohex-2-enones derivatives by employing iodine as a catalyst in aquatic conditions via one-pot MCR of 2-aminopyridine derivatives and pertinent aryl aldehydes along with dimedone embracing differently as one of the precursor substrates (Scheme 1). To the best of our knowledge, the use of dimedone as a precursor for the synthesis of imidazo[1,2-*a*]pyridine scaffolds by utilizing molecular iodine is hitherto unprecedented.

## 2 Experimental Section

### 2.1 General Procedure for the Ultrasonic-Assisted Synthesis of 2-Phenylimidazo[1,2-*a*]pyridin-3-yl Derivatives **4(a-o)**

A mixture of acetophenone derivatives **2(a-g)** (1.0 mmol) and a catalytic amount of (20 mol %) iodine in 4.0 mL of distilled water was irradiated utilizing an ultrasound at room temperature for 30 min. Then, 2-aminopyridine derivatives **1(a-b)** (1.0 mmol) and dimedone (**3**) (1.0 mmol) were added to the above mixture and again irradiated, employing ultrasound at room temperature for the ambient time (30 min). As the reaction time is very short, there was not a substantial elevation of temperature due to ultrasonic shock. The ultrasonic apparatus used showed the temperature automatically, so the temperature was controlled and fixed at room temperature by a water circulator in the case of any elevation of temperature.^22−24^ The progress of the reaction was monitored through TLC (on aluminum sheets precoated with silica) using *n*-hexane/ethyl acetate (4:1) as the eluting system. After the completion of the reaction, 15.0 mL of 10 mol % sodium thiosulfate solution was added and extracted with ethyl acetate (3 × 20 mL). The organic layer was dried over anhydrous Na_2_SO_4_ and concentrated under a vacuum. The crude product was filtered off and purified by recrystallization from methanol to afford the pure products **4(a-o)** (Scheme 1).

The analytical and spectroscopic data for each of the synthesized derivatives **4(a-o)** are summarized in Supporting Information.

### 2.2 Preparation of Protein and Ligand for Docking Study

The X-ray crystallographic structures of the human farnesyl diphosphate synthase (PDB ID: 5CG5),^25^ human phosphodiesterase 3B (PDB ID: 1SO2),^26^ human GABAa (PDB ID: 4COF),^27^ and CXCR4 (PDB ID: 3OE0)^28^ with a resolution of 1.40, 2.40, 2.97, and 2.90 Å, respectively, have been retrieved from the Research Collaboratory for Structural Bioinformatics–Protein Data Bank (RCSB–PDB). The imidazo[1,2-*a*]pyridin-3-yl derivatives **4(a-o)** were screened against human farnesyl diphosphate synthase, human phosphodiesterase 3B, human GABAa, and CXCR4 targets for the prediction of selectivity and binding affinity.

The Molegro Virtual Docker (MVD 2013.6.0.0 evaluation version)^29^ was used for performing docking studies, which are based on molecular docking (MD) simulations *viz*. ligand and macromolecular interaction energy. The docking score function (*E*_score_) is described by the following energy terms,
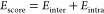
where *E*_inter_ is the ligand-protein interaction energy,

*E*_intra_ is the internal energy of the ligand
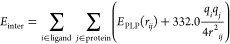
The summation considers all heavy atoms of the ligand and protein, wherein the cofactor atoms and water molecules have been taken into consideration if present, whereas the electrostatic interactions between charged atoms are considered by the second term.
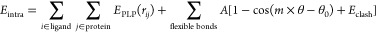
First, the geometrically optimized three-dimensional structures of imidazo[1,2-*a*]pyridin-3-yl derivatives **4(a-o)** were imported into the workspace of MVD accompanied by the individual target structure, retrieved from the RCSB Protein Data Bank for performing the molecular docking simulations. The geometrical optimizations of imidazo[1,2-*a*]pyridin-3-yl derivatives **4(a-o)** were procured by performing the molecular mechanics (MM2) and Hamiltonian approximation (AM1) optimizers until the root-mean-square (RMS) gradient value attains a value smaller than 0.001 kcal mol^–1^ Å^–1^. While the target protein was imported, all of the crystallographic water molecules were detached from it. Further, all the ligands and targets were refined with the protein preparation wizard extant at the preparation window in the workspace of MVD, followed by the identification and detection of active sites (cavities) within the target protein.

During this computational process, the maximum numbers of cavities were set to five, the grid resolution to 0.80 Å, and the probe size to 1.2 Å, while the other parameters were taken as default.^30^ The docking scores indicate the significant binding interactions, i.e., hydrogen-bonding and steric interactions that take place between the ligand of different conformations and key amino acid residues in the binding pocket of the target. Furthermore, the Mol Dock score is the sum of internal ligand energies, protein interaction energies, and soft penalties. The protein–ligand energy is the total interaction energy between the ligand and the target molecule, whereas the steric score indicates the interaction energy between the ligand and protein. The H-bond score is the hydrogen-bonding energy between the protein and ligand. The reranking score function is computationally more valuable than the scoring function used during the docking simulations. In general, the rerank score is better than the docking score function for determining the best pose among the various poses derived from the identical ligand.^30,31^

**Figure 2:**
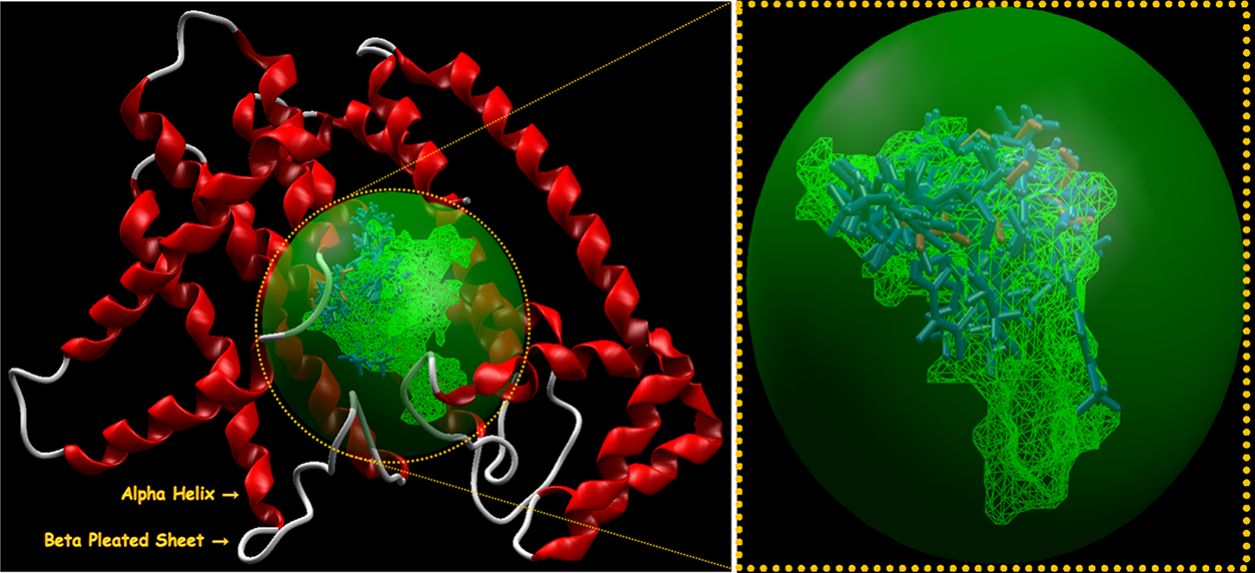
Secondary structure of farnesyl diphosphate synthase binds with imidazo[1,2-*a*]pyridin-3-yl derivatives **4(a-o)** (blue) and standard drug minodronic acid (pink) inside the cavity (green framework).

The active sites or cavities (1–5) with diverse surface areas and volumes within the selected targets for screening are depicted in Table S5 of the Supporting Information using the detect cavity module in MVD.^31^ The secondary structures of the selected targets with detected active sites (1–5) were visualized using Molegro Virtual Docker^29^ and are presented in Figure 1S of the Supporting Information. The MD simulations were performed within the cavity of the larger surface area of protein. Some other parameters such as binding radius, grid resolution, and maximum iteration parameters were set to 15 Å, 0.3 Å, and 1500, respectively. The docking algorithm was set to MolDock Simplex Evolution (MolDock SE) docking algorithm with a population size of 50. For cluster similar poses and ignore similar poses (for multiple runs only), the RMSD thresholds were firm to 1.00 Å. The number of independent runs was retained as 10, and each of these runs was recurred to a single final solution (Pose). After the completion of docking simulations, only the negative lowest-energy representative cluster was returned from each of them, followed by the removal of all the similar poses and keeping the best scoring pose. The clusters were ranked in order of increasing the lowest binding energy conformation in each cluster. The analyses of the molecular docking results were performed on the first binding free energy pose with minimum energy.^30,31^ The secondary structures of the selected targets bind with imidazo[1,2-*a*]pyridin-3-yl derivatives **4(a-o)** (blue) and standard drugs (pink) inside the cavities (green framework) envisaged by Molegro Virtual Docker (MVD 2013.6.0.0 evaluation version)^28^ and illustrated in Figures 2, 4, 6, and 8, respectively.

### 2.3 Prediction of ADMET, Toxicity, and Drug-Likeness Properties

The drug-likeness of all of the synthesized compounds **4(a-o)** and standard drugs were evaluated by assessing Lipinski’s rules, absorption, distribution, metabolism, excretion, and toxicity (ADMET) descriptors, and TOPKAT descriptors utilizing Accelrys Discovery studio package.^32^ The ADMET analyses were achieved using some descriptors, for instance, absorption, solubility, atom-based Log P98 (ALogP98), ADME 2D polar surface area (ADME 2D PSA), blood–brain barrier (BBB), cytochrome P4502D6 (CYP2D6), and hepatotoxicity (HEPATOX), and plasma protein binding (PPB). Furthermore, the Toxicity Prediction by Komputer-Assisted Technology (TOPKAT) analyses were attained using carcinogenic potency of the National Toxicology Program (NTP) Carcinogenicity of Male Rat and Mouse, Ames Mutagenicity, Skin Irritancy, Aerobic Biodegradability.^33^

### 2.4 DFT-Based Chemical Reactivity Parameters

All the molecular structures of imidazo[1,2-*a*]pyridin-3-yl derivatives **4(a-o)** were optimized at the B3LYP level of theory using the 6-311G++(d,p) basis set of the Gaussian 09 program suite.^34^ The global chemical reactivity parameters consist of total energy, electrophilicity (ω), chemical hardness (η), and electronic chemical potential (μ). The stability and reactivity of the molecules are appraised by these parameters.^35^ The calculated results were obtained using the HOMO and LUMO energies according to Koopmans’ theorem and Parr approximation.^36,50^
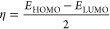
The HOMO–LUMO energy gap is used for assessing the chemical hardness as well as for determining the stability of the molecular structure.^36^ It is recognized that the higher the HOMO–LUMO energy gap, the more stable and chemically harder the molecules in contrast to the softer and less stable molecules. The parameter electrophilicity index (ω) is delineated as the lowering in energy of molecules due to the flow of electrons from the highest occupied molecular orbital to the lowest unoccupied molecular orbital. Hence, it measures the energy changes that take place when a molecule is saturated by the addition of electrons and governs the chemical reactivity behavior of the molecules.^37,38^ The following equation represents the electrophilicity index (ω) of molecules as follows,
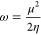
Electronic chemical potential (μ) is described as the negative of electronegativity and is given by the equation as^36^
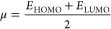
where η represents chemical hardness, ω represents electrophilicity index of molecules, and μ represents the electronic chemical potential.

It depicts the transfer of charge that takes place within the molecule in the ground state which describes the tendency of electrons to escape from the equilibrium state. Hence, chemically reactive molecules show a greater chemical potential.

The condensed Fukui functions (FF) were utilized to rationalize and predict the reactivity profile of the molecule, which were evaluated on the basis of atomic charges obtained from electron density population analysis using the following equations,^39^
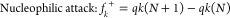

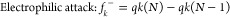

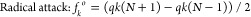
where *qk* (*N*) is the charge on the atom *k* for (*N*) total electrons, *qk* (*N+*1) is the charge on the atom *k* for (*N +* 1) total electrons, *qk* (*N* – 1) is the charge on the atom *k* for (*N –* 1) total electrons, respectively.

Here, the highest positive values of the Fukui function indicate the most probable atomic sites for the nucleophilic (*f*_*k*_^+^), electrophilic (*f*_*k*_^–^), and radical attacks (*f*_*k*_^0^).^40^

### 2.5 Data and Software Availability

The X-ray crystallographic structures of the biological targets were retrieved from the Research Collaboratory for Structural Bioinformatics–Protein Data Bank (RCSB–PDB). All of the chemical structures were produced from ChemDraw Ultra 11.0. The docking studies were performed adopting the Molegro Virtual Docker (MVD 2013.6.0.0 evaluation version). All of the quantum computational studies were computed at the B3LYP level of theory using the 6-31G++(d,p) basis set of the Gaussian 09 program suite. The drug-likeness properties were analyzed by assessing Lipinski’s rules, absorption, distribution, metabolism, excretion, and toxicity (ADMET) descriptors, and TOPKAT descriptors utilizing the Accelrys Biovia Discovery Studio 2019 package. The full workflow is reported in the Experimental Section.

## 3 Results and Discussion

### 3.1 Optimization of Reaction Conditions

We have developed a green and efficient methodology for the preparation of tetrasubstituted imidazole-fused pyridine tethered with dimedone via readily available reactants. We envisaged that the synthesis of our desired products was attained from the condensation reaction of phenylglyoxal derivatives, pertinent 2-aminopyridines, and dimedone. After considering the expenses and limited commercial accessibility of phenylglyoxal derivatives, we prepared *in situ* phenylglyoxals from the phenyl methyl ketones in the presence of iodine on the basis of inference revealed from the literature.

To optimize the reaction conditions, a model condensation reaction of 2-aminopyridine **1(a)**, acetophenone **2(a)**, and dimedone **(3)** was performed in the presence and absence of varying catalysts and solvents under ultrasonic conditions. The initial reactions were carried out in the absence of an iodine source but did not result in our desired three-component product **4(a)** in both neat and water media even after 2 h (Table 1, entries 1 and 2). To explore the impact of varying iodine sources, the reaction was performed in the presence of sodium iodide, potassium iodide, copper iodide, and zinc iodide for 1 h, but the reaction did not proceed with substantial yields (Table 1, entries 3–6). It is noted from the data presented in Table 1 that the reaction of iodine in the water medium was found to be in a suitable condition to afford a trace amount of product in 79% yield, while in the absence of water the medium had moderate performance to provide the product in a 62% yield even after one and half hours (Table 1, entries 7, 8).

**Table 1:** Optimization of Reaction Conditions for the Sonochemical Synthesis of 3-Hydroxy-5,5-dimethyl-2-(2-phenylimidazo[1,2-*a*]pyridin-3-yl)cyclohex-2-enone **4(a)**^a^

entry	source of iodine	solvent	time (h)^b^	yield^c^
**1**	−	−	2	NR
**2**	−	H_2_O	2	NR
**3**	NaI	H_2_O	1	28
**4**	KI	H_2_O	1	33
**5**	CuI	H_2_O	1	55
**6**	ZnI_2_	H_2_O	1	47
**7**	I_2_ (15 mol %)	−	1.5	62
**8**	I_2_ (15 mol %)	H_2_O	1	79
**9**	**I**_**2**_**(20**mol %)	**H**_**2**_**O**	**1**	**84**
**10**	I_2_ (20 mol %)	H_2_O	1.5	84
**11**	I_2_ (25 mol %)	H_2_O	1	83

a Reaction conditions: 2-aminopyridine (1 mmol) **1(a)**, acetophenone (1 mmol) **2(a)**, and dimedone (1 mmol) **(3)**.

b All the reactions were monitored by TLC.

c Isolated yield.

After ascertaining the optimal reaction conditions, we found that in the presence of 20 mol % of iodine underwater medium the reaction proceeds efficiently to afford the product in a preeminent yield (Table 1, entry 9). Intriguingly, the reaction was also executed for one and a half hours, but no further improvement in the yield of the product was acquired (Table 1, entry 10). A high amount of catalytic loading (25 mol %) under similar conditions revealed that there was no significant enhancement in the yield or in the reaction time (Table 1, entry 11). The results are summarized in Table 1.

In order to explore the generality and scope of this iodine-mediated multicomponent reaction, a wide variety of pertinent acetophenone, 2-amino pyridine derivatives, and dimedone was reacted under the optimized conditions, and the results are summarized in Table 2. Intriguingly, the 2-aminopyridine and derivative tethered with methyl at the para position were reacted under the optimized reactions, and in all of the cases significant yields were obtained. The variability of acetophenone derivatives having substituents such as 4-Cl, 2-OH, 4-OH, 4-OMe, 4-Br, 4-NO_2_, and 3-NO_2_ was found suitable for the synthesis of the corresponding imidazole-fused pyridine-3yl tethered with dimedone scaffolds. It should be noted that the acetophenone substituted with electron-donating groups resulted in better yields as compared to electron-withdrawing groups. It is noteworthy to mention that under the given reaction conditions; we did not observe any iodination in the aromatic rings of products **4(a-o)**. Also, the benzylic CH_2_ of the indene was unaffected by the iodine-mediated oxidation process.

**Table 2:**
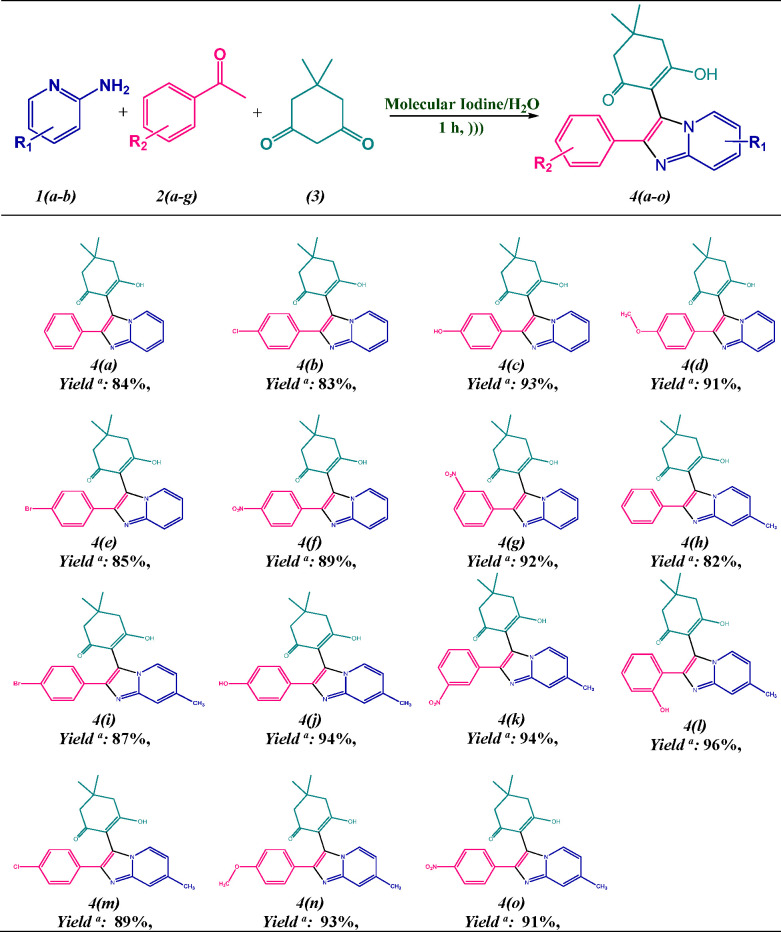
Substrate Scope for the Ultrasonic-Assisted Synthesis of 2-Phenylimidazo[1,2-*a*]pyridin-3-yl)cyclohex-2-enones Derivatives **4(a-o)**^a^

a Reaction conditions: 2-aminopyridine derivatives (1 mmol) **1(a-b)**, pertinent acetophenone (1 mmol) **2(a-g)**, dimedone (1 mmol) **(3)**, and a catalytic amount (20 mol %) of I_2_ was irradiated for 1 h in the presence of water. Reactants **2(a-g)** and **(3)** were added to the reaction mixture only after the disappearance of the reactant **1(a-b)**.

b Isolated yield.

To check the feasibility of scale-up and efficiency of this protocol, gram-scale synthesis of 3-hydroxy-2-(2-(4-methoxyphenyl)imidazo[1,2-*a*]pyridin-3-yl)-5,5-dimethylcyclohex-2-enone **4(d)** was carried out under optimized reaction conditions. The condensation reaction of imidazole-fused pyridin-3yl substrate in the 5 mmol scale resulted in the corresponding yield of 1.757 g with 91% yield.

**Scheme 2:**
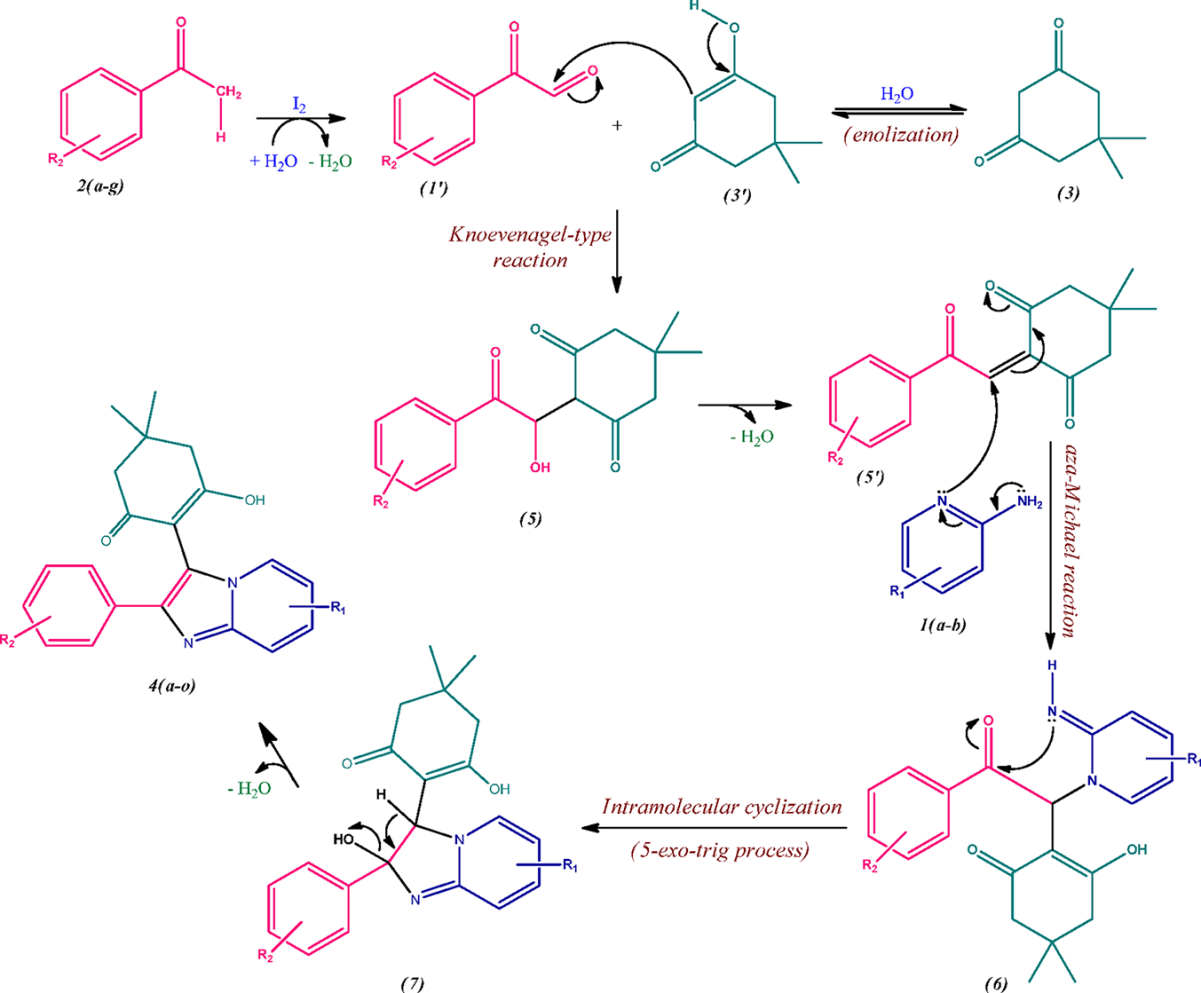
Plausible Mechanistic Pathway for the Synthesis of 2-Phenylimidazo[1,2-*a*]pyridin-3-yl)cyclohex-2-enones Scaffolds **4(a-o)**

On the basis of inference revealed from the literature and resultant outcomes,^41,42^ a plausible mechanistic pathway for the molecular iodine-catalyzed synthesis of imidazo[1,2-*a*]pyridin-3-yl derivatives **4(a-o)** is depicted in Scheme 2. The cascade reaction began with the attack of molecular iodine on acetophenone derivatives **2(a-g)** in an aqueous medium followed by dehydration which resulted *in situ* generation of phenylglyoxal **(1′)**. The Knoevenagel-type reaction takes place between phenylglyoxal **(1′)** and enolic form **(3′)** of dimedone **(3)** to form an intermediate **(5′)** followed by the elimination of water molecule. Furthermore, the 2-aminopyridine **1(a-b)** undergoes aza-Michael addition to form adduct (**6)**, which subsequently undergoes intramolecular ring closure via an energetically favored 5-*exo*-*trig* process, thereby resulting in the cycloadduct **(7)** followed by removal of water that furnishes the desired product **4(a-o)**. The whole process involves the elimination of three molecules of water as a greener waste.

Iodine can act as either a Lewis acid or as a source of *in situ* HI in organic transformations. To be acquainted with the concrete function of iodine via a three-component reaction, a few additional experiments were conducted. The model condensation reaction of 2-aminopyridine **1(a)**, acetophenone **2(a)**, and dimedone (**3)** was performed using 20 mol % HI in water media to afford the product **4(a)** in a moderate yield of 67%. To abandon the role of *in situ* HI, we executed a further reaction in the presence of iodine (20 mol %) along with 20 mol % of sodium hydrogen carbonate under aqueous media in similar reaction conditions. Interestingly, this resulted in 88% of the tetrasubstituted imidazo[1,2-*a*]pyridin-3-yl linked with a dimedone ring **4(a)**. It is noteworthy to mention that, if the reaction is catalyzed by HI in the presence of an equimolar amount of base, it will nullify the acid and have a drastic effect on the yield of the desired product. Since in our protocol this did not happen, we believe that iodine is acting as a Lewis acid to activate the carbonyl group (CO) in all the steps involves in a plausible mechanism as shown in Scheme 2.

### 3.2 Molecular Docking Studies

The secondary structures of farnesyl diphosphate synthase and the phosphodiesterase 3B target with the detected active sites are presented in Figures 2and 4, respectively. The screening results against the farnesyl diphosphate synthase target showed that compound **4(k)** exhibited the highest MolDock score (−145.600), rerank score (−107.580), and protein–ligand interaction (−149.188) among the series, and a comparable steric score (−136.328) with compound **4(g)** demonstrating the highest steric score (−139.207) among the series. In comparison with the reference standard minodronic acid, all compounds in the series demonstrated a better MolDock score, protein–ligand energy, and steric scores against farnesyl diphosphate synthase as summarized in Table 3.

**Table 3:** Docking Scores^a^ of Imidazo[1,2-*a*]pyridin-3-yl Derivatives **4(a-o)** Docked with Farnesyl Diphosphate Synthase Target Selected for Screening

compound name	MolDock score	rerank score (kJ/mol)	interaction energy (kJ/mol)	steric	HBond (kJ/mol)
**4(a)**	–121.070	–46.2331	–129.523	–125.412	–4.12665
**4(b)**	–116.882	–86.3746	–127.038	–123.787	–3.25102
**4(c)**	–119.894	–92.4212	–131.989	–126.252	–5.73747
**4(d)**	–121.378	–92.2706	–133.879	–130.228	–3.65058
**4(e)**	–116.694	–86.3413	–126.942	–123.758	–3.18489
**4(f)**	–124.997	–82.7741	–139.449	–130.422	–11.7324
**4(g)**	–136.382	–97.9987	–148.481	**–139.207**	–9.27341
**4(h)**	–121.380	–91.9035	–131.591	–129.897	–5.74383
**4(i)**	–115.326	–78.1772	–126.461	–119.791	–6.66971
**4(j)**	–123.665	–94.6358	–134.444	–128.501	–5.94368
**4(k)**	**–145.600**	**–107.580**	**–149.188**	–136.328	**–12.8601**
**4(l)**	–126.914	–97.9745	–138.142	–133.722	–8.41212
**4(m)**	–117.557	–89.4801	–128.437	–124.994	–3.44365
**4(n)**	–125.239	–94.7880	–136.104	–132.444	–3.65975
**4(o)**	–125.450	–87.6107	–135.379	–124.855	–10.5236
minodronic acid	–111.023	–88.7053	–117.088	–97.5360	**–19.5515**

a MolDock score, rerank score, protein–ligand interaction, H-bond, and steric score.

**Figure 3:**
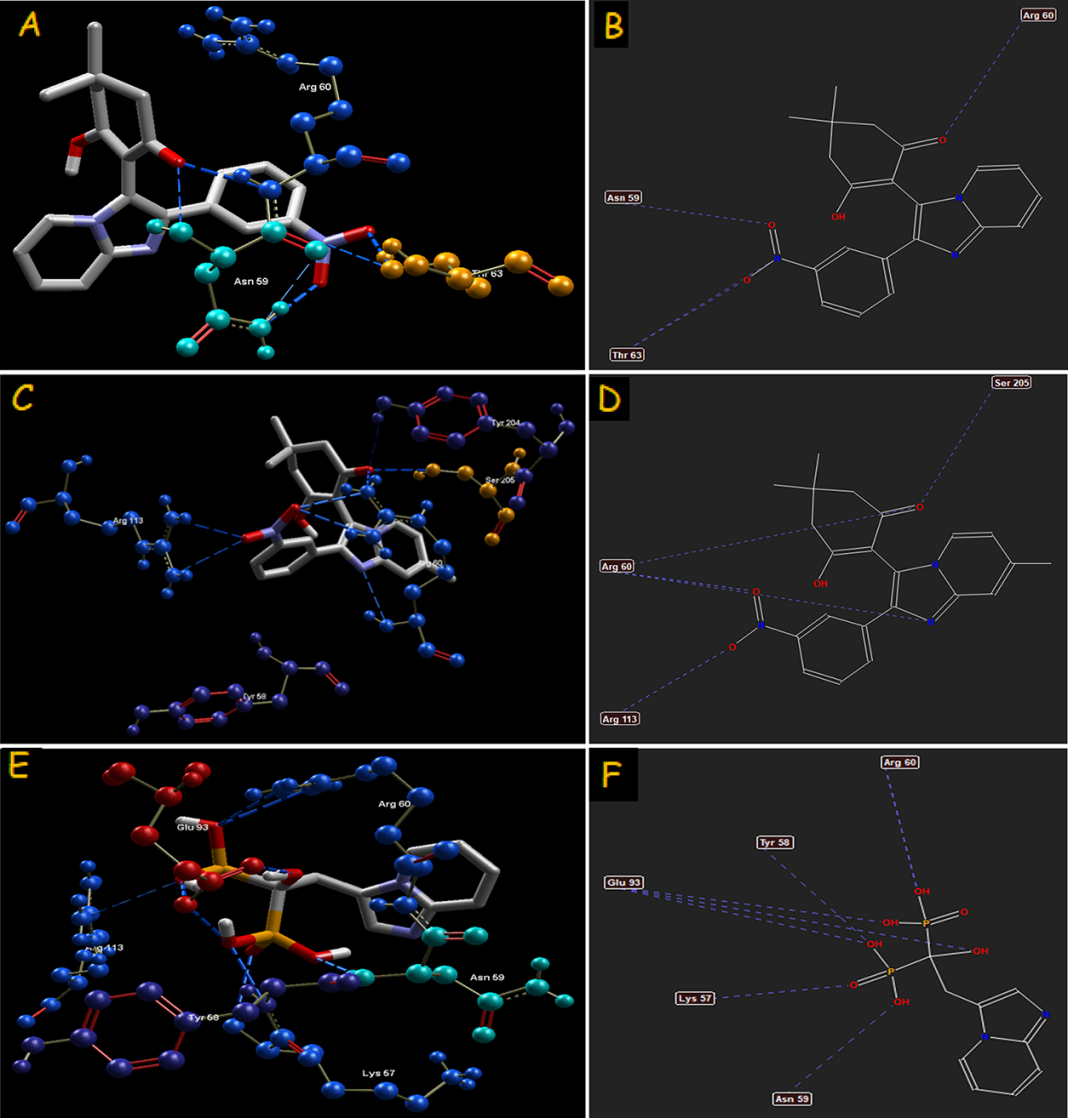
Hydrogen-bond interaction of compounds **4(k)** (A–B), **4(g)** (C–D), and standard drug minodronic acid (E–F) with farnesyl diphosphate synthase target.

**Figure 4:**
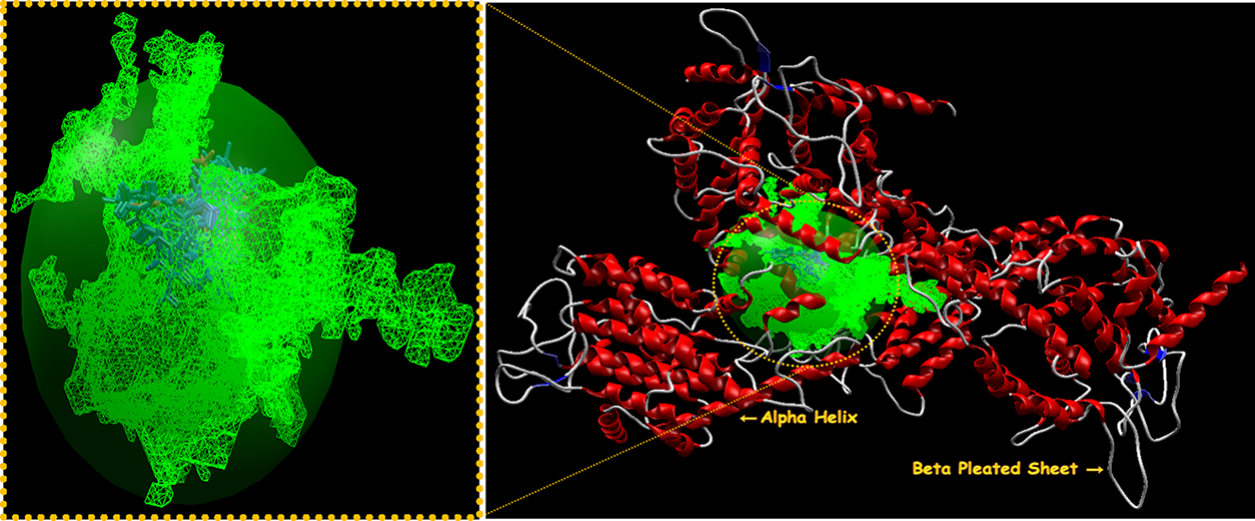
Secondary structure of phosphodiesterase 3B binds with imidazo[1,2-*a*]pyridin-3-yl derivatives **4(a-o)** (blue) and standard drug olprinone (pink) inside the cavity (green framework).

The compounds **4(k)**, **4(g)**, and **4(l)** with the highest moldock, rerank scores, steric scores, and protein–ligand energy reveal that the substitutions on the aromatic ring by nitro and hydroxyl groups at the meta and ortho positions respectively show significant binding affinity with the farnesyl diphosphate synthase target. The hydrogen-bond interactions, bond length, and bond energies of imidazo[1,2-*a*]pyridin-3-yl derivatives and standard drug minodronic acid are depicted in Table 4. All of the compounds and minodronic acid shows H-bond interactions with Tyr 58, Asn 59, Arg 60, Thr 63, Glu 93, Arg 113, Tyr 204, and Ser 205 of the farnesyl diphosphate synthase target. The reference drug exhibits the highest H-bond interactions followed by compound **4(k)** (−19.5515 and −12.8601, respectively).

**Table 4:** Molecular Interactions Analyses of Imidazo[1,2-*a*]pyridin-3-yl Derivatives **4(a-o)** and Standard Drug with Farnesyl Diphosphate Synthase Target

compound name	interaction	bond energy(kJ/mol)	bond length (Å)
**4(a)**	Ser 205 (O)–N (11)	–1.61890	3.27622
	Ser 205 (O)–N (12)	–2.50000	2.61902
	Arg 60 (N)–O (24)	–0.00775	2.29911
**4(b)**	Tyr 58 (N)–O (23)	–0.01189	3.57803
	Asn 59 (N)–O (23)	–0.73913	2.59207
	Arg 60 (N)–O (23)	–2.50000	2.82512
**4(c)**	Tyr 58 (N)–O (23)	–0.10649	3.44088
	Asn 59 (N)–O (23)	–1.08913	2.58761
	Arg 60 (N)–O (23)	–2.50000	3.09408
	Ser 205 (O)–O (25)	–2.04184	2.54502
**4(d)**	Tyr 58 (N)–O (23)	–0.09476	3.44893
	Asn 59 (N)–O (23)	–1.06219	2.56740
	Arg 60 (N)–O (23)	–2.49363	3.10127
**4(e)**	Tyr 58 (N)–O (23)	–0.02610	3.54831
	Asn 59 (N)–O (23)	–0.65880	2.55617
	Arg 60 (N)–O (23)	–2.50000	2.82583
**4(f)**	Ser 205 (O)–N (12)	–2.50000	3.00863
	Arg 60 (N)–O (24)	–0.20808	3.15632
	Arg 60 (N)–O (24)	–1.35251	2.14446
	Asn 59 (N)–N (25)	–0.25464	3.54907
	Thr 63 (O)–N (25)	–2.41718	3.11656
	Thr 63 (O)–O (26)	–2.50000	2.60213
	Asn 59 (N)–O (27)	–2.50000	2.60025
**4(g)**	Asn 59 (N)–O (23)	–0.57469	2.64989
	Arg 60 (N)–O (23)	–2.50000	2.61028
	Asn 59 (N)–N (25)	–0.09783	3.58043
	Thr 63 (O)–N (25)	–1.10088	3.37982
	Asn 59 (N)–O (26)	–2.50000	2.60122
	Thr 63 (O)–O (27)	–2.50000	2.80632
**4(h)**	Arg 60 (N)–N (12)	–1.49341	3.04388
	Arg 60 (N)–O (24)	–0.84816	3.12660
	Arg 60 (N)–O (24)	–2.02485	2.06714
	Tyr 204 (O)–O (24)	–1.20691	3.35862
	Ser 205 (O)–O (24)	–0.17050	2.32046-
**4(i)**	Ser 205 (O)–N (12)	–2.50000	2.88372
	Arg 60 (N)–O (24)	–2.22715	3.10029
	Glu 93 (O)–O (24)	–1.94256	3.21149
**4(j)**	Tyr 58 (N)–O (23)	–0.05245	3.51506
	Asn 59 (N)–O (23)	–0.99386	2.59110
	Arg 60 (N)–O (23)	–2.50000	2.99640
	Ser 205 (O)–O (26)	–2.39738	2.58769
**4(k)**	Arg 60 (N)–N (12)	–1.36329	2.79244
	Arg 60 (N)–O (23)	–1.81084	2.55609
	Tyr 204 (O)–O (23)	–0.26226	3.54755
	Ser 205 (O)–O (23)	–2.49869	2.59998
	Arg 60 (N)–O (27)	–2.41387	3.09897
	Arg 60 (N)–O (27)	–2.34329	3.12369
	Arg 113 (N)–O (28)	–1.17171	2.90942
	Arg 113 (N)–O (28)	–0.99612	3.11867
**4(l)**	Arg 60 (N)–N (12)	–1.53196	3.08287
	Arg 60 (N)–O (24)	–0.85461	3.12642
	Arg 60 (N)–O (24)	–1.99573	2.07049
	Tyr 204 (O)–O (24)	–1.22651	3.35470
	Ser 205 (O)–O (24)	–0.11522	2.31383
	Tyr 58 (N)–O (26)	–0.20459	3.10284
	Asn 59 (N)–O (26)	–2.48350	2.75736
**4(m)**	Tyr 58 (N)–O (23)	–0.01605	3.57054
	Asn 59 (N)–O (23)	–0.92761	2.57293
	Arg 60 (N)–O (23)	–2.50000	2.94743
**4(n)**	Tyr 58 (N)–O (23)	–0.08319	3.46818
	Asn 59 (N)–O (23)	–1.07656	2.57721
	Arg 60 (N)–O (23)	–2.50000	3.08395
**4(o)**	Arg 60 (N)–N (12)	–2.48607	3.08471
	Arg 113 (N)–O (23)	–1.80969	2.61959
	Tyr 58 (N)–N (26)	–1.66976	3.21718
	Asn 59 (N)–O (27)	–2.05854	3.09542
	Tyr 58 (N)–O (28)	–2.49951	2.71332
minodronic acid	Glu 93 (O)–O (11)	–2.50000	2.62058
	Arg 60 (N)–O (15)	–2.20851	3.05595
	Arg 60 (N)–O (15)	–2.22001	3.09973
	Glu 93 (O)–O (16)	–2.50000	2.64215
	Arg 113 (N)–O (16)	–0.35967	3.26586
	Tyr 58 (N)–O (17)	–2.40243	2.62664
	Glu 93 (O)–O (17)	–2.50000	2.70066
	Arg 60 (N)–O (15)	–2.36085	3.09912
	Asn 59 (N)–O (19)	–2.50000	2.60019

It is inferred from Table 5 that, among the imidazo[1,2-*a*]pyridin-3-yl derivatives, compound **4(g)** showed the highest MolDock (−130.663), rerank (−98.323), protein–ligand energy (−141.452), and steric (−132.962) scores against phosphodiesterase 3B. All of the compounds in the series exhibited a greater MolDock score, protein–ligand energy, and steric score when compared with the reference standard olprinone. The rerank scores of the remaining compounds in the series except for **4(a)**, **4(b)**, **4(d)**, **4(e)**, **4(h)**, **4(k)**, and **4(l)** are also better than that of olprinone. It should be noted from Table 5 that the presence of substitutions on the aromatic ring at the meta position followed by the para position enhances the binding affinities of the ligand and the protein. The hydrogen-bond interactions of all the compounds **4(a-o)** and reference standard olprinone, with Tyr 844, Ser 857, Asn 860, Ser 864, Leu 872, and His 873, of the phosphodiesterase 3B target are summarized in Table 6. The compounds **4(l)** (−9.58031) followed by **4(f)** (−9.34526), **4(k)** (−9.29270), and **4(g)** (−8.49026) exhibit the highest H-bond interaction among the series and reference drug.

**Table 5:** Docking Scores^a^ of Imidazo[1,2-*a*]pyridin-3-yl Derivatives **4(a-o)** Docked with Phosphodiesterase 3B Target Selected for Screening

compound name	MolDock score	rerank score (kJ/mol)	interaction energy (kJ/mol)	steric	HBond (kJ/mol)
**4(a)**	–108.136	–32.3135	–125.473	–118.475	–6.99816
**4(b)**	–111.562	–78.3614	–120.628	–116.517	–4.11087
**4(c)**	–119.362	–89.9521	–128.679	–124.015	–4.66410
**4(d)**	–116.080	–79.6175	–125.506	–120.981	–4.52482
**4(e)**	–111.031	–77.0171	–120.362	–116.213	–4.14992
**4(f)**	–118.320	–83.1092	–128.576	–119.230	**–9.34526**
**4(g)**	**–130.663**	**–98.3234**	**–141.452**	**–132.962**	**–8.49026**
**4(h)**	–109.303	–73.0475	–118.408	–114.611	–3.79774
**4(i)**	–115.557	–85.9591	–123.242	–119.762	–3.48020
**4(j)**	–118.900	–87.8734	–126.795	–120.784	–6.01043
**4(k)**	–128.312	–81.8875	–132.019	–122.726	**–9.29270**
**4(l)**	–116.616	–65.0245	–127.611	–118.031	**–9.58031**
**4(m)**	–115.537	–85.9622	–123.216	–119.743	–3.47298
**4(n)**	–126.841	–94.7604	–130.499	–122.702	–7.79698
**4(o)**	–125.361	–94.0195	–131.192	–126.334	–8.55344
olprinone	–105.404	–82.9526	–116.144	–112.107	–4.03755

a MolDock score, rerank score, protein–ligand interaction, H-bond, and steric score.

**Table 6:** Molecular Interactions Analyses of Imidazo[1,2-*a*]pyridin-3-yl Derivatives **4(a-o)** and Standard Drug with Phosphodiesterase 3B Target

compound name	interaction	bond energy(kJ/mol)	bond length (Å)
**4(a)**	Ser 857 (O)–N (12)	–2.50000	3.04544
	Asn 860 (O)–O (24)	–0.12746	3.57007
	Asn 860 (N)–O (24)	–1.87070	2.82732
	Ser 864 (O)–O (24)	–2.50000	2.79733
**4(b)**	Ser 857 (O)–N (12)	–2.50000	2.79195
	Asn 860 (N)–O (23)	–0.84588	2.79866
	His 873 (N)–O (24)	–0.76499	3.44700
**4(c)**	Ser 857 (O)–N (12)	–2.16410	3.16718
	Leu 872 (O)–O (25)	–2.50000	2.73473
**4(d)**	Ser 857 (O)–N (12)	–2.50000	2.80191
	Asn 860 (N)–O (23)	–0.84081	2.71526
	His 873 (N)–O (24)	–1.18401	3.36320
**4(e)**	Ser 857 (O)–N (12)	–2.50000	2.80803
	Asn 860 (N)–O (23)	–0.85524	2.76409
	His 873 (N)–O (24)	–0.79468	3.44106
**4(f)**	Ser 857 (O)–N (12)	–2.50000	3.04756
	Ser 864 (O)–N (25)	–1.84526	3.23095
	Ser 864 (O)–O (27)	–2.50000	2.60137
	His 873 (N)–O (24)	–2.50000	2.92871
**4(g)**	Ser 857 (O)–N (12)	–2.40937	3.11813
	Ser 864 (O)–N (25)	–2.42875	3.11425
	Asn 860 (N)–O (26)	–1.15490	3.33927
	Ser 864 (O)–O (27)	–2.49723	2.59967
**4(h)**	Ser 857 (O)–N (12)	–2.50000	2.86090
	Asn 860 (N)–O (23)	–0.87540	2.75165
	His 873 (N)–O (24)	–0.42234	3.51553
**4(i)**	Ser 857 (O)–N (12)	–2.49607	3.10079
	Asn 860 (N)–O (23)	–0.98413	2.66454
**4(j)**	Ser 857 (O)–N (12)	–2.50000	3.07232
	Asn 860 (N)–O (23)	–1.01043	2.65725
	Leu 872 (O)–O (26)	–2.50000	2.75209
**4(k)**	Ser 857 (O)–N (12)	–2.50000	2.64745
	Asn 860 (N)–O (23)	–0.69619	2.81216
	Ser 864 (O)–N (26)	–2.50000	2.71435
	Asn 860 (N)–O (28)	–2.48162	3.10368
	Ser 864 (O)–O (28)	–0.03093	2.31362
	His 873 (N)–O (24)	–1.08396	3.38321
**4(l)**	Ser 857 (O)–N (12)	–2.50000	2.89671
	Asn 860 (N)–O (23)	–0.86219	2.61278
	Ser 857 (O)–O (26)	–1.98567	3.20287
	Ser 857 (O)–O (26)	–2.50000	2.61160
	His 873 (N)–O (24)	–0.91767	3.41647
	His 873 (N)–O (26)	–0.81479	3.43704
**4(m)**	Ser 857 (O)–N (12)	–2.50000	3.09672
	Asn 860 (N)–O (23)	–0.97299	2.64190
**4(n)**	Asn 860 (N)–N (11)	–0.02900	3.52202
	Ser 857 (O)–N (12)	–2.50000	3.01701
	Asn 860 (N)–O (24)	–0.27708	2.92932
	Tyr 844 (O)–O (26)	–2.49089	2.59891
	His 853 (N)–O (26)	–2.50000	3.06206
**4(o)**	Asn 860 (N)–N (11)	–0.08751	3.51567
	Ser 857 (O)–N (12)	–1.47668	3.30466
	Asn 860 (N)–O (24)	–0.14193	2.46943
	Tyr 844 (O)–N (26)	–2.50000	2.60095
	His 853 (N)–N (26)	–2.50000	3.02081
	Tyr 844 (O)–O (27)	–1.84732	2.08756
olprinone	Ser 857 (O)–N (8)	–1.53755	3.29249
	Ser 864 (O)–O (15)	–2.50000	3.08748

The screening of the imidazo[1,2-*a*]pyridin-3-yl derivatives against the target CXCR4 revealed that all the compounds exhibited inferior docking scores (MolDock score, rerank score, protein–ligand energy, and steric score) when compared with the reference standard GSK812397. The H-bond interactions with bond length and energies are depicted in Table 2S of Supporting Information. The compound **4(o)** followed by GSK812397 demonstrates high H-bond interactions (−9.5484 and −8.9460, respectively) among the series. Tyr 45, His 113, Thr 117, Cys 186, Arg 188, Gln 200, His 203, Tyr 255, Tyr 256, and Glu 288 of the CXCR4 target exhibit H-bond interactions with all of the compounds and GSK812397 target. The secondary structures for CXCR4 and GABAa are depicted in Figures 6 and 8, respectively.

For GABAa agonistic activity, compound **4(k)** showed the highest MolDock score and protein–ligand energy (−127.861 and −132.945, respectively) followed by **4(g)** (−127.803 and −132.640, respectively), while reference standard Necopidem exhibited the highest rerank and steric scores (−87.3621 and −126.952, respectively) followed by compound **4(g)** (−78.484 and −125.020, respectively). The hydrogen-bond interactions, bond length, and bond energy of compounds and standard drugs alpidem, necopidem, saripidem, and zolpidem are summarized in Table 4S of Supporting Information. The compounds **4(g)** followed by **4(k)** exhibit the highest H-bond interactions (−11.6198 and −9.79576, respectively) among the series and as compared to reference drugs. All of the compounds and reference drugs show H-bond interactions with the Asp 48, Val 50, Ser 51, Glu 52, Gln 185, Arg 216, Asp 245, Ser 247, Ala 248, Ala 249, Lys 274, Tyr 299, and Asn 303 of the GABAa agonist target.

**Figure 5:**
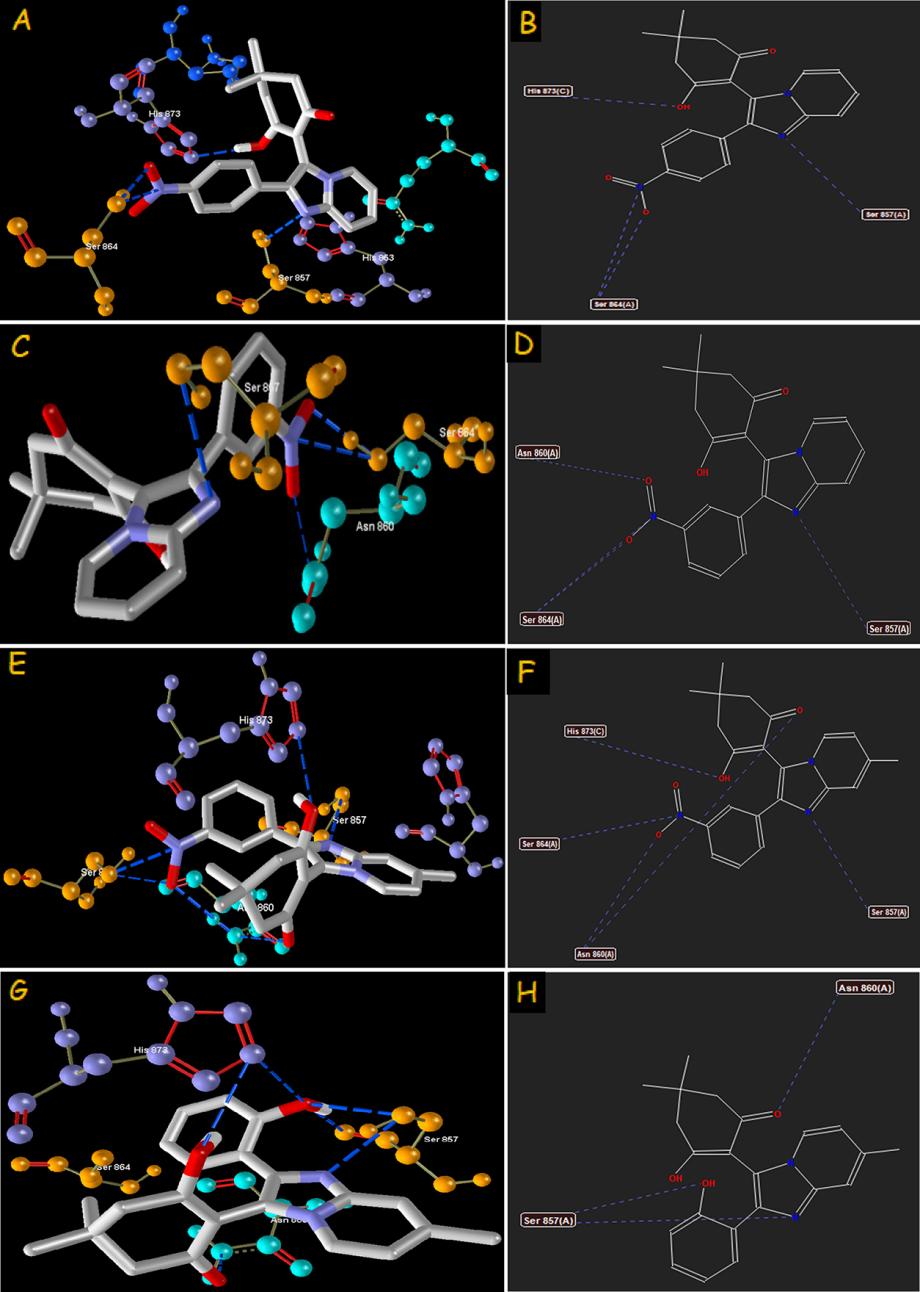
Hydrogen-bond interaction of compounds **4(f)** (A–B), **4(g)** (C–D), **4(k)** (E–F), and **4(l)** (G–H) with the phosphodiesterase 3B target.

However, the data need further experimental support to establish the selectivity profile of the imidazo[1,2-*a*]pyridin-3-yl derivatives. The binding interactions (H-bond and steric) of compound **4(k)** with farnesyl diphosphate synthase and **4(g)** with phosphodiesterase 3B are portrayed in Figures 3 and 5, respectively. The H-bond interactions of CXCR4 and GABAa with amino acids are illustrated in Figures 7 and 9, respectively. It can be postulated from the overall screening data (Tables 3 and 5) that compounds **4(k)** and **4(g)** exhibited the highest selectivity with farnesyl diphosphate synthase and phosphodiesterase 3B, respectively, among the series in terms of the MolDock score, and the other imidazo[1,2-*a*]pyridin-3-yl derivatives demonstrated comparable binding affinity against farnesyl diphosphate synthase and phosphodiesterase 3B in terms of overall docking scores (MolDock score, rerank score, protein–ligand energy, and steric score).

**Figure 6:**
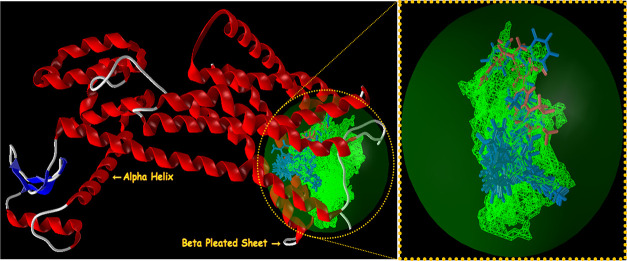
Secondary structure of CXCR4 binds with imidazo[1,2-*a*]pyridin-3-yl derivatives **4(a-o)** (blue) and standard drug GSK812397 (pink) inside the cavity (green framework).

**Figure 7:**
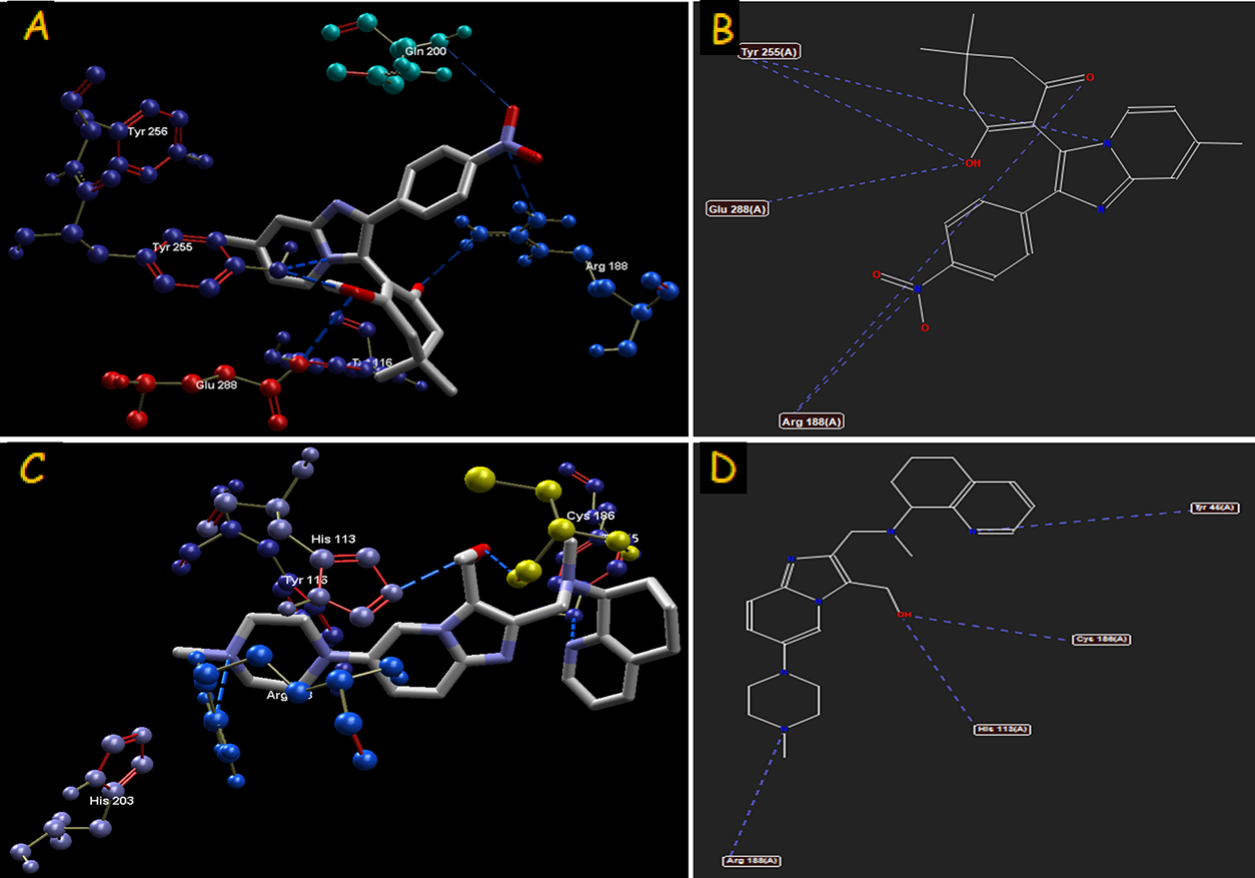
Hydrogen-bond interaction of compounds **4(o)** (A–B) and standard drug GSK812397 (C–D) with the CXCR4 target.

**Figure 8:**
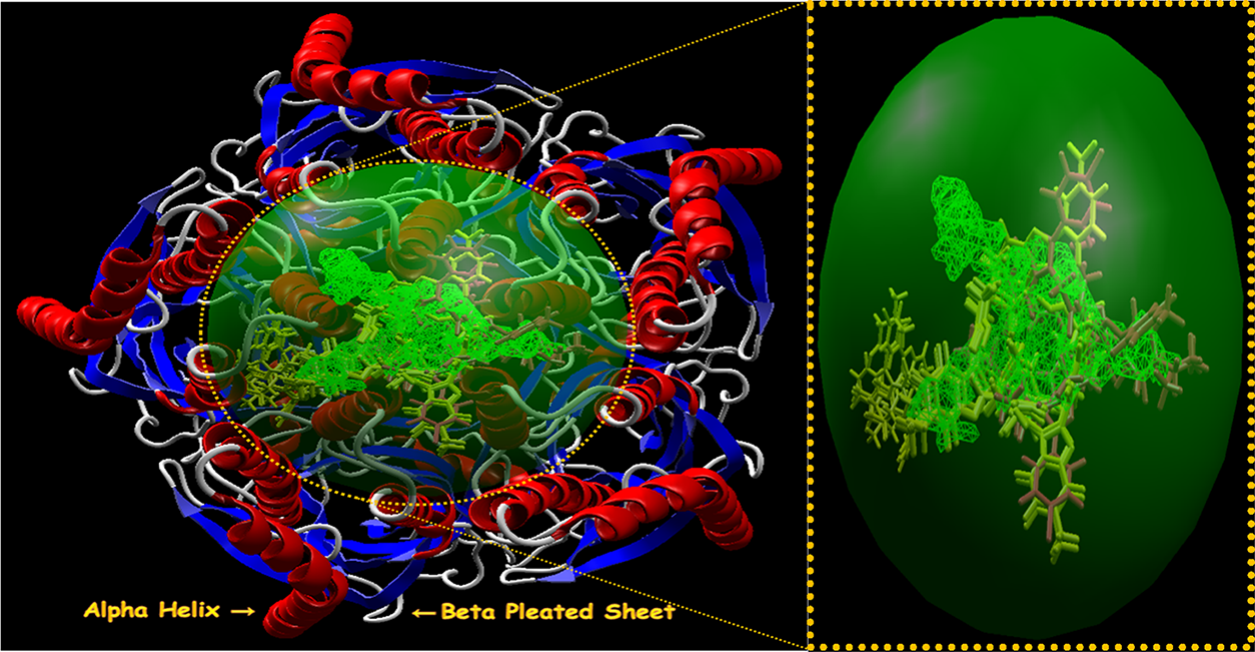
Secondary structure of GABAa agonist binds with imidazo[1,2-*a*]pyridin-3-yl derivatives **4(a-o)** (yellow) and standard drugs (pink) inside the cavity (green framework).

**Figure 9:**
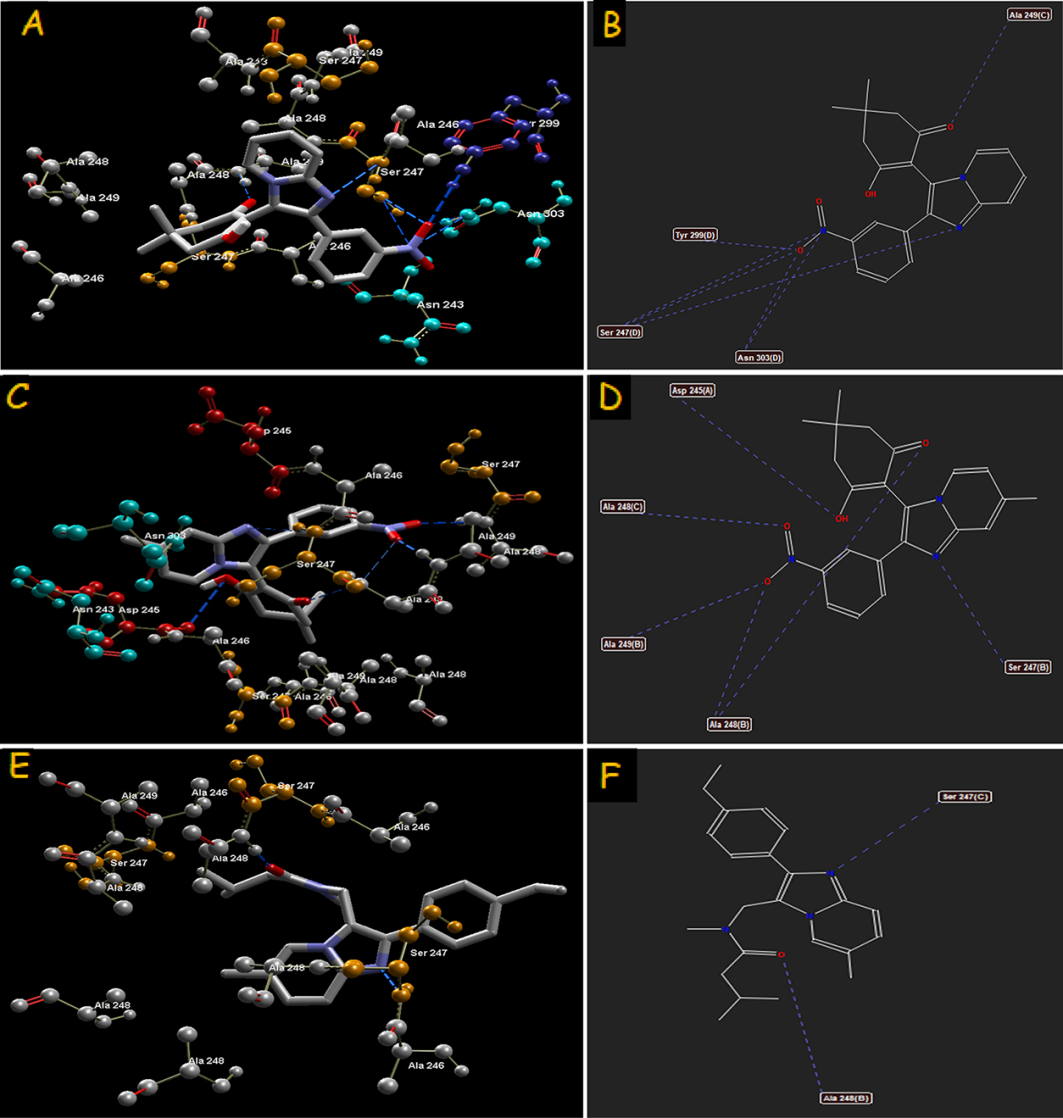
Hydrogen-bond interaction of compounds **4(g)** (A–B), **4(k)** (C–D), and standard drug necopidem (E–F) with the GABAa target.

### 3.3 Prediction of ADMET, Toxicity, and Drug-Likeness Properties

**Figure 10:**
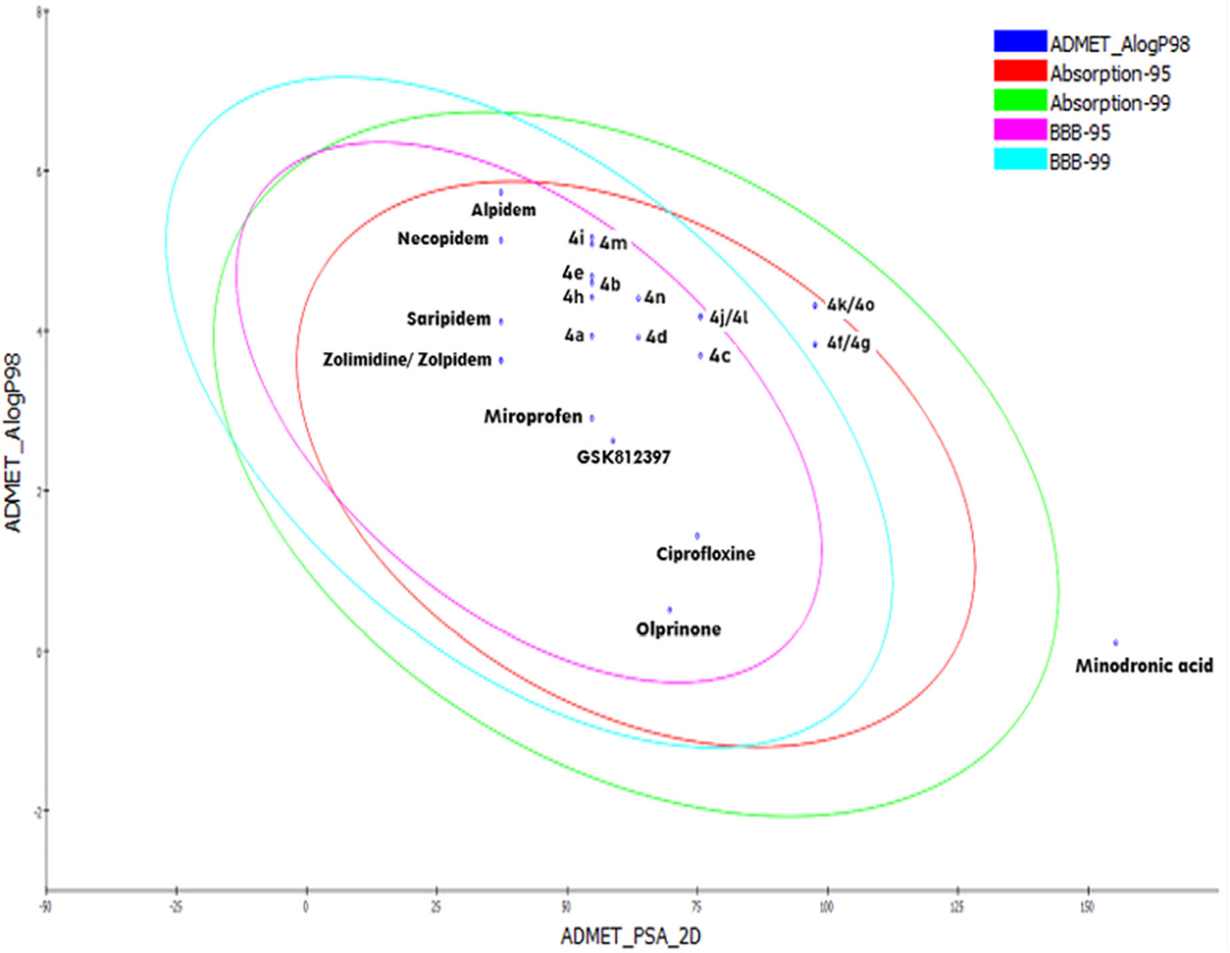
A plot of AlogP98 versus 2D polar surface area (PSA) for the synthesized compounds **4(a-o)** and standard drugs.

The pharmacokinetic profiles of all of the synthesized compounds **4(a-o)** and standard drugs under investigation were envisioned by ADMET (absorption, distribution, metabolism, excretion, and toxicity) models provided by the Discovery Studio 2019 program.^32^ The biplot exhibits the two analogous 95% and 99% confidence ellipses corresponding to the human intestinal absorption (HIA) and the blood–brain barrier (BBB) models, respectively, as depicted in Figure 10. The plot presents green and blue eclipses with 99% confidence limits, whereas the red and pink eclipses show 95% confidence limits for the intestinal absorption (HIA) and blood–brain barrier (BBB), respectively.^33,43,44^ The screened results of ADMET comprised of some descriptors such as the blood–brain barrier (BBB), absorption, solubility, hepatotoxicity, cytochrome P_450_ 2D6 (CYP2D6), plasma protein binding (PPB), AlogP98, and PSA2D are summarized in Table 7.

**Table 7:** Absorption, Distribution, Metabolism, Excretion, and Toxicity (ADMET) Predictions for the Synthesized Compounds **4(a-o)** and Standard Drugs

compounds	BBB level^a^	absorption level^b^	hepatotoxicity^c^	CYP2D6^d^	PPB^e^	solubility^f^	AlogP98^g^	PSA 2D
**4a**	1	0	0	0	true	–5.186	3.932	54.725
**4b**	1	0	0	1	true	–5.896	4.596	54.725
**4c**	2	0	0	0	false	–4.686	3.690	75.541
**4d**	1	0	0	0	true	–5.199	3.915	63.655
**4e**	1	0	0	0	true	–5.972	4.680	54.725
**4f**	4	0	0	0	true	–5.326	3.826	97.548
**4g**	4	0	0	0	true	–5.350	3.826	97.548
**4h**	1	0	0	1	true	–5.668	4.418	54.725
**4i**	1	0	0	0	true	–6.449	5.166	54.725
**4j**	2	0	0	0	true	–5.162	4.176	75.541
**4k**	4	0	0	0	true	–5.818	4.312	97.548
**4l**	2	0	0	0	true	–5.141	4.176	75.541
**4m**	1	0	0	1	true	–6.374	5.082	54.725
**4n**	1	0	0	0	true	–5.671	4.402	63.655
**4o**	4	0	0	0	true	–5.793	4.312	97.548
Alpidem	0	0	0	0	true	–6.455	5.729	37.262
Ciprofloxacin	3	0	0	0	false	–3.162	1.435	74.932
GSK812397	2	0	0	0	true	–3.910	2.621	58.743
minodronic acid	4	3	0	0	false	–1.462	0.098	155.288
miroprofen	2	0	0	0	true	–3.750	2.905	54.725
necopidem	0	0	0	0	true	–6.072	5.130	37.262
olprinone	3	0	0	0	false	–2.255	0.509	69.655
saripidem	1	0	0	0	true	–5.174	4.114	37.262
zolimidine	2	0	0	0	true	–3.812	2.304	51.210
zolpidem	1	0	0	0	true	–4.984	3.628	37.262

a 0, 1, 2, 3, and 4 denote very high, high, medium, low, and undefined, respectively.

b 0, 1, 2, and 3 denote good absorption, moderate absorption, low absorption, and very low absorption, respectively.

c 0 and 1 represent nontoxic and toxic, respectively.

d 0 and 1 denote noninhibitor and inhibitor, respectively.

e True symbolizes binding, and false symbolizes nonbinding of the drug.

f –6.0 to −4.0, −4.0 to −2.0, and −2.0 to 0.0 represents low, good, and optimal solubility, respectively.

g AlogP98 > 5 indicates good absorption through BBB.

According to the ADMET prediction, four of the compounds **4(f-g)**, **4(k)**, and **4(o)** were outside the 99% BBB confidence ellipse, meaning that the quality of the results obtained were unknowable (undefined level of 4). All of the compounds **4(a-o)** and standard drugs except minodronic acid were inside the 99% absorption ellipse that revealed the good intestinal absorption of the compounds. The obtained AlogP98 values of all the compounds, except **4(i)** and **4(m)**, were found to be less than five, which divulges the easy absorption of the drug through the blood–brain barrier.^44^ The two standard drugs alpidem, and necopidem indicate the higher value of atom-based Log P98 among the standard drugs and all of the synthesized compounds **4(a-o)**.

The cytochrome P450 2D6 (CYP2D6) is intricate in the metabolism of a varied range of xenobiotics, and its inhibition by a drug may lead to serious drug–drug interactions. Consequently, determining the CYP2D6 inhibition is a vital part of the drug discovery and development process.^43^ All of the compounds, except **4(b)**, **4(h)**, and **4(m)**, were classified as noninhibitors of CYP2D6. The hepatotoxicity model predicts the occurrence of dose-dependent human toxicity. According to the hepatotoxicity model, all of the compounds and standard drugs were classified as non-hepatotoxic. All of the synthesized compounds show low solubility, as compared to the standard drugs assorted from lower to optimal solubility. The pharmaceutical activity is determined by the free drug concentration; therefore, the possible plasma protein binding of compounds must be considered.^43^ All of the synthesized compounds were likely to be binding, whereas only **4(c)** was likely to be nonbinding among the synthesized library of compounds.

The toxicity predictions of the synthesized compounds **4(a-o)** were also investigated with Discovery Studio 2019 using the toxicity prediction by a komputer-assisted technology (TOPKAT) protocol.^32,33^ The acquired outcomes of the TOPKAT protocol are summarized in Table 8. The predicted toxicity values are in the range of 0.0–0.30, 0.30–0.70, and 0.70–1.0 representing the nontoxic, intervocal, and toxic nature of the drugs, respectively.^33^ The resultant toxicity parameters revealed the potency of all the synthesized compounds, except **4(h)** and **4(m)** with less toxicity and a greater safety index. The toxicity predictions of all of the compounds were endowed to be preferable for the development of this synthesized library of compounds into medicinal drugs.

**Table 8:** Toxicity Prediction of All the Synthesized Compounds **4(a-o)**

compounds	rat male NTP^a^ prediction	mouse male NTP^a^ prediction	Ames mutagenicity prediction	skin irritation	aerobic biodegradability prediction
**4a**	non-carcinogen (0.000)	non-carcinogen (0.000)	non-mutagen (0.000)	non-irritant (0.000)	non-biodegradable (0.000)
**4b**	non-carcinogen (0.000)	non-carcinogen (0.000)	non-mutagen (0.000)	non-irritant (0.000)	non-biodegradable (0.000)
**4c**	non-carcinogen (0.010)	non-carcinogen (0.000)	non-mutagen (0.000)	non-irritant (0.000)	non-biodegradable (0.000)
**4d**	non-carcinogen (0.000)	non-carcinogen (0.000)	non-mutagen (0.000)	non-irritant (0.000)	non-biodegradable (0.000)
***4e***	non-carcinogen (0.001)	non-carcinogen (0.000)	non-mutagen (0.094)	non-irritant (0.000)	non-biodegradable (0.000)
***4f***	non-carcinogen (0.001)	non-carcinogen (0.000)	non-mutagen (0.022)	non-irritant (0.000)	non-biodegradable (0.000)
**4g**	non-carcinogen (0.000)	non-carcinogen (0.000)	non-mutagen (0.000)	non-irritant (0.003)	non-biodegradable (0.131)
**4h**	non-carcinogen (0.000)	non-carcinogen (0.000)	non-mutagen (0.000)	non-irritant (0.000)	biodegradable (1.000)
***4i***	non-carcinogen (0.000)	non-carcinogen (0.000)	non-mutagen (0.000)	non-irritant (0.002)	non-biodegradable (0.000)
**4j**	non-carcinogen (0.001)	non-carcinogen (0.000)	non-mutagen (0.002)	non-irritant (0.000)	non-biodegradable (0.000)
**4k**	non-carcinogen (0.000)	non-carcinogen (0.000)	non-mutagen (0.000)	non-irritant (0.030)	non-biodegradable (0.000)
**4l**	non-carcinogen (0.000)	non-carcinogen (0.000)	non-mutagen (0.000)	non-irritant (0.000)	non-biodegradable (0.000)
***4m***	non-carcinogen (0.009)	non-carcinogen (0.000)	non-mutagen (0.000)	irritant (0.992)	non-biodegradable (0.000)
**4n**	non-carcinogen (0.001)	non-carcinogen (0.000)	non-mutagen (0.008)	non-irritant (0.000)	non-biodegradable (0.002)
**4o**	non-carcinogen (0.000)	non-carcinogen (0.000)	non-mutagen (0.000)	non-irritant (0.000)	non-biodegradable (0.016)

a NTP: National Toxicology Program.

To determine the ability of the drug to diffuse passively through the BBB, analyses of drug-likeness were performed by Lipinski’s rule-of-five prediction of the drug. The rule states that a compound can be considered biologically active for an oral administration in humans if it does not violate more than one of these thresholds: the molecular weight (MW) of the molecule must be <500 Da, octanol/water partition coefficient (iLOGP) must be ≤5, number of hydrogen-bond acceptors (nHBA) must be ≤10, number of hydrogen-bond donors (nHBD) ≤ 5, and topological polar surface area (TPSA) ≤ 40 Å^2^.^45^ To ascertain the drug-likeness character of compounds, the Discovery Studio 2019 program was used for determining the substantial pharmacokinetic properties.^32,33^ The outputs of drug-likeness properties of synthesized compounds **4(a-o)** in comparison with standard drugs are summarized in Table 9. The results reveal that the synthesized imidazo[1,2-*a*]pyridine derivatives **4(a-o)** have zero violations of Lipinski’s rule.

**Table 9:** Physicochemical Properties of All of the Synthesized Compounds **4(a-o)** and Standard Drugs on the Basis of Lipinski’s Rule of Five^a^

compounds	MW(g/mol)	nHBA	nHBD	TPSA (Å^2^)	Log P_o/w_	nLV
**4a**	332.40	3	1	54.60	3.56	0
**4b**	366.84	3	1	54.60	4.12	0
**4c**	348.40	4	2	74.83	3.18	0
**4d**	362.42	4	1	63.83	3.59	0
**4e**	411.29	3	1	54.60	4.19	0
**4f**	377.39	5	1	100.42	2.85	0
**4g**	377.39	5	1	100.42	2.84	0
**4h**	346.42	3	1	54.60	3.89	0
**4i**	425.32	3	1	54.60	4.54	0
**4j**	362.42	4	2	74.83	3.49	0
**4k**	391.42	5	1	100.42	3.20	0
**4l**	362.42	4	2	74.83	3.45	0
**4m**	380.87	3	1	54.60	4.45	0
**4n**	376.45	4	1	63.83	3.91	0
**4o**	391.42	5	1	100.42	3.21	0
alpidem	404.33	2	0	37.61	4.89	0
ciprofloxacin	331.34	5	2	74.57	1.10	0
GSK812397	402.35	7	1	60.14	2.44	0
minodronic acid	680.79	8	5	172.21	–1.74	0
miroprofen	266.29	3	1	54.60	2.64	0
necopidem	363.50	2	0	37.61	4.40	0
olprinone	250.26	3	1	73.95	1.67	0
saripidem	341.83	2	0	37.61	3.66	0
zolimidine	272.32	3	0	59.82	2.25	0
zolpidem	307.39	2	0	37.61	3.13	0

a MW molecular weight, nHBD number of hydrogen-bond donor, nHBA number of hydrogen-bond acceptor, TPSA topological polar surface area, Log Po/w octanol/water partition coefficient, nLV number of Lipinski violation.

### 3.4 Frontier Molecular Orbital Analysis

The frontier orbital of the chemical compounds is a very significant parameter in drug design and in recognizing their reactivity.^46−48^ The higher value of the highest occupied molecular orbital (HOMO) of a molecule can donate electrons to suitable acceptor molecules with low energy and empty molecular orbitals (LUMO). The predicted frontier orbital energies, the chemical potential (μ),^36^ chemical hardness (η),^49^ and electrophilicity index (ω)^37,50^ are summarized in Table 10.

**Table 10:** Electron Density-Based Molecular Properties Calculated with the DFT/B3LYP/6-311G + + (d,p) Level of Theory for Imidazo[1,2-*a*]pyridin-3-yl Derivatives **4(a-o)**^a^

compounds	*E*_HOMO_(eV)	*E*_LUMO_(eV)	Δ*E* = *E*_(LUMO–HOMO)_ (eV)	η (eV)	μ (eV)	ω (eV)
**4(a)**	–5.3878	−1.4150	3.9728	1.9864	–3.4014	2.9122
**4(b)**	–5.5511	–1.5238	4.0273	2.0137	–3.5375	3.1072
**4(c)**	–5.2790	–1.3878	3.8912	1.9456	–3.3334	2.8556
**4(d)**	–5.4151	–1.6055	3.8096	1.9048	–3.5103	3.2345
**4(e)**	–5.5239	–1.5238	4.0001	2.0001	–3.5239	3.1043
**4(f)**	–5.8232	–2.4218	3.4014	1.7007	–4.1225	4.9965
**4(g)**	–5.7144	–2.2858	3.4286	1.7143	–4.0001	4.6669
**4(h)**	–5.2790	–1.3606	3.9184	1.9592	–3.3198	2.8126
**4(i)**	–5.4151	–1.4966	3.9185	1.9593	–3.4559	3.0478
**4(j)**	–5.1702	–1.3606	3.8096	1.9048	–3.2654	2.7989
**4(k)**	–5.6055	–2.2585	**3.3470**	1.6735	–3.9320	4.6192
**4(l)**	–5.1702	–1.2517	3.9185	1.9593	–3.2110	2.6312
**4(m)**	–5.4151	–1.4966	3.9185	1.9593	–3.4559	3.0478
**4(n)**	–5.1429	–1.3334	3.8095	1.9048	–3.2382	2.7525
**4(o)**	–5.6872	–2.3674	**3.3198**	1.6599	–4.0273	4.8856

a (η) Chemical hardness, (ω) electrophilicity index of molecules, and (μ) electronic chemical potential.

All the molecular structures of imidazo[1,2-*a*]pyridin-3-yl derivatives **4(a-o)** were optimized utilizing the level of theory as mentioned in the Experimental Section.^34^ After optimization of all the structures, the vibrational modes were checked to be true energy minima by frequency analyses, and it revealed that no imaginary frequencies were observed. The chemical hardness (η) shows the reactivity of the molecule, where a larger η value indicates a less reactive nature than a molecule having a smaller value of η. A hard molecule that possesses a large HOMO–LUMO gap means high excitation energies are required to manifold excited states and be less reactive, and their electron density is less easily changed than a soft molecule.^51^

**Figure 11:**
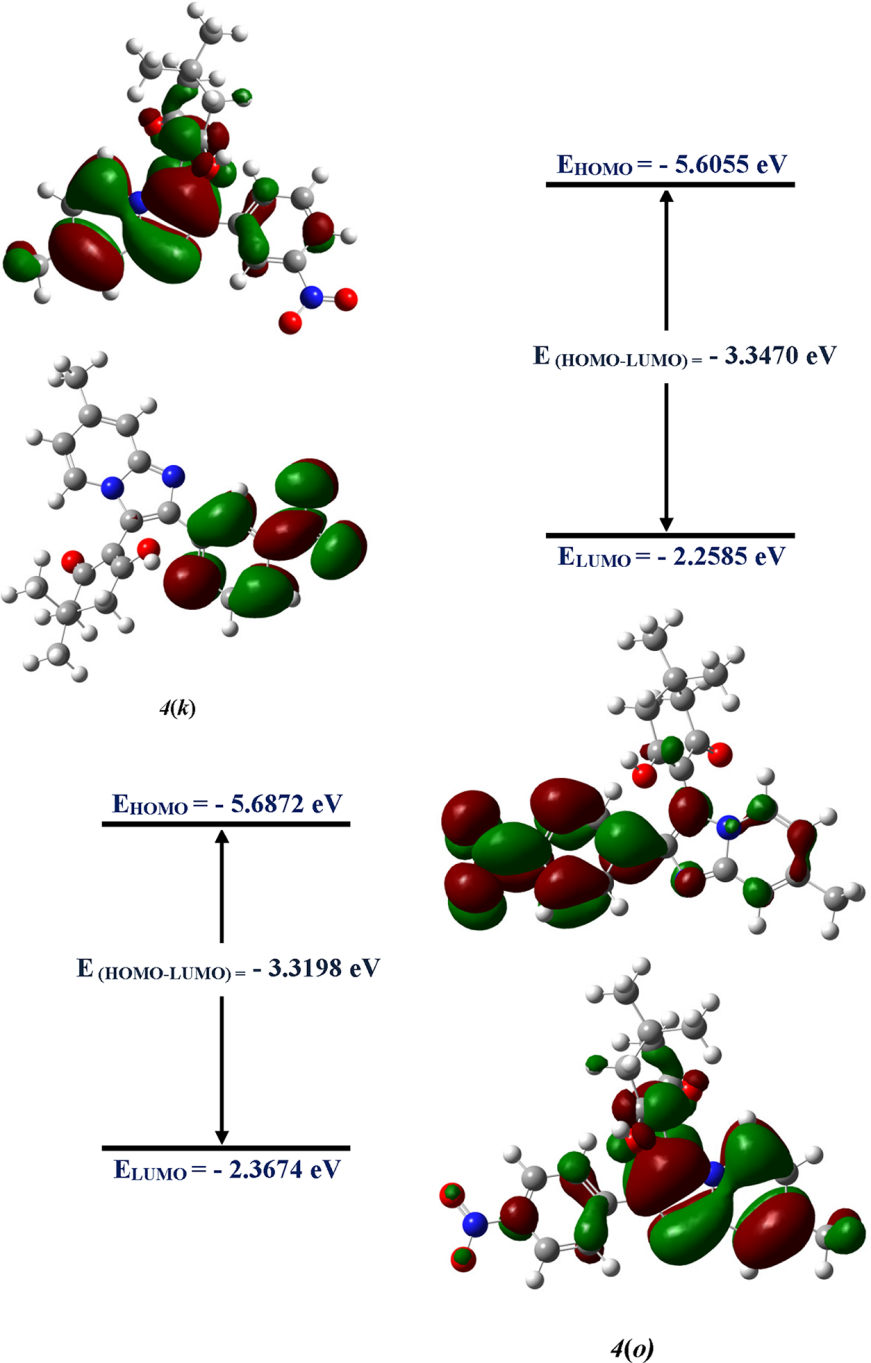
Optimized geometries with a frontier molecular orbital of **4(k)** and **4(o)**.

The FMOs distribution patterns at the ground state of imidazo amalgamated pyridine hybrid molecules **4(k)** and **4(o)** are depicted in Figure 11. All the optimized geometries with a frontier molecular orbital of **4(a-j)** and **4(l-n)** are portrayed in Figure 2S of the Supporting Information. The trend of the hardness of molecules in the following increasing order:
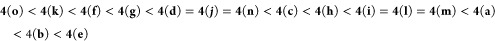
The trend of chemical hardness reveals that compound **4(e)** is the least reactive, while the compounds **4(o)**, **4(k)**, **4(f)**, and **4(g)**, respectively, are the most reactive molecules among the series of imidazo-pyridine derivatives. The electrophilicity index (ω) divulges the stabilization energy when the system is augmented by an electronic charge from the surrounding environment.^50^ Furthermore, the calculated results also indicate that the imidazo-pyridine hybrid molecules substituted with a nitro functional group on the aryl are more reactive and hence are more active as highlighted in the *in silico* studies for compounds **4(o)**, **4(k)**, **4(f)**, and **4(g)** with the nitro group substitution found to be more potent against the farnesyl diphosphate synthase, human phosphodiesterase 3B, CXCR4, and GABAa agonist targets. Thus, the result obtained from the DFT studies by the level of theory used is qualitatively only and in good agreement with the outcomes of *in silico* analyses.

### 3.5 Local Reactivity Descriptors and Electrostatic Potential Surface Analysis

**Figure 12:**
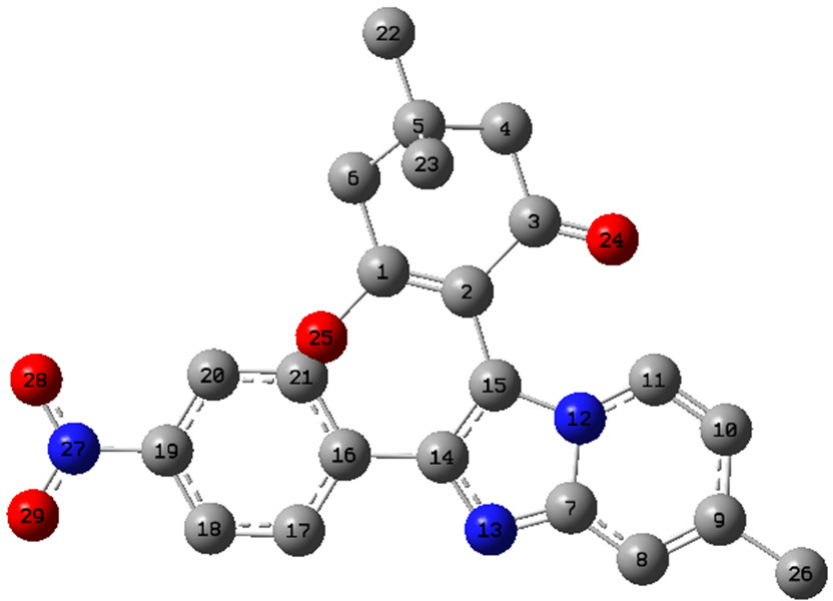
Labeled image of atoms for compound **4(o)**.

To predict the reactivity and selectivity, the local reactivity descriptors such as Fukui functions (*f*_*k*_^*+*^, *f*_*k*_^*-*^, and *f*_*k*_^0^) were calculated at the DFT/B3LYP/6-311G++ (d, p) level of theory for the synthesized compound **4(o)** using the natural bond order (NBO) analysis method.^39^ The labeled image of the atoms for **4(o)** is depicted in Figure 12.

The charges extant on each atom were calculated as *qk* (*k*th atom) for the cationic (*N* – 1), anionic (*N* + 1), and neutral (*N*) molecule of the compound **4(o)**. The atomic sites likely to undergo electrophilic and nucleophilic attacks can be ascertained using the highest value of the Fukui function for the HOMO (*f*_*k*_^*-*^) and LUMO (*f*_*k*_^*+*^), respectively.^40^ The local reactivity descriptors for compound **4(o)** in terms of Fukui functions are summarized in Table 11. The highest value of the Fukui function for the LUMO (*f*_*k*_^*+*^) is 1.4858 followed by 0.5879 present around the atoms O_25_ and C_5_, which indicates the most electrophilic sites, respectively, and these are the probable sites for nucleophilic attacks. However, the atom C_21_ characterized by the highest value of the Fukui function for the HOMO (*f*_*k*_^*-*^) is 0.7955, which represents the nucleophilic site and hence possible site for the electrophilic attack. The highest value of the Fukui function for the (*f*_*k*_^*0*^) is 0.0903 present around atom C_5_, which reveals the most significant site for the radical attacks.

**Table 11:** Local Reactivity Descriptors for Compound **4(o)** in Terms of Fukui Function Using DFT/B3LYP/6-311G + + (d, p) Level of Theory

atom	*q*_*k*_ (N)	*q*_*k*_ (*N* + 1)	*f*_*k*_^+^	*f*_*k*_^-^	*f*_*k*_^0^
C_1_	–0.151310	–0.38542	–0.401360	0.250047	0.007970
C_2_	–0.092970	0.42033	0.249521	–0.342490	0.085405
C_3_	0.238183	–0.47370	–0.483440	0.721625	0.004871
C_4_	0.184695	–0.22889	–0.304330	0.489020	0.037720
C_5_	–0.362620	0.29922	**0.587872**	–0.950490	–0.144325
C_6_	0.174867	–0.12933	–0.309850	0.484712	**0.090258**
C_7_	0.312993	0.10023	0.122315	0.190678	–0.011042
C_8_	0.018006	0.23488	0.088316	–0.070310	0.073283
C_9_	–0.081770	0.11073	0.221697	–0.303470	–0.055486
C_10_	–0.054300	0.09544	–0.083020	0.028717	0.089229
C_11_	0.218577	–0.13781	–0.092950	0.311523	–0.022431
N_12_	–0.355590	0.29693	0.247717	–0.603310	0.024607
N_13_	–0.378090	0.08790	0.007784	–0.385870	0.040059
C_14_	0.061015	–0.355810	–0.393740	0.454753	0.018966
C_15_	0.104763	0.091094	0.097453	0.007310	–0.003180
C_16_	–0.163560	0.659550	0.587435	–0.750990	0.036058
C_17_	0.119294	–0.270240	–0.435790	0.555082	0.082774
C_18_	0.033791	0.474118	0.331978	–0.298190	0.071070
C_19_	0.210717	–0.463470	–0.462950	0.673667	–0.000261
C_20_	–0.217070	–0.798400	–0.934870	0.717799	0.068236
C_21_	0.364037	–0.545010	–0.431450	**0.795488**	–0.056778
C_22_	0.128279	0.214642	0.058900	0.069379	0.077871
C_23_	0.138569	0.009307	–0.145230	0.283802	0.077270
O_24_	–0.344970	–0.018290	–0.188560	–0.156420	0.085135
O_25_	0.201408	1.660380	**1.485765**	–1.284360	0.087308
C_**26**_	0.114567	0.167997	0.018904	0.095663	0.074547
N_27_	0.123946	–0.227000	–0.256400	0.380350	0.014704
O_28_	–0.229870	0.098736	–0.026610	–0.203250	0.062675
O_29_	–0.315590	0.011858	–0.155120	–0.160470	0.083489

**Figure 13:**
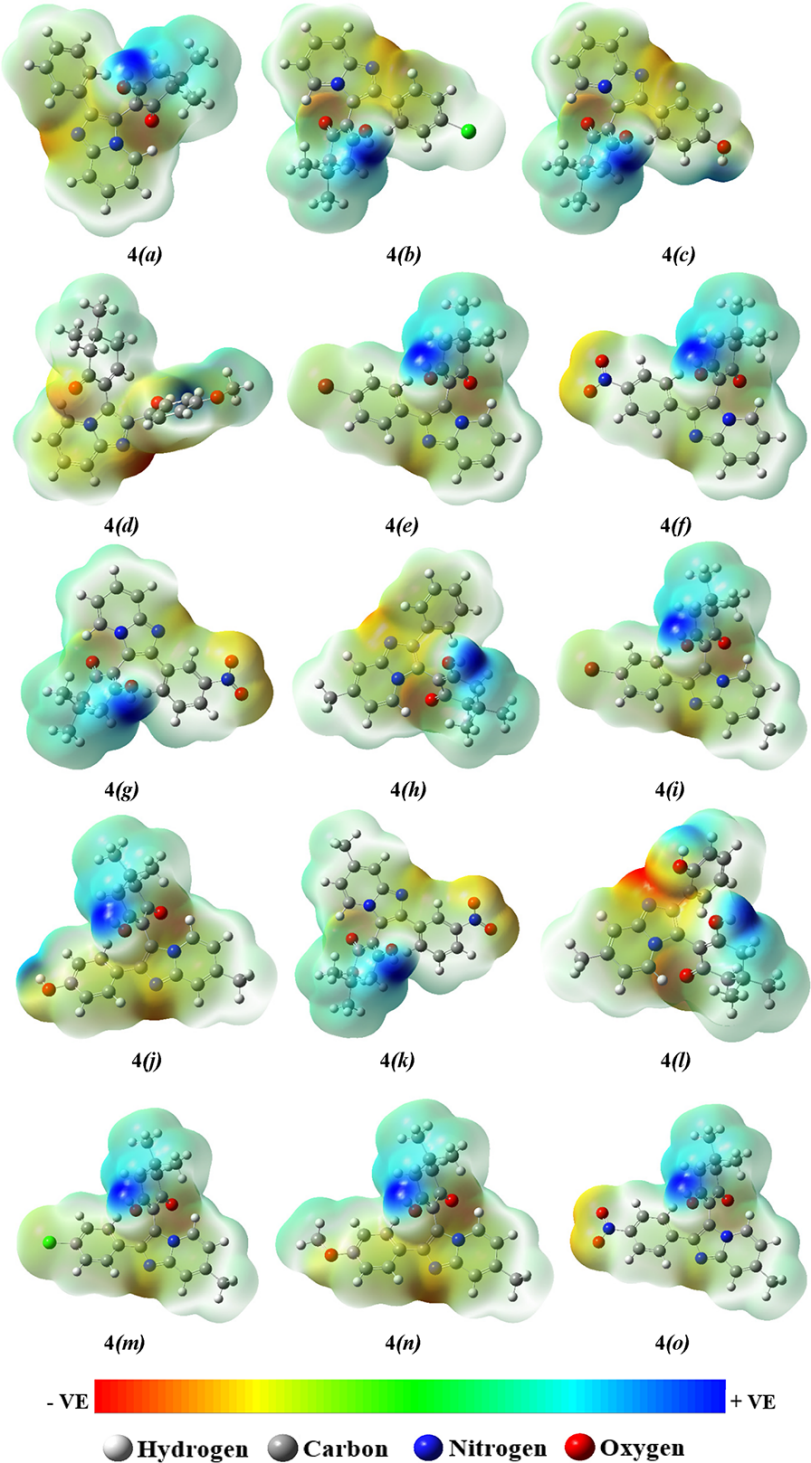
Molecular electrostatic potential (MEP) maps of the synthesized compounds **4(a-o)**.

The molecular electrostatic potential surface to envisage the reactive sites in the optimized structure of all of the synthesized compounds **4(a-o)** is mapped in Figure 13. The red color in the map signifies an electronegative region with minimum electrostatic potential that divulges its susceptibility to electrophilic attack. Similarly, the blue color represents an electropositive region liable for the nucleophilic attack, whereas green is a region of zero potential.^52,53^ The MEP diagrams revealed that the red region corresponds to the oxygen atom of the carbonyl group of dimedone, imine group of the imidazole ring, and oxygen atoms of substituted nitro, hydroxyl, and methoxy groups of the acetophenone ring, which indicates its most reactivity toward the intermolecular hydrogen-bonding interactions. The hydrogen atoms of the hydroxyl group of the dimedone ring exhibit the electropositive nature as represented in blue regions.

## 4 Conclusion

An ultrasonic-assisted efficient and environmentally sustainable methodology is presented for the synthesis of pharmacologically significant 2-phenylimidazo[1,2-*a*]pyridine-3yl scaffolds. The molecular-iodine-catalyzed protocol for the synthesis of biologically active synthetic equivalents has been envisaged to intensify the viability and yield of the products. The higher environmental compatibility and sustainability factors of this protocol thereby satisfy the triple bottom line philosophy of green and sustainable chemistry. The reaction protocol is also feasible for the multigram scale, which devises an economically affordable methodology on a large scale.

The virtual screening of synthetic moieties against several biological targets attributed significant interactions with the active site of receptor proteins. The compounds **4(k)** and **4(g)** have come to light as potential inhibitors with the highest selectivity against farnesyl diphosphate synthase and phosphodiesterase 3B, respectively. The acquired results indicate that the theoretical studies are in good agreement with the outcomes of *in silico* analyses. This screening study opens the way for *in vitro* and *in vivo* testing of synthesized derivatives as potent inhibitors with an improved pharmacological profile against farnesyl diphosphate synthase, phosphodiesterase III, CXCR4, and GABAa receptor agonists.

## Supporting Information Available

General experimental section, characterization data, FT-IR, ^1^H, and ^13^C NMR spectra for selected synthesized compound, secondary structures of biological targets, docking scores, optimized geometries of some structures (PDF)
