# Supplementary material for: Molecular Iodine-Catalyzed Synthesis of Imidazo[1,2-a]Pyridines: Screening of Their In Silico Selectivity, Binding Affinity to Biological Targets, and Density Functional Theory Studies Insight
Source: ACS Omega. 2022 Jun 22;7(26):22421–39. doi: 10.1021/acsomega.2c01570 (PMC9260945; doi:10.1021/acsomega.2c01570)
Supplement: Supplementary file 1 — ao2c01570_si_001.pdf [file ao2c01570_si_001.pdf]

Molecular Iodine-Catalyzed Synthesis of Imidazo[1,2-*a*]Pyridines: Screening of their *In Silico* Selectivity, Binding Affinity to Biological Targets, and Density Functional Theory Studies Insight

Deepika Geedkar, Ashok Kumar, and Pratibha Sharma*

School of Chemical Sciences, Devi Ahilya University, Indore 452001, Madhya Pradesh

Table of Contents

- **General Information**...S3
- **The secondary structure of all the selected targets with detected active sites**..................S4-S5
- **Docking scores (MolDock score, re-rank score, protein-ligand interaction, H-bond and steric score) of imidazo[1,2-a]pyridin-3-yl derivatives *4(a-o)* docked with different targets selected for screening**...S6-S9
- **The active sites (cavities) (1-5) detected in different selected targets along with their volume and surface area** ……………………………………………………………….………………S10
- **The Optimized geometries with a frontier molecular orbital of *4(a-j)* and 4*(l-n)*** ……S11-S17
- **Physical analyses data for imidazo[1,2-*a*]pyridin-3-yl derivatives *4(a-o)***……….……....S18-S32
- **Figure S1-S4. The spectrums of imidazo[1,2-*a*]pyridin-3-yl derivatives *4(a-o)***.............S33-S36
- **References**...S37

❖ General Information

All chemicals and solvents were purchased from Sigma Aldrich and Merck India. The reactions were performed in an aerobic atmosphere without any specific precautions. Melting points were determined in open capillary tubes on a Veego melting-point apparatus and are uncorrected. The ultrasound-promoted reactions were performed using a Cole Parmer-ultrasonic processor Model CPX 130, with a maximum power of 130 W, operating at amplitude of 60% and a frequency of 20 kHz. All the products were characterized by their spectroscopic data (FTIR, ^{1}H and ^{13}C NMR). The ^{1}H and ^{13}C NMR spectra of the synthesized compounds were recorded at 400 and 100 MHz respectively, using Bruker Advance II 400 NMR spectrometer in DMSO-d_6 solvent, and the chemical shifts (δ) were expressed in parts per million. Spin multiplicities are described as s (singlet), d (doublet), t (triplet), q (quartet), and m (multiplet). The mass spectra were attained on Water's Q-Tof Micromass instrument using electron spray ionization (ESI) in positive mode. Fourier Transform Infra Red (FTIR) spectra were recorded as ATR spectra within the range of 4000–400 cm^{-1} using Frontier Perkin-Elmer FTIR SP 10 STD. Thin-layer chromatography (TLC) is performed using precoated aluminum sheets with silica gel 60 F254.[1-3]

* ***Molecular docking studies***

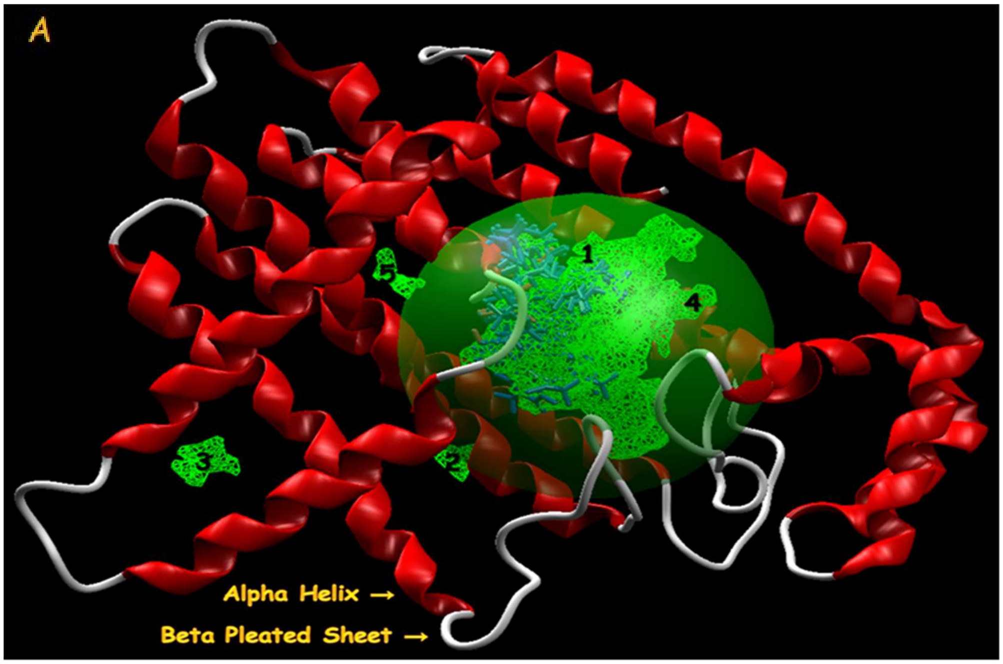

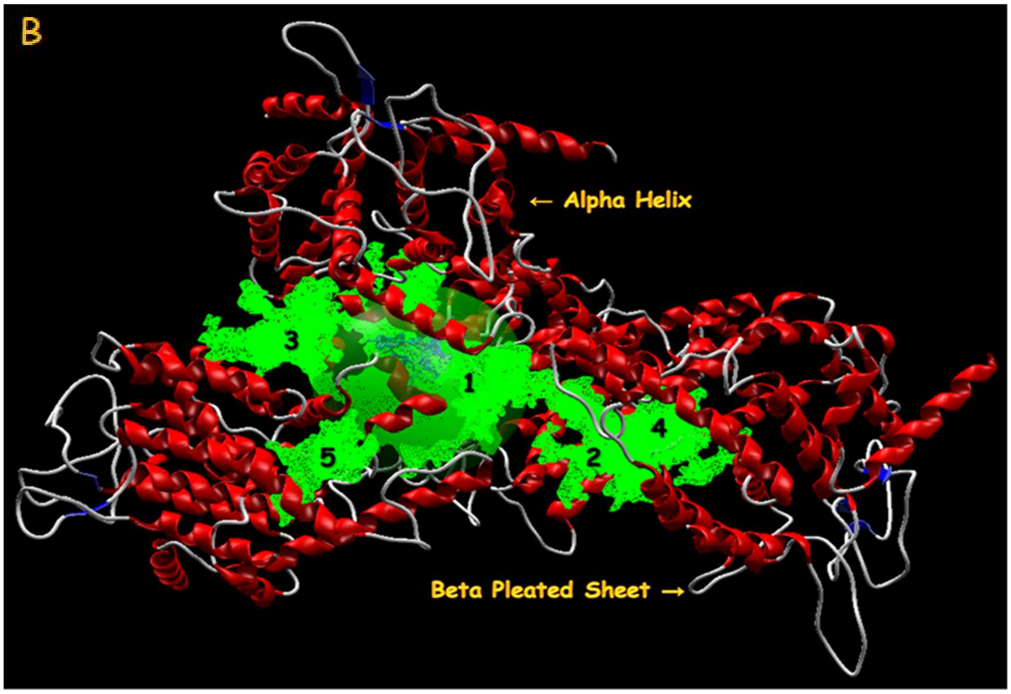

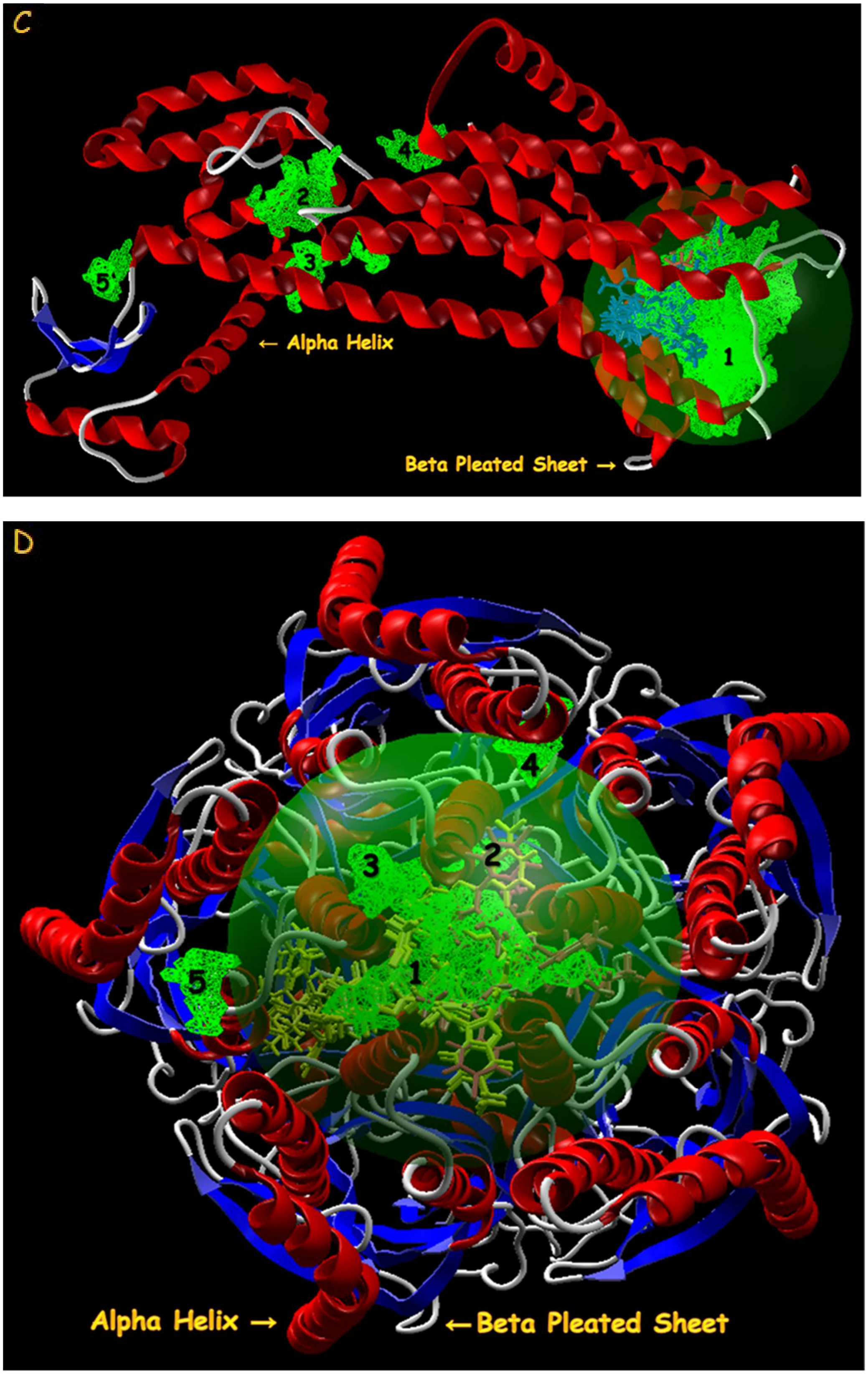

Figure S1. The Secondary structure of farnesyl diphosphate synthase (A), Human Phosphodiesterase 3B (B), CXCR4 (C), and GABAa (D) along with active sites (1-5) (green framework)

Table S1. Docking scores (MolDock score, re-rank score, protein-ligand interaction, H-bond and steric score) of imidazo[1,2-*a*]pyridin-3-yl derivatives ***4(a-o)*** docked with CXCR4 target selected for screening

Compound Name	MolDock Score	Rerank Score (kJ/mol)	Interaction Energy (kJ/mol)	Steric	HBond (kJ/mol)
4(a)	-110.947	-15.4683	-122.407	-119.910	-2.49659
4(b)	-116.564	-32.4437	-122.593	-121.055	-1.53804
4(c)	-118.514	-75.0560	-123.605	-121.105	-2.50000
4(d)	-123.594	-69.1066	-131.516	-128.118	-3.39761
4(e)	-116.356	-29.4637	-122.473	-120.856	-1.61678
4(f)	-123.710	-81.0062	-133.284	-130.259	-3.02522
4(g)	-129.236	-78.1883	-139.268	-131.175	-8.09349
4(h)	-117.568	-82.7142	-125.063	-124.373	-0.68934
4(i)	-120.181	-83.3620	-127.334	-125.341	-1.99252
4(j)	-121.750	-86.0709	-128.823	-127.814	-1.00954
4(k)	-134.939	-95.1971	-141.098	-135.154	-5.94470
4(l)	-120.624	-76.5033	-127.835	-124.713	-3.12210
4(m)	-122.049	-84.6806	-128.327	-126.562	-1.76459
4(n)	-128.162	-89.2231	-137.033	-129.115	-7.91857
4(o)	-122.928	-86.2262	-135.946	-126.397	**-9.54836**
GSK812397	**-140.600**	**-107.788**	**-162.632**	**-153.686**	-8.94604

Table S2. Molecular interactions analyses of imidazo[1,2-*a*]pyridin-3-yl derivatives ***4(a-o)*** and standard drug with CXCR4 target

Compound Name	Interaction	Bond Energy (kJ/mol)	Bond Length (Å)
4(a)	His 113 (N) – O (24)	-2.49659	3.10068
4(b)	Tyr 255 (O) – N (12)	-1.53804	3.29239
4(c)	His 203 (N) – O (24)	-2.50000	2.73422
4(d)	Tyr 255 (O) – N (11)	-0.72240	3.45552
	Arg 188 (N) – O (24)	-0.11967	3.02926
	His 203 (N) – O (24)	-2.50000	2.67462
	Arg 188 (N) – O (25)	-0.05553	3.57800
4(e)	Tyr 255 (O) – N (12)	-1.61678	3.27664
4(f)	Arg 188 (N) – O (24)	-0.08864	3.06312
	His 203 (N) – O (24)	-2.50000	2.63715
	Arg 188 (N) – N (25)	-0.43658	3.42724
4(g)	Tyr 255 (O) – N (11)	-0.46886	3.46287
	Tyr 255 (O) – N (12)	-1.43511	3.31298
	Arg 188 (N) – O (23)	-1.96889	3.02446
	Tyr 255 (O) – O (24)	-2.50000	3.09870
	Arg 188 (N) – N (25)	-0.46484	3.48990
	Thr 117 (O) – O (27)	-1.03903	3.39219
4(h)	Arg 188 (N) – O (23)	-0.68934	3.37597
4(i)	Arg 188 (N) – N (11)	-0.45503	3.50899
	Arg 188 (N) – O (23)	-1.46754	3.10181
	Gln 200 (N) – O (24)	-0.06995	3.07381
4(j)	Arg 188 (N) – O (23)	-0.73487	3.36232
	Tyr 256 (O) – O (26)	-0.27467	3.54507
4(k)	Arg 188 (N) – N (11)	-0.09408	3.58118
	Arg 188 (N) – O (23)	-0.91800	3.28765

	Tyr 255 (O) – N (26)	-2.47077	3.10585
	Tyr 255 (O) – O (28)	-2.46184	2.59580
4(l)	Arg 188 (N) – O (24)	-0.62210	3.37495
	Gln 200 (O) – O (26)	-2.50000	2.75218
4(m)	Arg 188 (N) – O (23)	-0.33636	3.53273
	Arg 188 (N) – N (11)	-1.42823	3.10997
4(n)	Tyr 255 (O) – N (11)	-2.50000	3.00195
	Glu 288 (O) – O (24)	-1.02597	2.87083
	Tyr 255 (O) – O (24)	-2.50000	2.62798
	Arg 188 (N) – O (23)	-1.89261	3.22148
4(o)	Tyr 255 (O) – N (11)	-2.50000	3.05410
	Arg 188 (N) – O (23)	-1.13822	2.96908
	Tyr 255 (O) – O (24)	-2.49754	2.59970
	Glu 288 (O) – O (24)	-2.35753	3.12849
	Arg 188 (N) – N (26)	-1.04858	3.36450
	Gln 200 (N) – O (28)	-0.00650	3.59325
GSK812397	His 113 (N) – O (15)	-2.44779	3.11044
	Cys 186 (O) – O (15)	-1.62570	3.27486
	Arg 188 (N) – N (18)	-2.37256	2.96048
	Tyr 45 (O) – N (27)	-2.50000	2.60997

Table S3. Docking scores (MolDock score, re-rank score, protein-ligand interaction, H-bond and steric score) of imidazo[1,2-*a*]pyridin-3-yl derivatives ***4(a-o)*** docked with GABAa target selected for screening

Compound Name	MolDock Score	Rerank Score (kJ/mol)	Interaction Energy (kJ/mol)	Steric	HBond (kJ/mol)
4(a)	-103.452	-69.5127	-116.442	-114.087	-2.35454
4(b)	-112.338	-73.2030	-118.926	-117.861	-1.06506
4(c)	-115.377	-84.4617	-122.956	-118.863	-4.09361
4(d)	-117.756	-57.2226	-125.317	-118.268	-7.04956
4(e)	-104.395	-76.7948	-117.214	-109.726	-7.48801
4(f)	-112.245	-54.2515	-122.505	-114.253	-8.25149
4(g)	-127.803	-78.4839	-132.640	-125.020	**-11.6198**
4(h)	-106.777	-66.9797	-118.490	-114.890	-3.60053
4(i)	-116.213	-61.6053	-120.648	-116.568	-4.08002
4(j)	-119.771	-64.9900	-125.664	-118.162	-7.50155
4(k)	**-127.861**	-66.0834	**-132.945**	-123.150	-9.79657
4(l)	-114.248	-68.4873	-122.295	-116.603	-5.69182
4(m)	-117.061	-62.8410	-121.504	-117.508	-3.99595
4(n)	-126.362	-69.3167	-132.274	-124.732	-7.54145
4(o)	-121.116	-67.3607	-129.283	-121.114	-8.16987
Alpidem	-124.136	-75.0110	-122.136	-120.423	-2.06300
Necopidem	-120.603	**-87.3621**	-131.208	**-126.952**	-4.25571
Saripidem	-107.125	-73.9209	-121.777	-116.984	-4.79349
Zolpidem	-107.739	-72.5033	-109.909	-102.360	-4.99674

Table S4. Molecular interactions analyses of imidazo[1,2-*a*]pyridin-3-yl derivatives ***4(a-o)*** and standard drugs with GABAa target

Compound Name	Interaction	Bond Energy (kJ/mol)	Bond Length (Å)
4(a)	Gln 185 (N) – N (11)	-0.01887	3.50074
	Glu 52 (O) – O (24)	-2.06127	3.18775
	Gln 185 (N) – O (24)	-0.20224	2.33505
	Arg 216 (N) – O (24)	-0.07216	3.48320
4(b)	Lys 274 (N) – N (11)	-0.39451	3.52110
	Lys 274 (N) – O (23)	-0.67055	3.02489
4(c)	Lys 274 (N) – N (11)	-0.76046	3.44791
	Lys 274 (N) – O (23)	-0.83315	2.98530
	Asp 48 (O) – O (25)	-2.50000	2.61034
4(d)	Ser 247 (N) – N (12)	-2.45906	3.10819
	Ala 248 (N) – O (23)	-0.94262	3.12790
	Ser 247 (N) – O (25)	-0.26395	3.38594
	Ala 248 (N) – O (25)	-2.50000	2.87342
	Asp 245 (O) – O (24)	-0.88393	3.42321
4(e)	Ser 51 (O) – N (12)	-2.50000	3.05332
	Val 50 (N) – O (24)	-2.48801	3.09671
	Val 50 (O) – O (24)	-2.50000	2.60752
4(f)	Ser 247 (N) – N (12)	-2.28007	3.14399
	Ala 248 (N) – O (23)	-0.62739	3.25713
	Ala 248 (N) – N (25)	-1.20660	3.35868
	Ser 247 (N) – O (26)	-1.63743	2.87303
	Ala 248 (N) – O (27)	-2.50000	2.66798
4(g)	Ala 249 (N) – O (23)	-1.02667	3.39467
	Ser 247 (N) – N (12)	-2.03881	2.96584
	Ser 247 (N) – N (25)	-1.23115	3.35377
	Asn 303 (N) – N (25)	-1.59348	3.28130
	Ser 247 (N) – O (27)	-2.49309	2.86640
	Tyr 299 (O) – O (27)	-2.43911	3.11218
	Asn 303 (N) – O (27)	-0.79745	2.40128
4(h)	Ser 247 (N) – N (12)	-2.50000	2.88144
	Ala 249 (N) – O (23)	-1.10053	3.37989
4(i)	Asp 245 (O) – O (24)	-1.34511	3.33098
	Ser 247 (N) – N (12)	-1.95609	3.20878
	Ala 248 (N) – O (23)	-0.77881	3.17806
4(j)	Ser 247 (N) – N (12)	-2.50000	3.09845
	Ala 248 (N) – O (23)	-0.94951	3.11150
	Ser 247 (N) – O (26)	-0.29519	3.40177
	Ala 248 (N) – O (26)	-2.50000	3.01517
	Asp 245 (O) – O (24)	-1.25685	3.34863
4(k)	Asp 245 (O) – O (24)	-1.71254	3.09974
	Ser 247 (N) – N (12)	-0.88481	3.42304
	Ala 248 (N) – O (23)	-1.06726	2.89624
	Ala 248 (N) – O (28)	-0.68707	3.15805
	Ala 249 (N) – O (28)	-2.50000	3.00206
	Ala 248 (N) – O (27)	-2.15662	2.60252
4(l)	Ser 247 (N) – N (12)	-2.50000	2.99292
	Ala 248 (N) – O (23)	-0.30269	3.40918

	Asp 245 (O) – O (26)	-2.50000	2.74660
	Ala 249 (N) – O (23)	-0.38914	3.52217
4(m)	Asp 245 (O) – O (24)	-1.18269	3.36346
	Ser 247 (N) – N (12)	-2.09955	3.18009
	Ala 248 (N) – O (23)	-0.71371	3.20069
4(n)	Asp 245 (O) – N (12)	-2.49769	3.10046
	Ala 248 (N) – O (23)	-0.96790	3.10685
	Ser 247 (N) – O (26)	-0.29448	3.38722
	Ala 248 (N) – O (26)	-2.50000	2.95489
	Asp 245 (O) – O (24)	-1.28138	3.34372
4(o)	Ser 247 (N) – N (12)	-2.24579	3.15084
	Ala 248 (N) – O (23)	-0.58669	3.27028
	Ala 248 (N) – N (26)	-1.16893	3.36621
	Ser 247 (N) – O (27)	-1.66846	2.89123
	Ala 248 (N) – O (28)	-2.50000	2.65312
Alpidem	Ser 247 (N) – N (6)	-1.38285	2.89728
	Ala 248 (N) – N (6)	-0.68007	3.46399
Necopidem	Ala 248 (N) – O (16)	-2.08813	2.55058
	Ser 247 (N) – N (8)	-2.16759	2.97824
Saripidem	Ala 248 (N) – O (15)	-2.09427	2.89916
	Ala 248 (N) – N (3)	-0.20955	3.55809
	Ser 247 (N) – N (8)	-2.48967	3.06991
Zolpidem	Ser 247 (O) – O (0)	-2.49674	2.59961
	Asn 303 (N) – O (0)	-2.50000	2.91458

Table S5. The active sites (cavities) (1-5) detected in different selected targets along with their volume and surface area

Target	Cavity Number	Volume (Å^3)	Surface area (Å^2)
**Human Farnesyl diphosphate synthase**	1	580.608	1182.72
	2	13.824	58.88
	3	13.824	60.16
	4	13.312	58.88
	5	10.752	51.20

Target	Cavity Number	Volume (Å^3)	Surface area (Å^2)
**Human Phosphodiesterase 3B**	1	3106.30	7082.24
	2	1007.62	3330.56
	3	614.91	2059.52
	4	531.46	1363.20
	5	330.24	921.60

Target	Cavity Number	Volume (Å^3)	Surface area (Å^2)
**GABAa**	1	194.56	732.16
	2	48.13	224.00
	3	39.42	154.88
	4	38.91	115.20
	5	32.77	133.12

Target	Cavity Number	Volume (Å^3)	Surface area (Å^2)
**CXCR4**	1	1274.370	2775.04
	2	116.224	375.04
	3	70.656	282.88
	4	41.472	185.60
	5	39.424	166.40

2. Frontier molecular orbital analysis

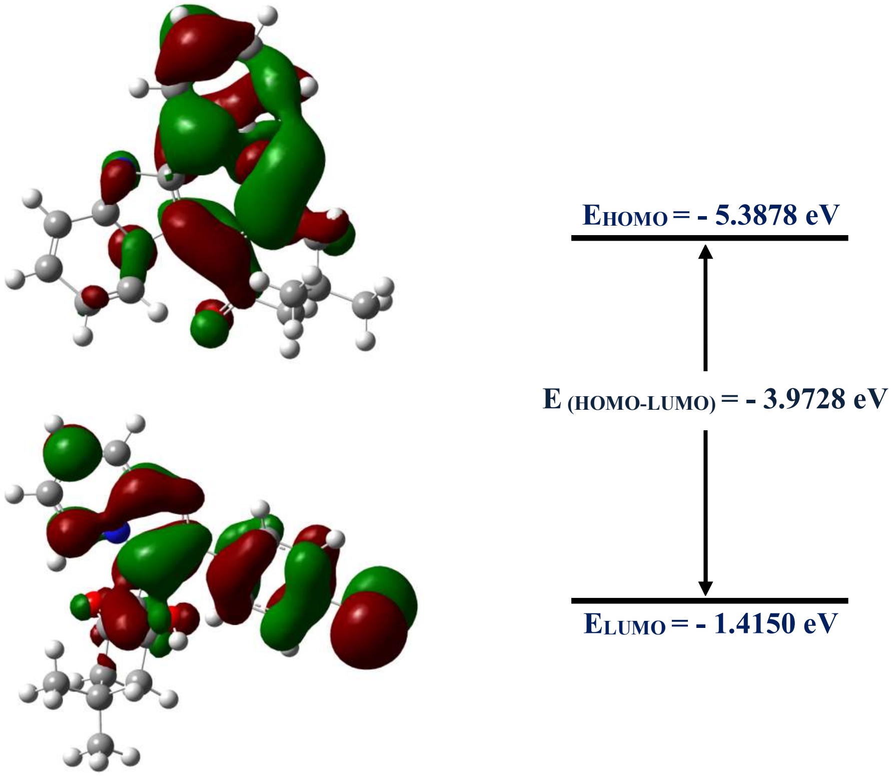

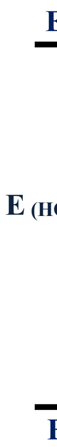

4(a)

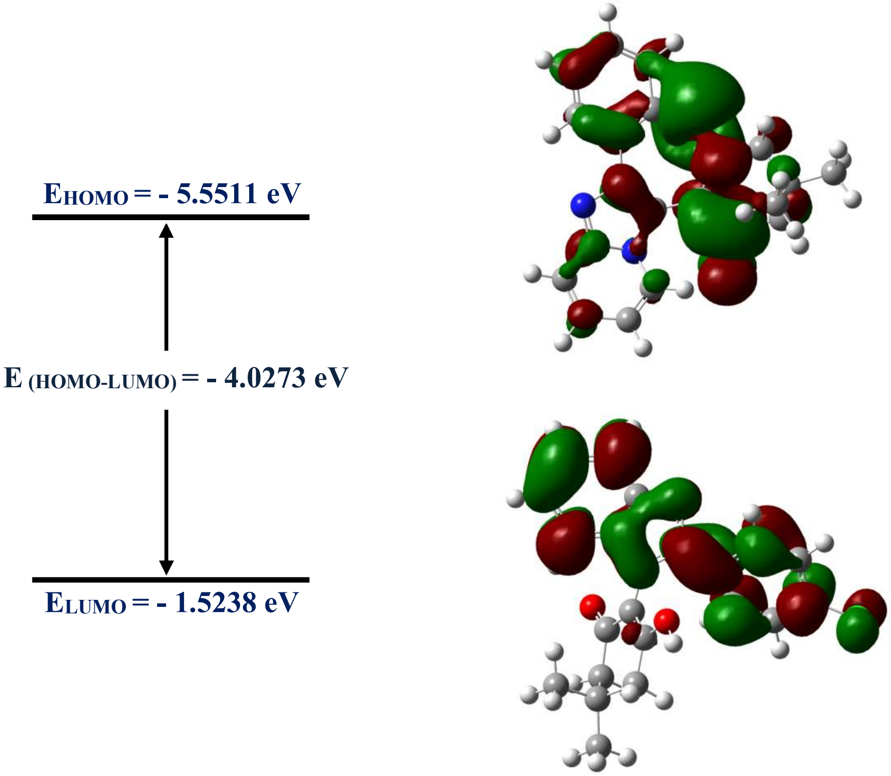

4(b)

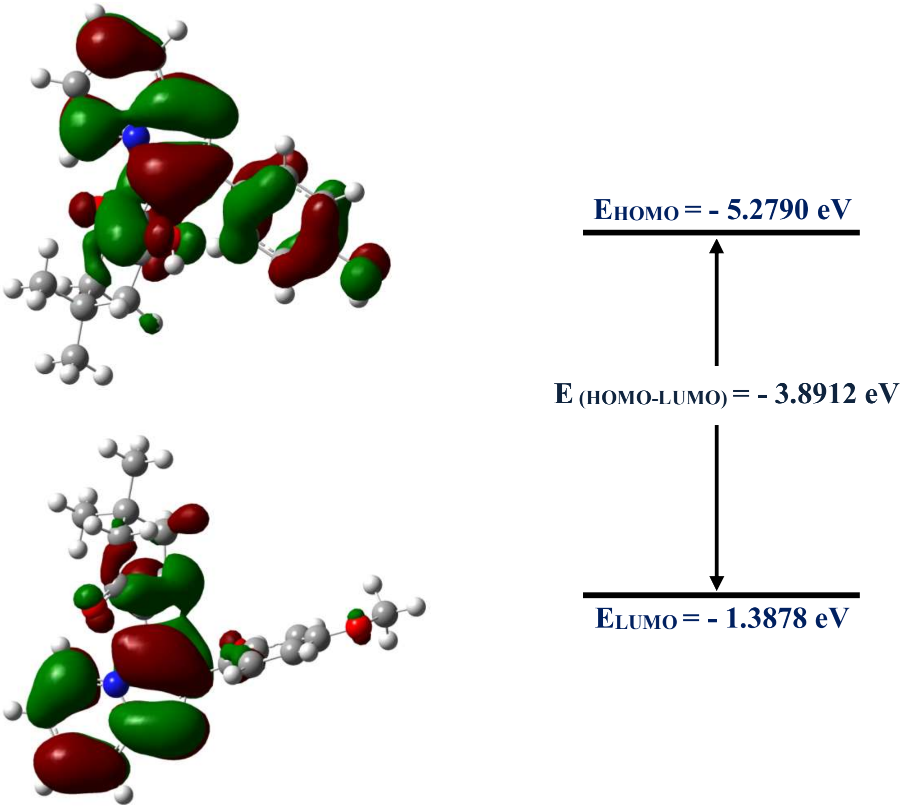
EHOMO = - 5.2790 eV
E (HOMO-LUMO) = - 3.8912 eV
ELUMO = - 1.3878 eV

4(c)

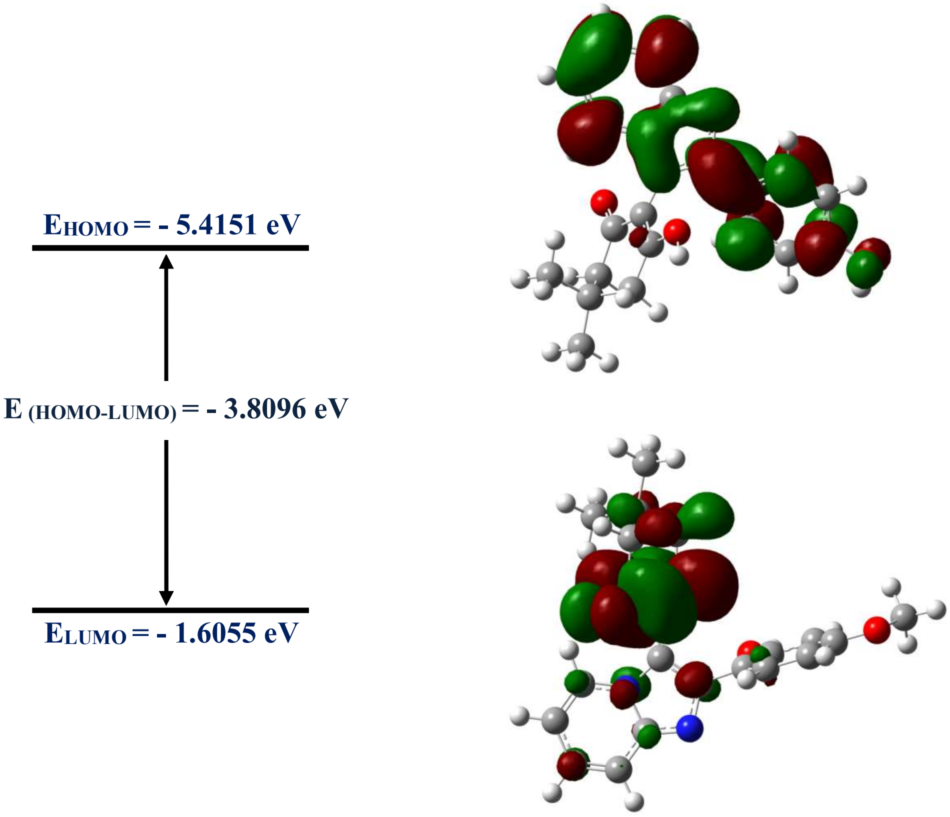
EHOMO = - 5.4151 eV
E (HOMO-LUMO) = - 3.8096 eV
ELUMO = - 1.6055 eV

4(d)

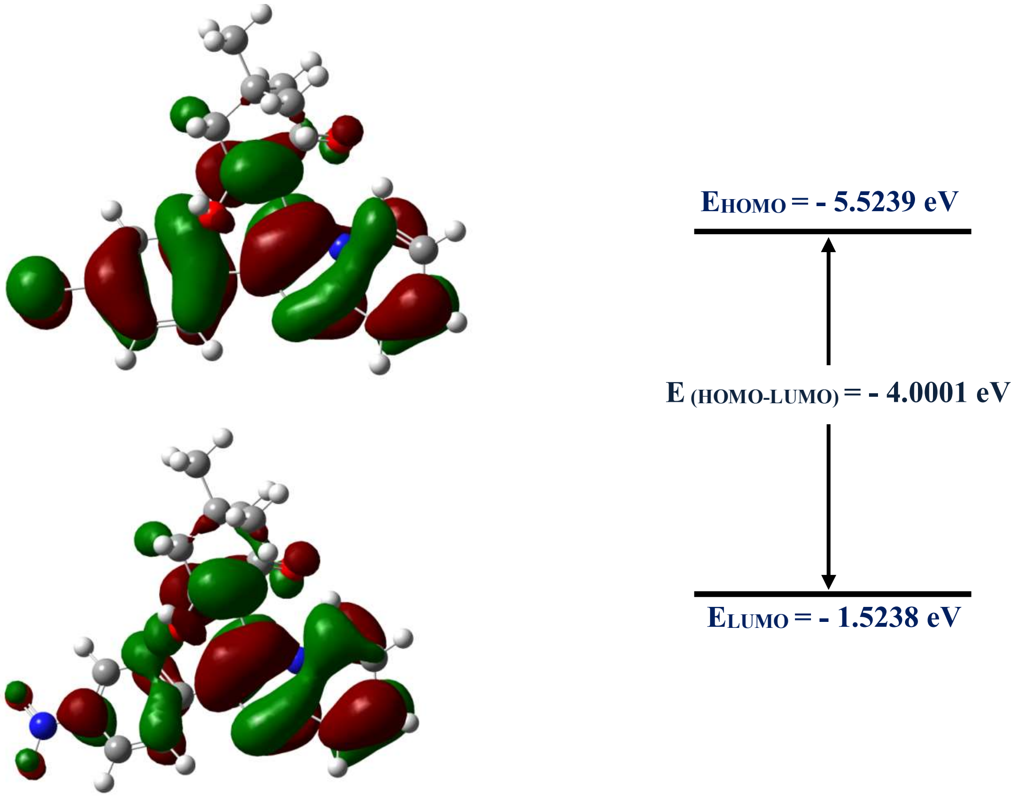
EHOMO = - 5.5239 eV
E (HOMO-LUMO) = - 4.0001 eV
ELUMO = - 1.5238 eV

4(e)

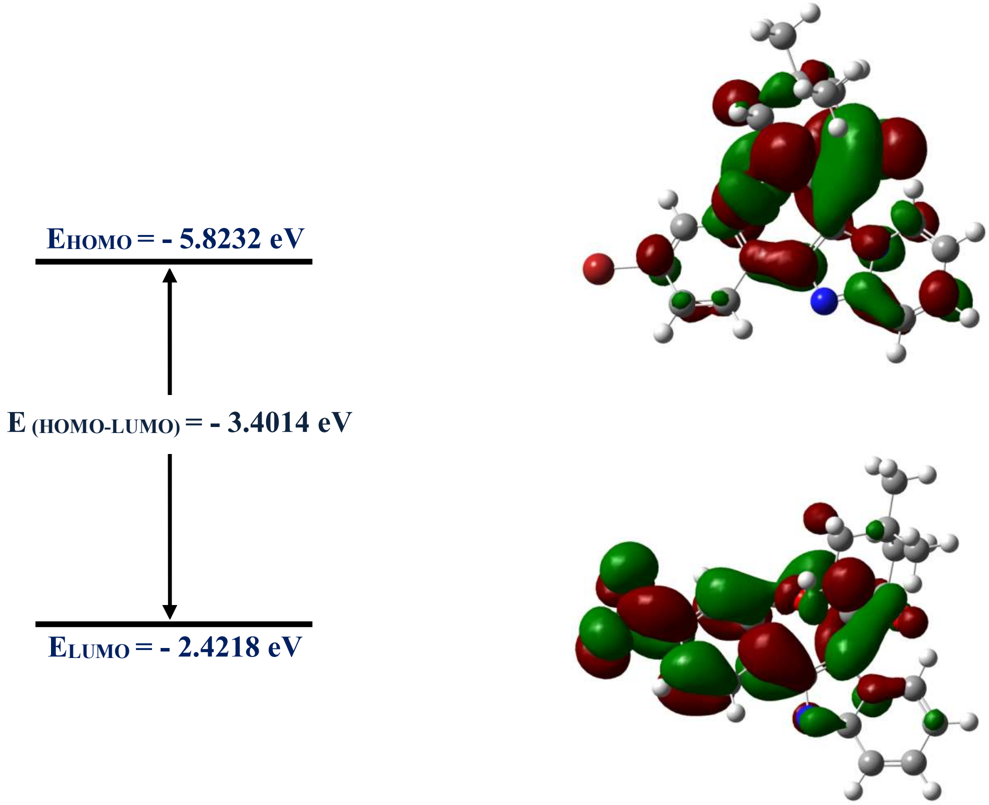
EHOMO = - 5.8232 eV
E (HOMO-LUMO) = - 3.4014 eV
ELUMO = - 2.4218 eV

4(f)

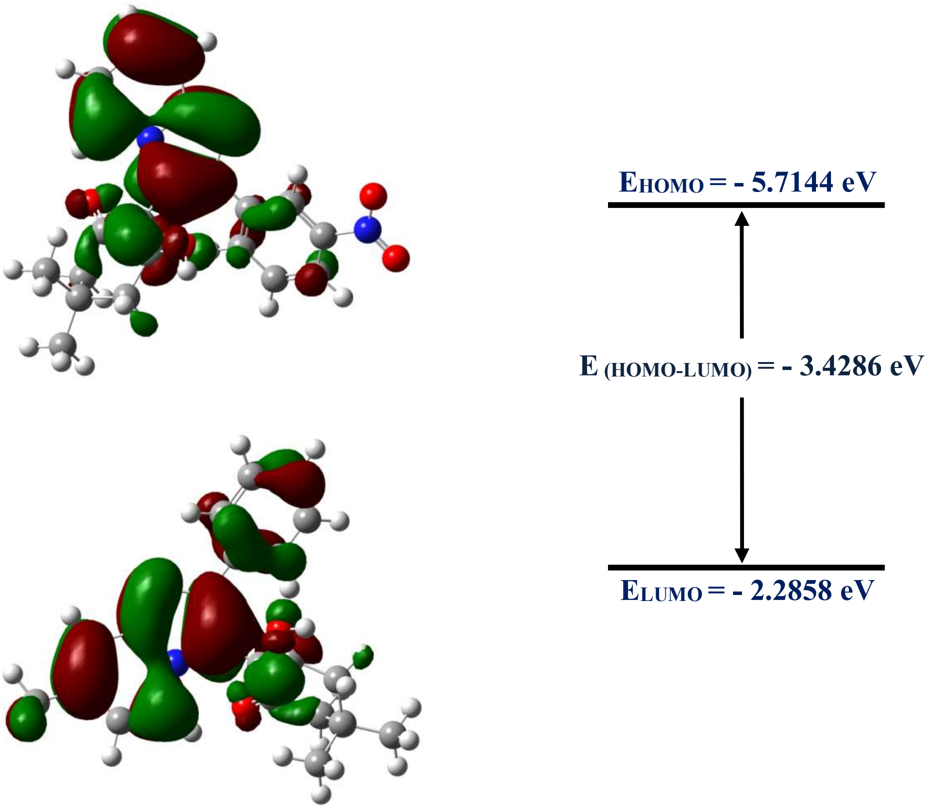
E_{HOMO} = - 5.7144 eV
$E_{(HOMO-LUMO)}$ = - 3.4286 eV
E_{LUMO} = - 2.2858 eV

4(g)

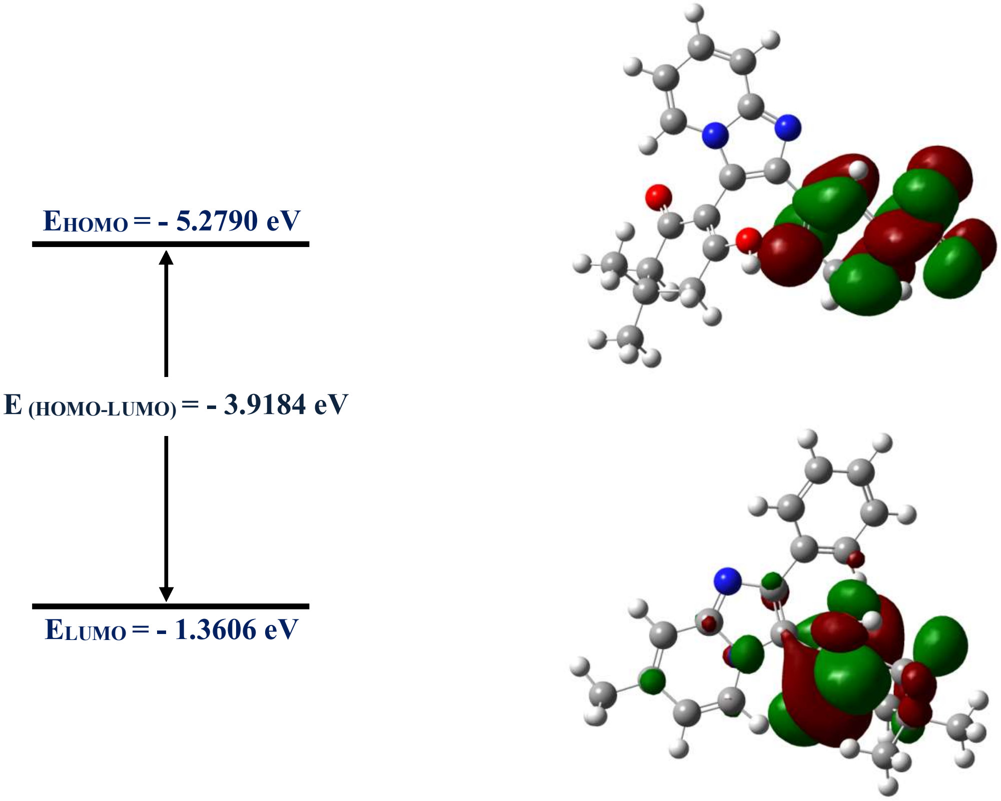
E_{HOMO} = - 5.2790 eV
$E_{(HOMO-LUMO)}$ = - 3.9184 eV
E_{LUMO} = - 1.3606 eV

4(h)

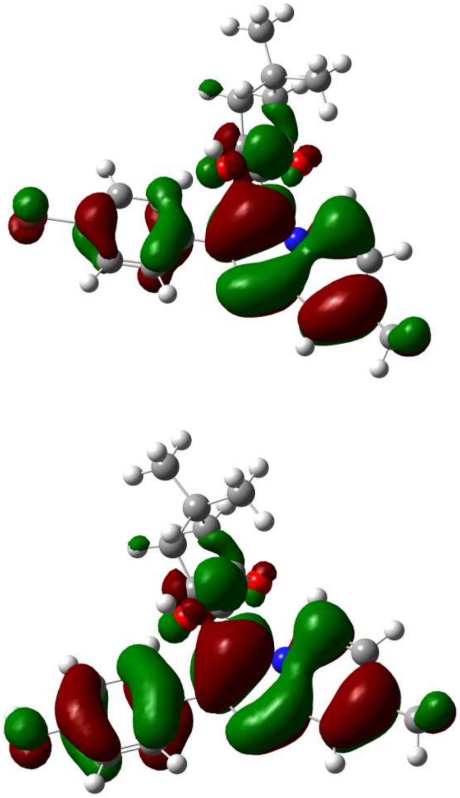

E_{HOMO} = - 5.4151 eV

$E_{(HOMO\text{-}LUMO)}$ = - 3.9185 eV

E_{LUMO} = - 1.4966 eV

4(i)

E_{HOMO} = - 5.1702 eV

$E_{(HOMO\text{-}LUMO)}$ = - 3.8096 eV

E_{LUMO} = - 1.3606 eV

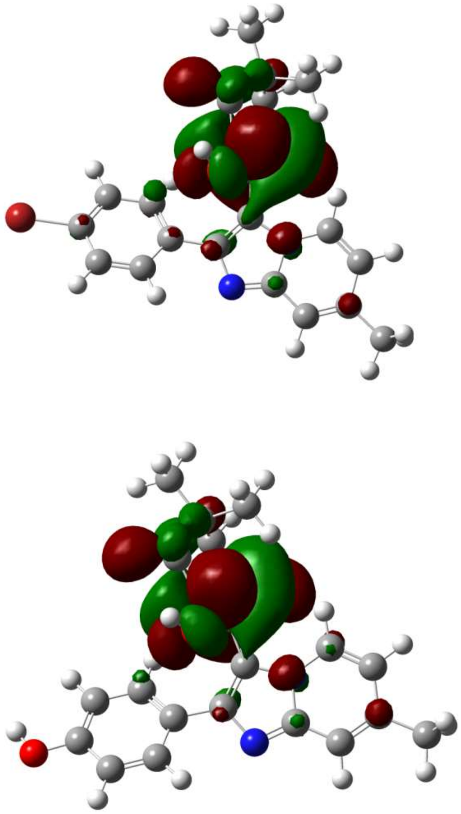

4(j)

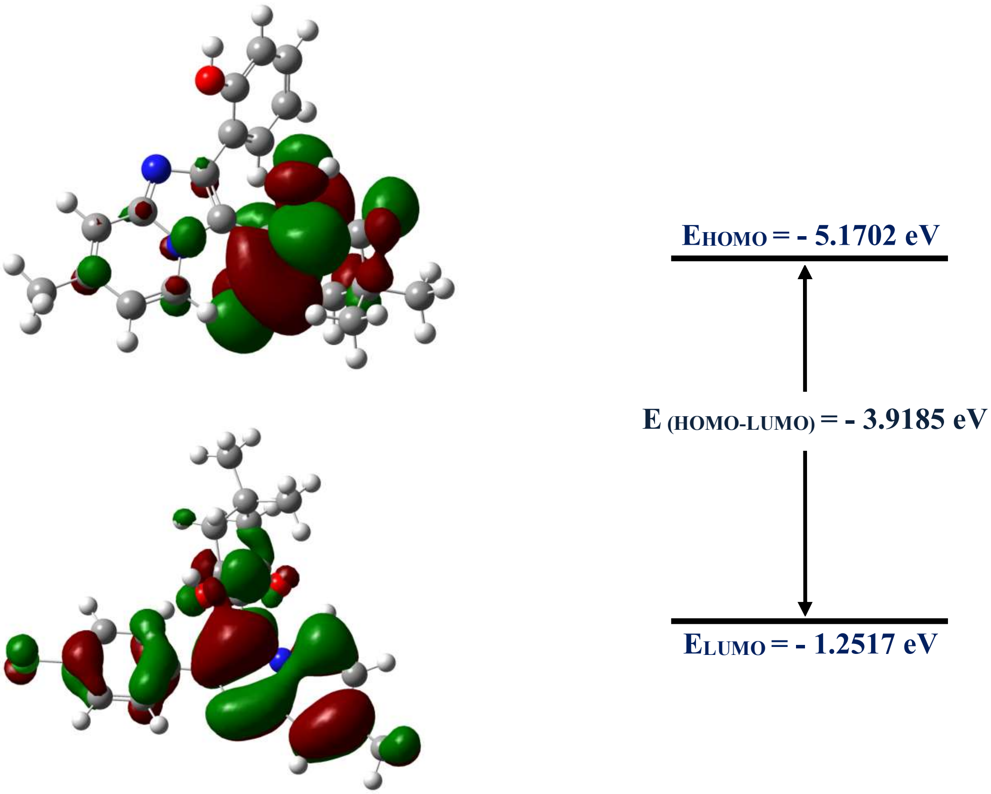
EHOMO = - 5.1702 eV
E (HOMO-LUMO) = - 3.9185 eV
ELUMO = - 1.2517 eV

4(l)

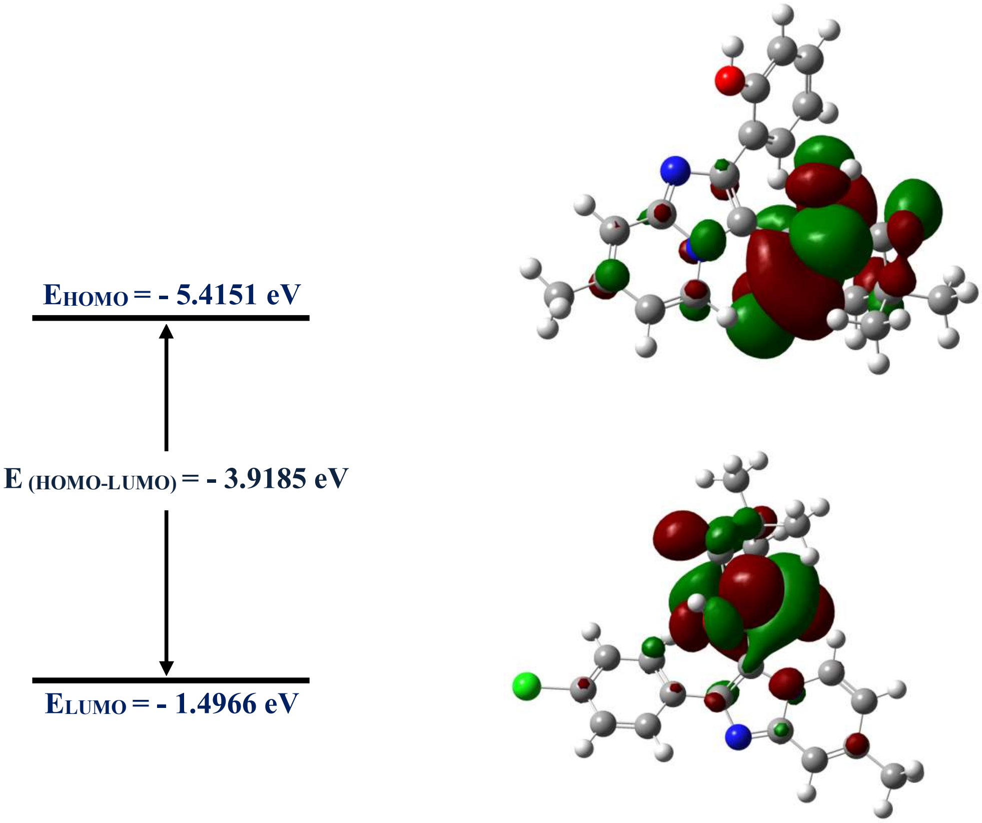
EHOMO = - 5.4151 eV
E (HOMO-LUMO) = - 3.9185 eV
ELUMO = - 1.4966 eV

4(m)

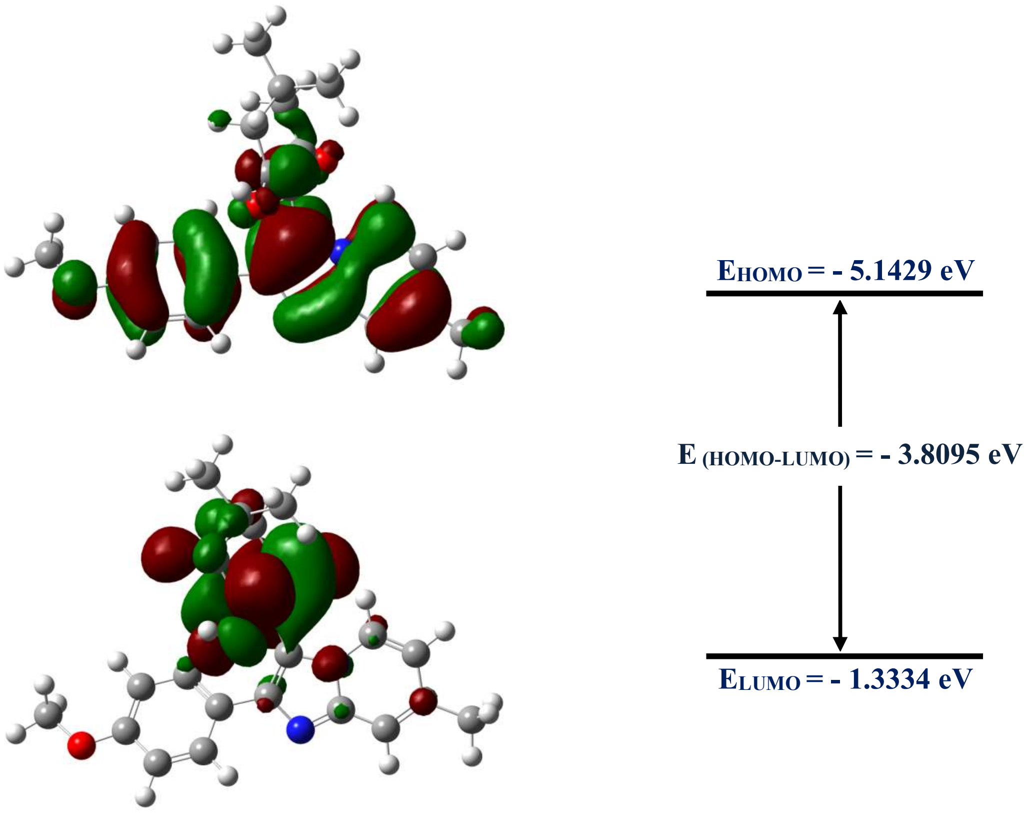

4(n)

Figure S2. The Optimized geometries with frontier molecular orbital of ***4(a-j)*** and ***4(l-n).***

❖ *Physical analyses data for imidazo[1,2-a]pyridin-3-yl derivatives 4(a-o)*

S1. Compound *4(a)*: *3-hydroxy-5,5-dimethyl-2-(2-phenylimidazo[1,2-a]pyridin-3-yl)cyclohex-2-enone*

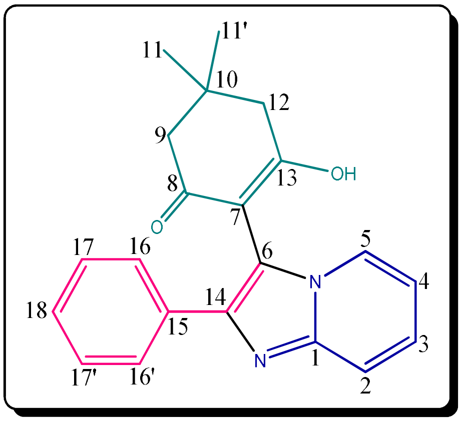

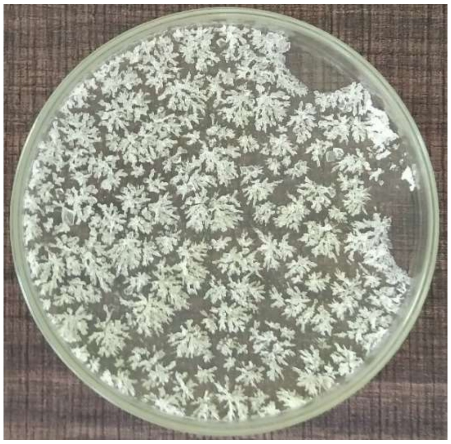

Compound ***4(a)*** was prepared in 84% yield from *2*-aminopydrine (1mmol) ***1(a)***, acetophenone (1mmol) ***2(a)***, dimedone (1mmol) *(3)*; White Solid; M.P.: 389−391°C;

FTIR (ATR, υ, cm^{-1}): 3330 (O-H str.), 3086 (C−H, sp^2), 2919 ($C{-}H_{Asym}$, sp^3), 2872 ($C{-}H_{Sym}$, sp^3), 1663 (C=C/C=N), 1624, 1573, 1487 (C⎓C ring str.), 1076 (C-O), 719 (CH_2);

^{1}H NMR: δ 1.03 (6H, s, CH_3), 2.17-2.29 (4H, m, CH_2), 7.08-7.18 (1H, m, CH), 7.33-7.52 (5H, m, CH), 7.60-7.68 (2H, m, CH), 8.80 (1H, m, CH);

^{13}C NMR: δ 28.2 (*C*−11, 11’), 34.0 (*C*−10), 43.7 (*C*−12), 50.2 (*C*−9), 112.7 (*C*−4), 116.7 (*C*−2), 126.8 (*C*−16, 16’), 127.3 (*C*−7), 127.8 (*C*−18), 128.4 (*C*−17,17’), 129.2 (*C*−5), 131.9 (*C*−15), 133.7 (*C*−6), 134.3 (*C*−3), 139.0 (*C*−14), 146.6 (*C*−1), 181.2 (*C*−13), 197.1 (*C*−8);

HRMS (ESI) *m/z*: 333.1507 $[M+H]^+$; Anal. Cald. for $C_{21}H_{20}N_2O_2$: C, 75.88; H, 6.06; N, 8.43 %. Found: C, 75.91; H, 6.09; N, 8.44 %.

S2. Compound *4(b)*: *2-(2-(4-chlorophenyl)imidazo[1,2-a]pyridin-3-yl)-3-hydroxy-5,5-dimethylcyclohex-2-enone*

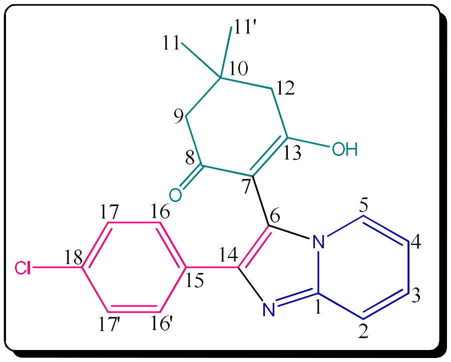

Compound ***4(b)*** was prepared in 83% yield from *2*-aminopydrine (1mmol) ***1(a)***, 4-chloro acetophenone (1mmol) ***2(b)***, dimedone (1mmol) ***(3)***; White Solid; M.P.: 384−386°C;

FTIR (ATR, υ, cm^{-1}): 3338 (O-H str.), 3078 (C−H, sp^2), 2921 (C−H_{Asym}, sp^3), 2866 (C−H_{Sym}, sp^3), 1653 (C=C/C=N), 1632, 1576, 1490 (C┄C ring str.), 1083 (C-O), 721 (CH_2), 609 (C-Cl);

^{1}H NMR: δ 1.03 (6H, s, CH_3), 2.17-2.32 (4H, m, CH_2), 7.13 (1H, m, CH), 7.37 (2H, m, CH), 7.54-7.62 (2H, m, CH), 7.66 (2H, m, CH), 8.78 (1H, m, CH);

^{13}C NMR: δ 28.2 (*C*−11, 11'), 34.0 (*C*−10), 43.7 (*C*−12), 50.2 (*C*−9), 112.7 (*C*−4), 116.7 (*C*−2), 127.3 (*C*−7), 128.5 (*C*−16, 16'), 128.7 (*C*−17,17'), 129.2 (*C*−5), 131.9 (*C*−15), 133.7 (*C*−18), 133.7 (*C*−3), 134.3 (*C*−6), 139.0 (*C*−14), 146.6 (*C*−11), 181.2 (*C*−13), 197.1 (*C*−8);

HRMS (ESI) *m/z*: 367.1110 $[M+H]^+$; Anal. Cald. for $C_{21}H_{19}ClN_2O_2$: C, 68.76; H, 5.22; N, 7.64 %. Found: C, 68.79; H, 5.25; N, 7.67 %.

S3. Compound *4(c)*: *3-hydroxy-2-(2-(4-hydroxyphenyl)imidazo[1,2-a]pyridin-3-yl)-5,5-dimethylcyclohex-2-enone*

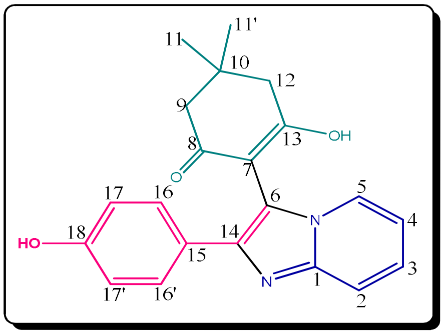

Compound ***4(c)*** was prepared in 93% yield from *2*-aminopydrine (1mmol) ***1(a)***, 4-Hydroxy acetophenone (1mmol) ***2(c)***, dimedone (1mmol) ***(3)***; White Solid; M.P.: 387−389°C;

FTIR (ATR, υ, cm^{-1}): 3452, 3342 (O-H str.), 3072 (C−H, sp^2), 2915 (C−H_{Asym}, sp^3), 2871 (C−H_{Sym}, sp^3), 1645 (C=C/C=N), 1633, 1552, 1448 (C⋯C ring str.), 1048 (C-O), 724 (CH_2);

^{1}H NMR: δ 1.03 (6H, s, CH_3), 2.17-2.32 (4H, m, CH_2), 7.10 (1H, m, CH), 7.20-7.28 (2H, m, CH), 7.52-7.59 (2H, m, CH), 7.62-7.65 (2H, m, CH), 8.76 (1H, m, CH);

^{13}C NMR: δ 28.2 (*C*−11, 11’), 34.0 (*C*−12), 43.7 (*C*−12), 50.2 (*C*−9), 112.7 (*C*−4), 115.7 (*C*−17, 17’), 116.7 (*C*−2), 127.3 (*C*−7), 129.0 (*C*−16, 16’), 129.2 (*C*−5), 131.9 (*C*−15), 133.7 (*C*−6), 134.3 (*C*−2), 139.0 (*C*−14), 146.6 (*C*−1), 157.4 (*C*−18), 181.2 (*C*−13), 197.1 (*C*−8);

HRMS (ESI) *m/z*: 349.1521 $[M+H]^+$; Anal. Cald. for $C_{21}H_{20}N_2O_3$: C, 72.40; H, 5.79; N, 8.04 %. Found: C, 72.43; H, 5.82; N, 8.07 %.

S4. Compound *4(d)*: *3-hydroxy-2-(2-(4-methoxyphenyl)imidazo[1,2-a]pyridin-3-yl)-5,5-dimethylcyclohex-2-enone*

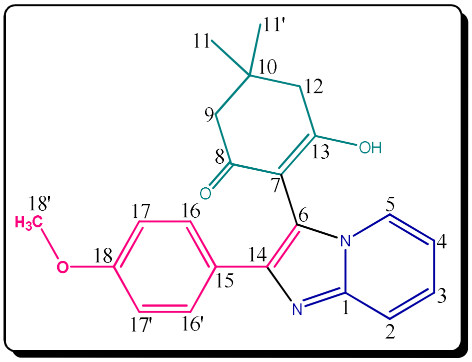

Compound ***4(d)*** was prepared in 91% yield from *2*-aminopydrine (1mmol) ***1(a)***, 4-Methoxy acetophenone (1mmol) ***2(d)***, dimedone (1mmol) ***(3)***; White Solid; M.P.: 388−390°C;

FTIR (ATR, υ, cm^{-1}): 3345 (O-H str.), 3085 (C−H, sp^2), 2923 (C−H_{Asym}, sp^3), 2867 (C−H_{Sym}, sp^3), 1649 (C=C/C=N), 1628, 1546, 1432 (C⎓C ring str.), 1242, 1048 (C-O), 717 (CH_2);

^{1}H NMR: δ 1.03 (6H, s, CH_3), 2.17-2.32 (4H, m, CH_2), 3.76 (3H, s, CH_3), 7.09 (1H, m, CH), 7.10-7.16 (2H, m, CH), 7.37-7.44 (2H, m, CH), 7.56-7.61 (2H, m, CH), 8.75 (1H, m, CH);

^{13}C NMR: δ 28.2 (*C*−11, 11'), 34.0 (*C*−10), 43.7 (*C*−12), 50.2 (*C*−9), 56.0 (*C*−18'), 112.7 (*C*−4), 114.3 (*C*−17, 17'), 116.7 (*C*−2), 127.3 (*C*−7), 129.0 (*C*−16, 16'), 129.2 (*C*−5), 131.9 (*C*−15), 133.7 (*C*−6), 134.3 (*C*−3), 139.0 (*C*−14), 146.6 (*C*−1), 159.8 (*C*−18), 181.2 (*C*−13), 197.1 (*C*−8);

HRMS (ESI) *m/z*: 363.1611 $[M+H]^+$; Anal. Cald. for $C_{22}H_{22}N_2O_3$: C, 72.91; H, 6.12; N, 7.73 %. Found: C, 72.93; H, 6.14; N, 7.75 %.

S5. Compound *4(e)*: *2-(2-(4-bromophenyl)imidazo[1,2-a]pyridin-3-yl)-3-hydroxy-5,5-dimethylcyclohex-2-enone*

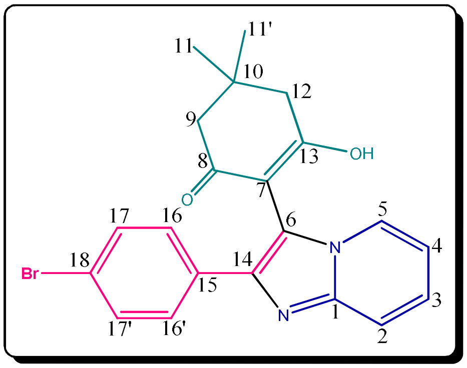

Compound ***4(e)*** was prepared in 85% yield from *2*-aminopydrine (1mmol) ***1(a)***, 4-Bromo acetophenone (1mmol) ***2(e)***, dimedone (1mmol) ***(3)***; White Solid; M.P.: 394−396°C;

FTIR (ATR, υ, cm^{-1}): 3346 (O-H str.), 3075 (C−H, sp^2), 2917 (C−H_{Asym}, sp^3), 2862 (C−H_{Sym}, sp^3), 1664 (C=C/C=N), 1631, 1544, 1428 (C┄C ring str.), 1046 (C-O), 724 (CH_2), 574 (C-Br);

^{1}H NMR: δ 1.03 (6H, s, CH_3), 2.17-2.32 (4H, m, CH_2), 7.10 (1H, m, CH), 7.19 (2H, m, CH), 7.54-7.68 (2H, m, CH), 7.79-7.82 (2H, m, CH), 8.79 (1H, m, CH);

^{13}C NMR: δ 28.2 (*C*−11, 11’), 34.0 (*C*−10), 43.7 (*C*−12), 50.2 (*C*−9), 112.7 (*C*−4), 116.7 (*C*−2), 122.3 (*C*−18), 127.3 (*C*−7), 128.2 (*C*−16, 16’), 129.2 (*C*−5), 131.7 (*C*−17, 17’), 131.9 (*C*−15), 133.7 (*C*−6), 134.3 (*C*−3), 139.0 (*C*−14), 146.6 (*C*−1), 181.2 (*C*−13), 197.1 (*C*−8);

HRMS (ESI) *m/z*: 411.0613 $[M+H]^+$; Anal. Cald. for $C_{21}H_{19}BrN_2O_2$: C, 61.33; H, 4.66; N, 6.81 %. Found: C, 61.37; H, 4.68; N, 6.84 %.

S6. Compound *4(f)*: *3-hydroxy-5,5-dimethyl-2-(2-(4-nitrophenyl)imidazo[1,2-a]pyridin-3-yl)cyclohex-2-enone*

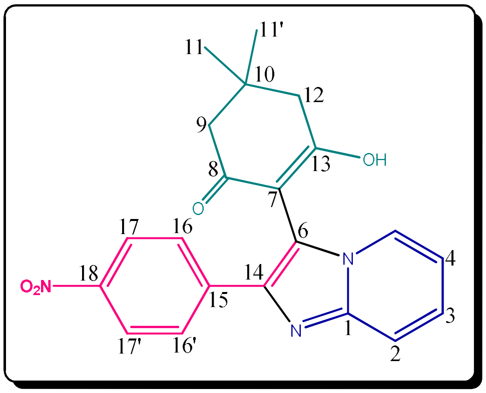

Compound *4(f)* was prepared in 89% yield from *2*-aminopydrine (1mmol) ***1(a)***, 4-Nitro acetophenone (1mmol) ***2(f)***, dimedone (1mmol) ***(3)***; White Solid; M.P.: 397−399°C;

FTIR (ATR, υ, cm^{-1}): 3342 (O-H str.), 3084 (C−H, sp^2), 2923 ($C{-}H_{Asym}$, sp^3), 2868 ($C{-}H_{Sym}$, sp^3), 1671 (C=C/C=N), 1636, 1546, 1423 (C⎓C ring str.), 1537 (N-O), 1043 (C-O), 715 (CH_2);

^1H NMR: δ 1.00 (6H, s, CH_3), 2.26-2.44 (4H, m, CH_2), 7.29 (1H, m, CH), 7.55-7.61 (2H, m, CH), 7.73-8.22 (4H, m, CH), 8.82 (1H, m, CH);

^{13}C NMR: δ 28.2 (*C*−11, 11'), 34.0 (*C*−10), 43.7 (*C*−12), 50.2 (*C*−9), 112.7 (*C*−4), 116.7 (*C*−2), 123.8 (*C*−17, 17'), 127.1 (*C*−16, 16'), 127.3 (*C*−7), 129.2 (*C*−5), 131.9 (*C*−15), 133.7 (*C*−6), 134.3 (*C*−3), 139.0 (*C*−14), 146.6 (*C*−1), 147.3 (*C*−18), 181.2 (*C*−13), 197.1 (*C*−8);

HRMS (ESI) *m/z*: 378.1405 $[M+H]^+$; Anal. Cald. for $C_{21}H_{19}N_3O_4$: C, 66.83; H, 5.07; N, 11.13 %. Found: C, 66.85; H, 5.09; N, 11.16 %.

S7. Compound *4(g)*: *3-hydroxy-5,5-dimethyl-2-(2-(3-nitrophenyl)imidazo[1,2-a]pyridin-3-yl)cyclohex-2-enone*

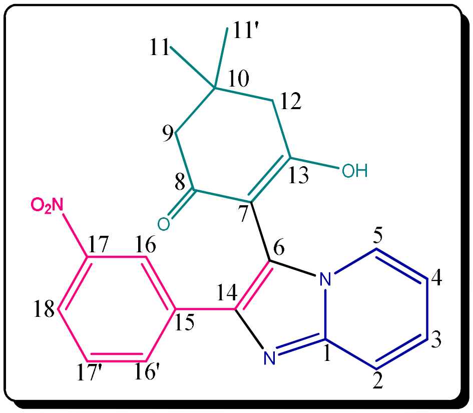

Compound ***4(g)*** was prepared in 92% yield from *2*-aminopydrine (1mmol) ***1(a)***, 3-Nitro acetophenone (1mmol) ***2(g)***, dimedone (1mmol) ***(3)***; White Solid; M.P.: 393−395°C;

FTIR (ATR, υ, cm^{-1}): 3424 (O-H str.), 3074 (C−H, sp^2), 2920 ($C-H_{Asym}$, sp^3), 2856 ($C-H_{Sym}$, sp^3), 1673 (C=C/C=N), 1635, 1520, 1421 (C⎓C ring str.), 1539 (N-O), 1048 (C-O), 720 (CH_2);

^1H NMR: δ 1.03 (6H, s, CH_3), 2.32-2.48 (4H, m, CH_2), 7.23 (1H, m, CH), 7.61 (1H, m, CH), 7.68-7.77 (2H, m, CH), 7.88-8.13 (2H, m, CH), 8.39 (1H, m, CH), 8.78 (1H, m, CH);

^{13}C NMR: δ 28.2 (*C*−11, 11’), 34.0 (*C*−12), 43.7 (*C*−12), 50.2 (*C*−9), 112.7 (*C*−4), 116.7 (*C*−2), 123.3 (*C*−18), 125.0 (*C*−16), 126.8 (*C*−16’), 127.3 (*C*−7), 129.0 (*C*−17), 129.2 (*C*−5), 132.0 (*C*−15), 133.7 (*C*−6), 134.3 (*C*−3), 139.0 (*C*−14), 145.3 (*C*−17), 146.6 (*C*−1), 181.2 (*C*−13), 197.1 (*C*−8);

HRMS (ESI) *m/z*: 378.1408 $[M+H]^+$; Anal. Cald. for $C_{21}H_{19}N_3O_4$: C, 66.83; H, 5.07; N, 11.13 %. Found: C, 66.86; H, 5.08; N, 11.18 %.

S8. Compound *4(h)*: *3-hydroxy-5,5-dimethyl-2-(7-methyl-2-phenylimidazo[1,2-a]pyridin-3-yl)cyclohex-2-enone*

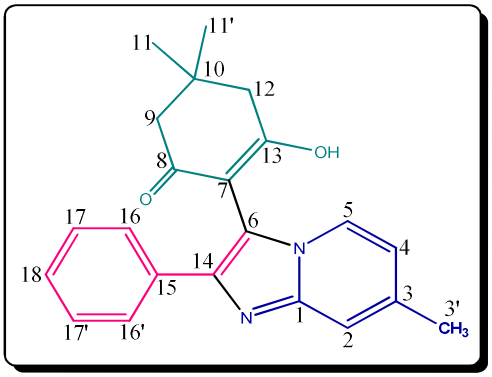

Compound ***4(h)*** was prepared in 82% yield from *2*-amino,4-methyl pydrine (1mmol) ***1(b)***, acetophenone (1mmol) ***2(a)***, dimedone (1mmol) ***(3)***; White Solid; M.P.: 395−397°C;

FTIR (ATR, υ, cm^{-1}): 3337 (O-H str.), 3089 (C−H, sp^2), 2934 ($C-H_{Asym}$, sp^3), 2861, 2855 ($C-H_{Sym}$, sp^3), 1669 (C=C/C=N), 1632, 1542, 1419 (C⋯C ring str.), 1047 (C-O), 719 (CH_2);

^1H NMR: δ 1.03 (6H, s, CH_3), 2.17-2.26 (4H, m, CH_2), 2.34 (3H, s, CH_3), 7.12 (1H, m, CH), 7.32-7.38 (3H, m, CH), 7.42 (1H, m, CH), 7.45-7.52 (2H, m, CH), 8.77 (1H, m, CH);

^{13}C NMR: δ 21.6 (*C*−3'), 28.2 (*C*−11, 11'), 34.0 (*C*−10), 43.7 (*C*−12), 50.2 (*C*−9), 114.8 (*C*−2), 115.1 (*C*−4), 126.8 (*C*−16, 16'), 127.3 (*C*−7), 127.8 (*C*−18), 128.4 (*C*−17, 17') 129.2 (*C*−5), 131.9 (*C*−15), 133.7 (*C*−6), 138.1 (*C*−3), 139.0 (*C*−14), 146.6 (*C*−1), 181.2 (*C*−13), 197.1 (*C*−8);

HRMS (ESI) *m/z*: 347.1705 $[M+H]^+$; Anal. Cald. for $C_{22}H_{22}N_2O_2$: C, 76.28; H, 6.40; N, 8.09 %. Found: C, 76.31; H, 6.43; N, 8.12 %.

S9. Compound *4(i)*: *2-(2-(4-bromophenyl)-7-methylimidazo[1,2-a]pyridin-3-yl)-3-hydroxy-5,5-dimethylcyclohex-2-enone*

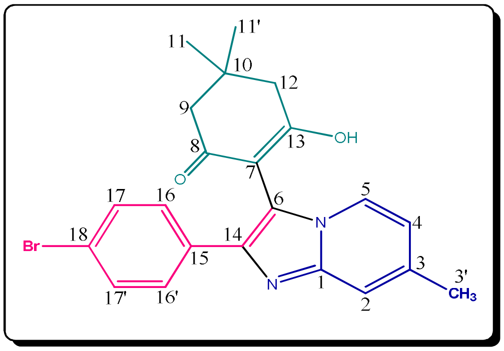

Compound ***4(i)*** was prepared in 87% yield from *2*-amino,4-methyl pydrine (1mmol) ***1(b)***, 4-Bromo acetophenone (1mmol) ***2(e)***, dimedone (1mmol) ***(3)***; White Solid; M.P.: 396−398°C;

FTIR (ATR, υ, cm^{-1}): 3390 (O-H str.), 3083 (C−H, sp^2), 2921 ($C{-}H_{Asym}$, sp^3), 2858, 2834 ($C{-}H_{Sym}$, sp^3), 1667 (C=C/C=N), 1639, 1541, 1422 (C⎓C ring str.), 1047 (C-O), 723 (CH_2), 578 (C-Br);

^{1}H NMR: δ 1.03 (6H, s, CH_3), 2.17-2.25 (4H, m, CH_2), 2.30 (3H, s, CH_3), 6.97-7.16 (2H, m, CH), 7.40-7.78 (4H, m, CH), 8.76 (1H, m, CH);

^{13}C NMR: δ 21.6 (*C*−3'), 28.2 (*C*−11, 11'), 34.0 (*C*−10), 43.7 (*C*−12), 50.2 (*C*−9), 114.8 (*C*−2), 115.1 (*C*−4), 122.3 (*C*−18), 127.3 (*C*−7), 128.2 (*C*−16, 16'), 129.2 (*C*−5), 131.7 (*C*−17, 17'), 131.9 (*C*−15), 133.7 (*C*−6), 138.1 (*C*−3), 139.0 (*C*−14), 146.6 (*C*−1), 181.2 (*C*−13), 197.1 (*C*−8);

HRMS (ESI) *m/z*: 425.0812 $[M+H]^+$; Anal. Cald. for $C_{22}H_{21}BrN_2O_2$: C, 62.13; H, 4.98; N, 6.59 %. Found: C, 62.15; H, 5.01; N, 6.62 %.

S10. Compound *4(j)*: *3-hydroxy-2-(2-(4-hydroxyphenyl)-7-methylimidazo[1,2-a]pyridin-3-yl)-5,5-dimethylcyclohex-2-enone*

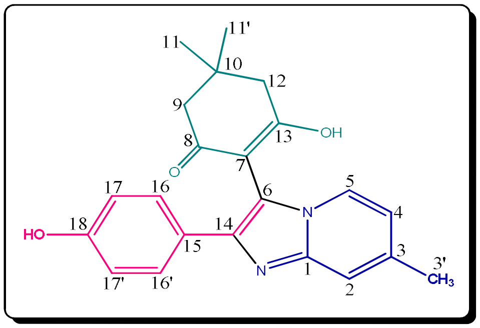

Compound ***4(j)*** was prepared in 94% yield from *2*-amino,4-methyl pydrine (1mmol) ***1(b)***, 4-Hydroxy acetophenone (1mmol) ***2(c)***, dimedone (1mmol) ***(3)***; White Solid; M.P.: 386−388°C;

FTIR (ATR, υ, cm^{-1}): 3449, 3387 (O-H str.), 3084 (C−H, sp^2), 2917 (C−H_{Asym}, sp^3), 2851, 2835 (C−H_{Sym}, sp^3), 1659 (C=C/C=N), 1639, 1545, 1419 (C⩵C ring str.), 1049 (C-O), 722 (CH_2);

^1H NMR: δ 1.03 (6H, s, CH_3), 2.15-2.24 (4H, m, CH_2), 2.30 (3H, s, CH_3), 7.03-7.13 (2H, m, CH), 7.20-7.61 (4H, m, CH), 8.70 (1H, m, CH);

^{13}C NMR: δ 21.6 (*C*−3'), 28.2 (*C*−11, 11'), 34.0 (*C*−10), 43.7 (*C*−12), 50.2 (*C*−9), 114.8 (*C*−2), 115.1 (*C*−4), 115.7 (*C*−17, 17'), 127.3 (*C*−7), 129.0 (*C*−16, 16'), 129.2 (*C*−5), 131.9 (*C*−15), 133.7 (*C*−6), 138.1 (*C*−3), 139.0 (*C*−14), 146.6 (*C*−1), 157.4 (*C*−18), 181.2 (*C*−13), 197.1 (*C*−8);

HRMS (ESI) *m/z*: 363.1602 $[M+H]^+$; Anal. Cald. for $C_{22}H_{22}N_2O_3$: C, 72.91; H, 6.12; N, 7.73 %. Found: C, 72.94; H, 6.16; N, 7.75 %.

S11. Compound *4(k)*: *3-hydroxy-5,5-dimethyl-2-(7-methyl-2-(3-nitrophenyl)imidazo[1,2-a]pyridin-3-yl)cyclohex-2-enone*

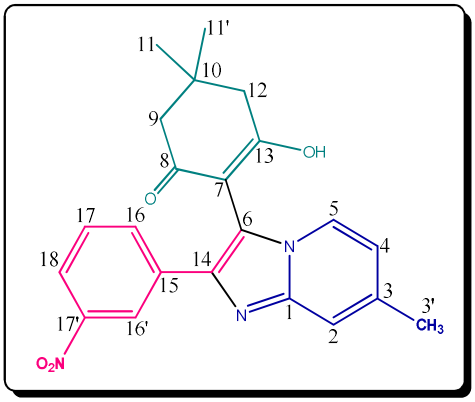

Compound ***4(k)*** was prepared in 94% yield from *2*-amino,4-methyl pydrine (1mmol) ***1(b)***, 3-Nitro acetophenone (1mmol) ***2(g)***, dimedone (1mmol) ***(3)***; White Solid; M.P.: 390−392°C;

FTIR (ATR, υ, cm^{-1}): 3392 (O-H str.), 3072 (C−H, sp^2), 2919 (C−H_{Asym}, sp^3), 2851, 2829 (C−H_{Sym}, sp^3), 1669 (C=C/C=N), 1637, 1545, 1421 (C┉C ring str.), 1538 (N-O), 1047 (C-O) 723 (CH_2);

^{1}H NMR: δ 1.03 (6H, s, CH_3), 2.25-2.32 (4H, m, CH_2), 2.33 (3H, s, CH_3), 7.15-7.24 (2H, m, CH), 7.57-8.39 (4H, m, CH), 8.72 (1H, m, CH);

^{13}C NMR: δ 21.6 (*C*−3'), 28.2 (*C*−11, 11'), 34.0 (*C*−10), 43.7 (*C*−12), 50.2 (*C*−9), 114.8 (*C*−2), 115.1 (*C*−4), 123.3 (*C*−18), 125.0 (*C*−16'), 126.8 (*C*−16), 127.3 (*C*−7), 129.0 (*C*−17), 129.2 (*C*−5), 132.0 (*C*−15), 133.7 (*C*−6), 138.1 (*C*−3), 139.0 (*C*−14), 145.3 (*C*−17'), 146.6 (*C*−1), 181.2 (*C*−13), 197.1 (*C*−8);

HRMS (ESI) *m/z*: 392.1507 $[M+H]^+$; Anal. Cald. for $C_{22}H_{21}N_3O_4$: C, 67.51; H, 5.41; N, 10.74 %. Found: C, 67.54; H, 5.43; N, 10.76 %.

S12. Compound *4(l)*: *3-hydroxy-2-(2-(2-hydroxyphenyl)-7-methylimidazo[1,2-a]pyridin-3-yl)-5,5-dimethylcyclohex-2-enone*

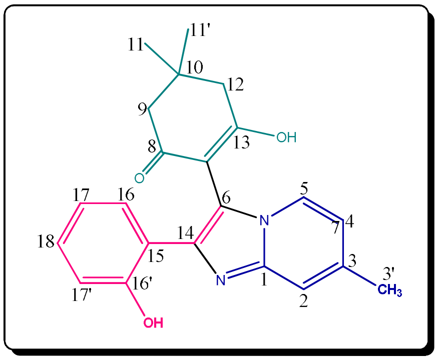

Compound ***4(l)*** was prepared in 96% yield from *2*-amino,4-methyl pydrine (1mmol) ***1(b)***, 2-Hydroxy acetophenone (1mmol) ***2(h)***, dimedone (1mmol) ***(3)***; White Solid; M.P.: 389−391°C;

FTIR (ATR, υ, cm^{-1}): 3451, 3389 (O-H str.), 3083 (C−H, sp^2), 2917 ($C{-}H_{Asym}$, sp^3), 2848, 2827 ($C{-}H_{Sym}$, sp^3), 1667 (C=C/C=N), 1631, 1542, 1423 (C⩦C ring str.), 1048 (C-O), 717 (CH_2);

^{1}H NMR: δ 1.03 (6H, s, CH_3), 2.24-2.35 (4H, m, CH_2), 2.30 (3H, s, CH_3), 7.03-7.13 (2H, m, CH), 7.17-8.12 (4H, m, CH), 8.70 (1H, m, CH);

^{13}C NMR: δ 21.6 (*C*−3’), 28.2 (*C*−11, 11’), 34.0 (*C*−10), 43.7 (*C*−12), 50.2 (*C*−9), 114.8 (*C*−2), 115.1 (*C*−4), 116.8 (*C*−17’), 119.6 (*C*−15), 125.5 (*C*−16), 127.3 (*C*−7), 128.4 (*C*−17), 129.2 (*C*−5), 129.4 (*C*−18), 133.7 (*C*−6), 138.1 (*C*−3), 139.0 (*C*−14), 146.6 (*C*−1), 150.0 (*C*−16’), 181.2 (*C*−13), 197.1 (*C*−8);

HRMS (ESI) *m/z*: 363.1608 $[M+H]^+$; Anal. Cald. for $C_{22}H_{22}N_2O_4$: C, 72.91; H, 6.12; N, 7.73 %. Found: C, 72.93; H, 6.15; N, 7.76 %.

S13. Compound *4(m)*: *3-hydroxy-2-(2-(4-methoxyphenyl)-7-methylimidazo[1,2-a]pyridin-3-yl)-5,5-dimethylcyclohex-2-enone*

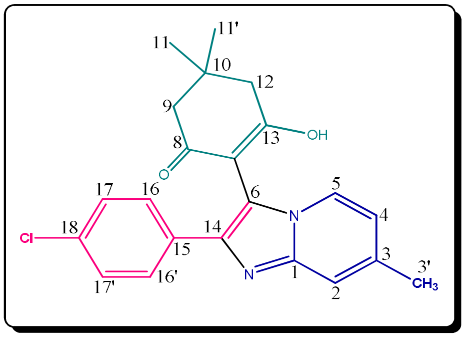

Compound ***4(m)*** was prepared in 89% yield from *2*-aminopydrine (1mmol) ***1(a)***, 4-chloro acetophenone (1mmol) ***2(b)***, dimedone (1mmol) ***(3)***; White Solid; M.P.: 395−397 °C;

FTIR (ATR, υ, cm^{-1}): 3385 (O-H str.), 3082 (C−H, sp^2), 2918 (C−H_{Asym}, sp^3), 2856, 2831 (C−H_{Sym}, sp^3), 663 (C=C/C=N), 1635, 1538, 1419 (C⩦C ring str.), 1049 (C-O), 719 (CH_2), 609 (C-Cl);

^{1}H NMR: δ 1.03 (6H, s, CH_3), 2.25-2.31 (4H, m, CH_2), 2.33 (3H, s, CH_3), 7.07-7.15 (2H, m, CH), 7.59-7.78 (4H, m, CH), 8.75-8.78 (1H, m, CH);

^{13}C NMR: δ 21.6 (*C*−3'), 28.2 (*C*−11, 11'), 34.0 (*C*−10), 43.7 (*C*−12), 50.2 (*C*−9), 114.8 (*C*−2), 115.1 (*C*−4), 127.3 (*C*−7), 128.5 (*C*−16, 16'), 128.7 (*C*−17, 17'), 129.2 (*C*−5), 131.9 (*C*−15), 133.7 (*C*−6, 18), 138.1 (*C*−3), 139.0 (*C*−14), 146.6 (*C*−1), 181.2 (*C*−13), 197.1 (*C*−8).

HRMS (ESI) *m/z*: 381.1314 $[M+H]^+$; Anal. Cald. for $C_{22}H_{21}ClN_2O_2$: C, 69.38; H, 5.56; N, 9.31 %. Found: C, 69.41; H, 5.59; N, 9.34 %.

S14. Compound *4(n)*: *3-hydroxy-2-(2-(4-methoxyphenyl)-7-methylimidazo[1,2-a]pyridin-3-yl)-5,5-dimethylcyclohex-2-enone*

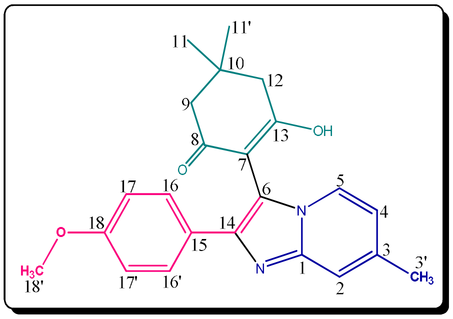

Compound ***4(n)*** was prepared in 93% yield from *2*-aminopydrine (1mmol) ***1(a)***, 4-methoxy acetophenone (1mmol) ***2(d)***, dimedone (1mmol) ***(3)***; White Solid; M.P.: 389−391°C;

FTIR (ATR, υ, cm^{-1}): 3435 (O-H str.), 3079 (C−H, sp^2), 2921 ($C-H_{Asym}$, sp^3), 2858, 2836 ($C-H_{Sym}$, sp^3), 1659 (C=C/C=N), 1630, 1536, 1417 (C┅C ring str.), 1541(N-O), 1254, 1048 (C-O), 721 (CH_2);

^{1}H NMR (400 MHz, $CDCl_3$): δ_H ppm 1.03 (6H, s, CH_3), 2.22-2.27 (4H, m, CH_2), 2.29 (3H, s, CH_3), 3.75 (3H, s, CH_3), 7.01-7.10 (2H, m, CH), 7.14-7.74 (4H, m, CH), 8.66-8.69 (1H, m, CH);

^{13}C NMR: δ 21.6 (*C*−3'), 28.2 (*C*−11, 11'), 34.0 (*C*−10), 43.7 (*C*−12), 50.2 (*C*−9), 56.0 (*C*−18'), 114.3 (*C*−17, 17'), 114.8 (*C*−2), 115.1 (*C*−4), 127.3 (*C*−7), 129.0 (*C*−16, 16'), 129.2 (*C*−5), 131.9 (*C*−15), 133.7 (*C*−6), 138.1 (*C*−3), 139.0 (*C*−14), 146.6 (*C*−1), 159.8 (*C*−18), 181.2 (*C*−13), 197.1 (*C*−8).

HRMS (ESI) *m/z*: 377.1801 $[M+H]^+$; Anal. Cald. for $C_{23}H_{24}N_2O_3$: C, 73.38; H, 6.43; N, 7.44 %. Found: C, 73.43; H, 6.46; N, 7.45 %.

S15. Compound *4(o)*: *3-hydroxy-5,5-dimethyl-2-(7-methyl-2-(4-nitrophenyl)imidazo[1,2-a]pyridin-3-yl)cyclohex-2-enone*

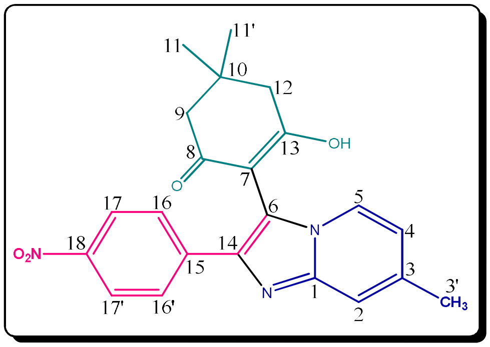

Compound *4(o)* was prepared in 91% yield from *2*-aminopydrine (1mmol) ***1(a)***, 4-nitro acetophenone (1mmol) ***2(f)***, dimedone (1mmol) ***(3)***; White Solid; M.P.: 396−398°C;

FTIR (ATR, υ, cm^{-1}): 3341 (O-H str.), 3081 (C−H, sp^2), 2926 ($C{-}H_{Asym}$, sp^3), 2863, 2848 ($C{-}H_{Sym}$, sp^3), 1665 (C=C/C=N), 1636, 1547, 1421 (C$\cdots$C ring str.), 1541(N-O), 1049 (C-O), 718 (CH_2);

^{1}H NMR (400 MHz, $CDCl_3$): δ_H ppm 1.00 (6H, s, CH_3), 2.24-2.30 (4H, m, CH_2), 2.33 (3H, s, CH_3), 7.10-7.30 (2H, m, CH), 7.71-8.24 (4H, m, CH), 8.74-8.76 (1H, m, CH);

^{13}C NMR (100 MHz, $CDCl_3$): δ 21.6 (*C*−3'), 28.2 (*C*−11, 11'), 34.0 (*C*−10), 43.7 (*C*−12), 50.2 (*C*−9), 114.8 (*C*−2), 115.1 (*C*−4), 123.8 (*C*−17, 17'), 127.1 (*C*−16, 16'), 127.3 (*C*−7), 129.2 (*C*−5), 131.9 (*C*−15), 133.7 (*C*−6), 138.1 (*C*−3), 139.0 (*C*−14), 146.6 (*C*−1), 147.3 (*C*−18), 181.2 (*C*−13), 197.1 (*C*−8);

HRMS (ESI) *m/z*: 392.1507 $[M+H]^+$; Anal. Cald. for $C_{22}H_{21}N_3O_4$: C, 67.51; H, 5.41; N, 10.74 %. Found: C, 67.54; H, 5.43; N, 10.77 %.

Figure S1. The FT-IR Spectrum of 3-hydroxy-5,5-dimethyl-2-(2-phenylimidazo[1,2-*a*]pyridin-3-yl)cyclohex-2-enone ***4(a)***

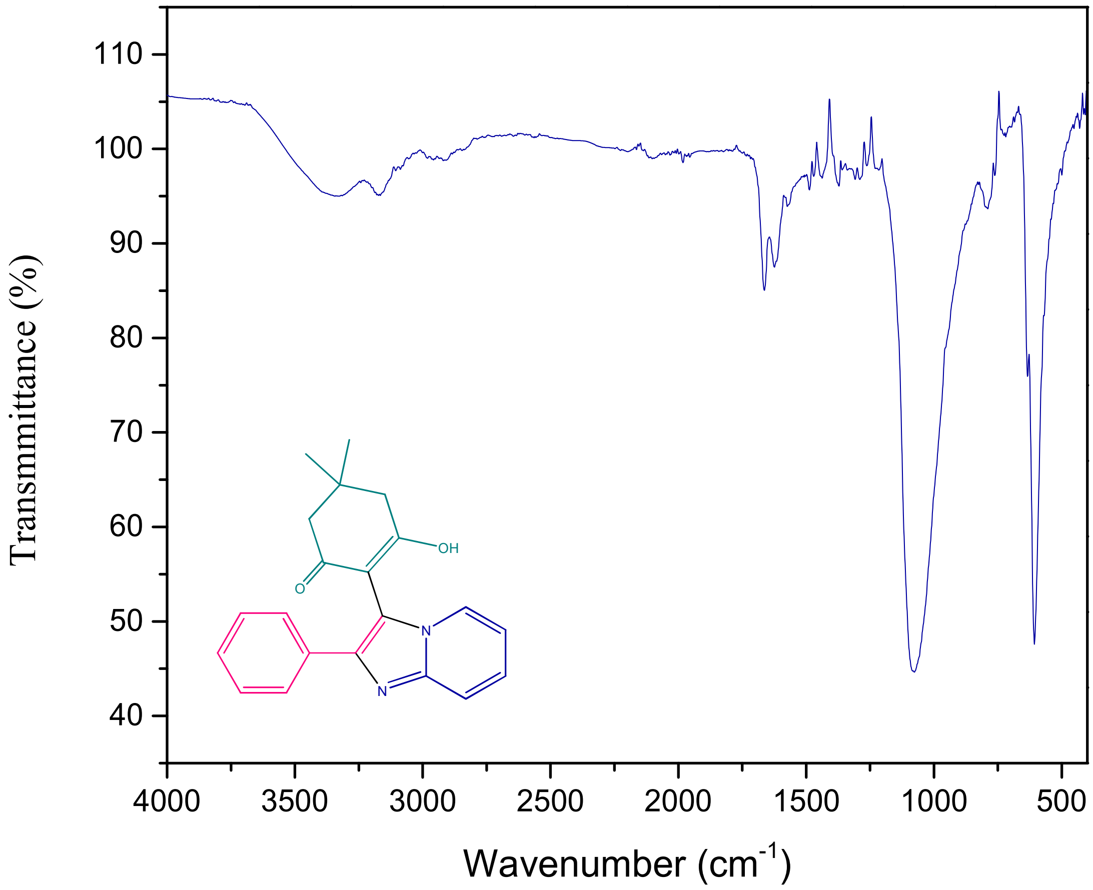

Figure S2. The ^{1}H NMR Spectrum of 3-hydroxy-5,5-dimethyl-2-(2-phenylimidazo[1,2-*a*]pyridin-3-yl)cyclohex-2-enone ***4(a)***

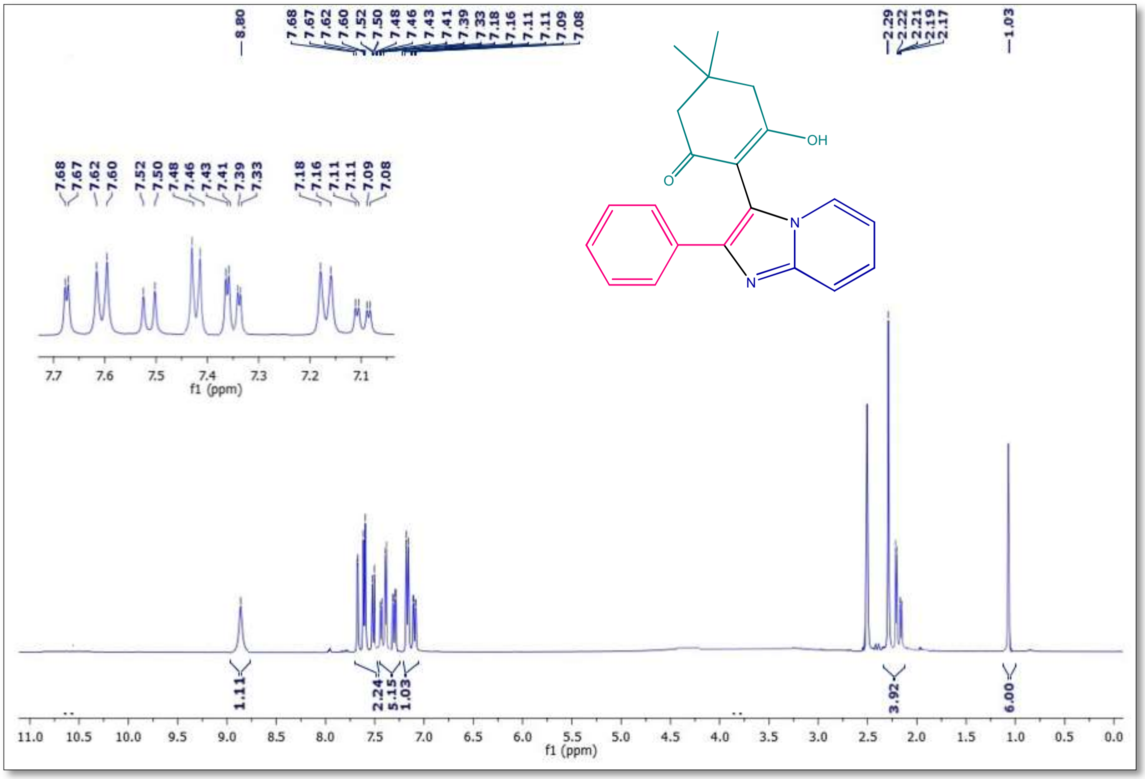

Figure S3. The ^{13}C NMR Spectrum of 3-hydroxy-5,5-dimethyl-2-(2-phenylimidazo[1,2-*a*]pyridin-3-yl)cyclohex-2-enone *4(a)*

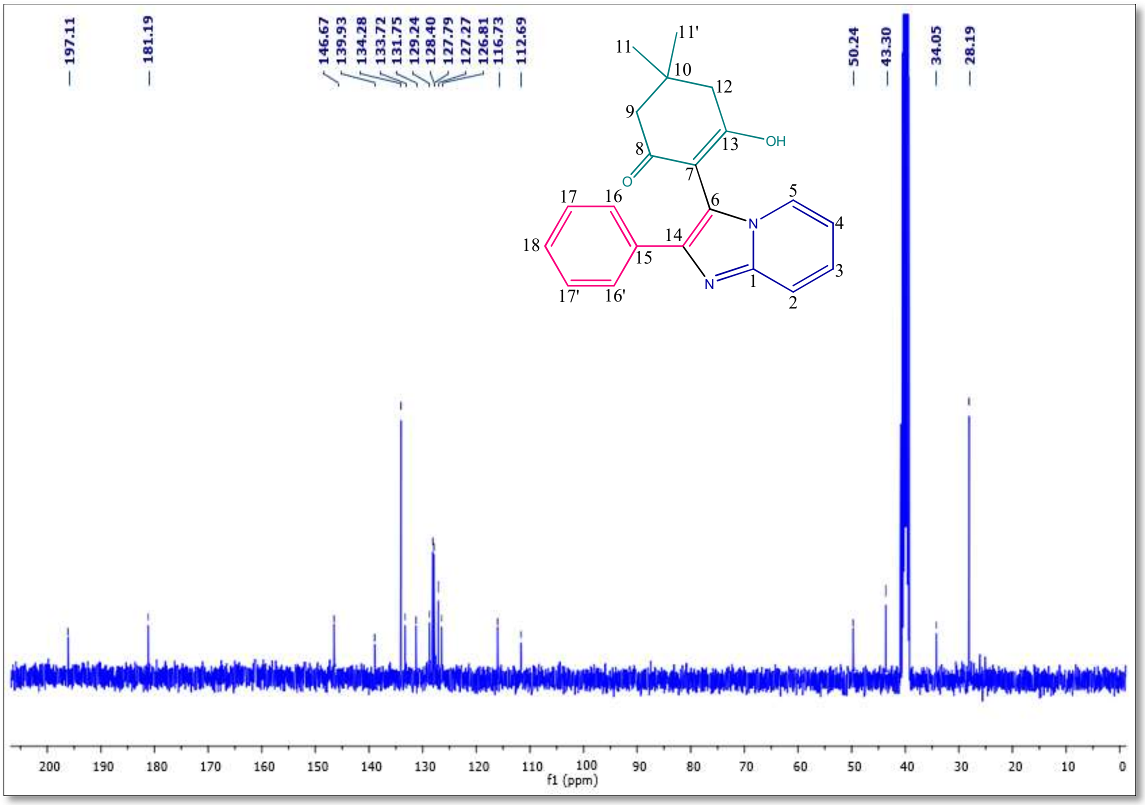

Figure S4. The ESI-MS Spectrum of 3-hydroxy-5,5-dimethyl-2-(2-phenylimidazo[1,2-*a*]pyridin-3-yl)cyclohex-2-enone *4(a)*

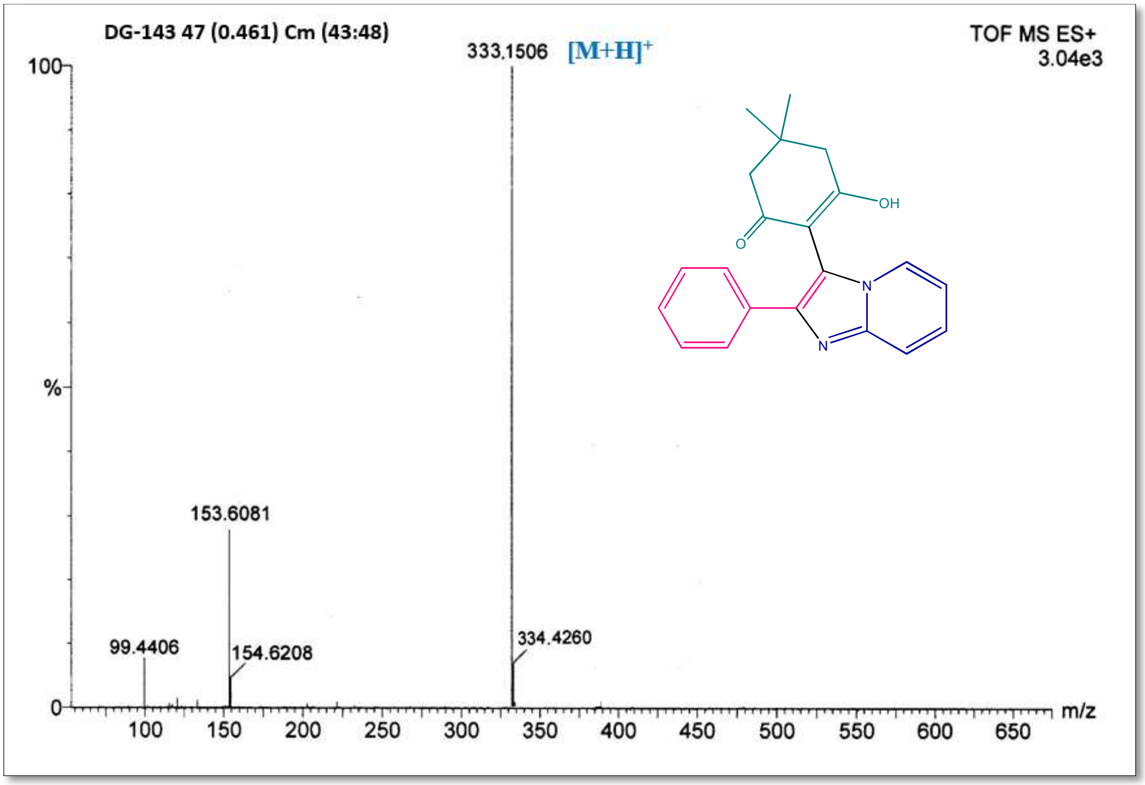

References

1. Geedkar, D.; Kumar, A.; Sharma, P. Multiwalled carbon nanotubes crowned with nickel-ferrite magnetic nanoparticles assisted heterogeneous catalytic strategy for the synthesis of benzo[*d*]imidazo-[2,1-*b*]thiazole scaffolds. *J. Heterocycl. Chem*. **2020**, *57*, 4331−4347.
2. Geedkar, D.; Kumar, A.; Reen, G. K.; Sharma, P. Titania-silica nanoparticles ensemblies assisted heterogeneous catalytic strategy for the synthesis of pharmacologically significant 2,3-diaryl-3,4-dihydroimidazo[4,5-*b*]indole scaffolds. *J. Heterocycl. Chem.* **2020**, *57*, 1963−1973.
3. Geedkar, D.; Kumar, A.; Kumar, K.; Sharma, P. Hydromagnesite sheets impregnated with cobalt−ferrite magnetic nanoparticles as heterogeneous catalytic system for the synthesis of imidazo[1,2-*a*]pyridine scaffolds. *RSC Adv.* **2021**, *11*, 23207−23220.
